# Electrosynthesis of chlorine from seawater-like solution through single-atom catalysts

**DOI:** 10.1038/s41467-023-38129-w

**Published:** 2023-04-29

**Authors:** Yangyang Liu, Can Li, Chunhui Tan, Zengxia Pei, Tao Yang, Shuzhen Zhang, Qianwei Huang, Yihan Wang, Zheng Zhou, Xiaozhou Liao, Juncai Dong, Hao Tan, Wensheng Yan, Huajie Yin, Zhao-Qing Liu, Jun Huang, Shenlong Zhao

**Affiliations:** 1 CAS Key Laboratory of Nanosystem and Hierarchical Fabrication, CAS Center for Excellence in Nanoscience, National Center for Nanoscience and Technology, Beijing, 100190 China; 2 School of Chemical and Biomolecular Engineering, The University of Sydney, Sydney, NSW 2006 Australia; 3 Key Laboratory of Rare Earth Optoelectronic Materials and Devices of Zhejiang Province, College of Optical and Electronic Technology, China Jiliang University, Hangzhou, 310018 China; 4 Department of Mechanical Engineering, University of Aveiro, Aveiro, 3810-93 Portugal; 5 School of Aerospace, Mechanical and Mechatronic Engineering, The University of Sydney, Sydney, NSW 2006 Australia; 6 Beijing Synchrotron Radiation Facility, Institute of High Energy Physics, Chinese Academy of Sciences, Beijing, 100049 China; 7 National Synchrotron Radiation Laboratory, University of Science and Technology of China, Hefei, 230029 China; 8 Institute of Solid-State Physics, Chinese Academy of Sciences, Hefei, 230031 China; 9 School of Chemistry and Chemical Engineering/Guangzhou Key Laboratory for Clean Energy and Materials/Key Laboratory for Water Quality and Conservation of the Pearl River Delta, Ministry of Education, Guangzhou University, Guangzhou, 510006 China

**Keywords:** Electrocatalysis, Chemical engineering, Electrocatalysis

## Abstract

The chlor-alkali process plays an essential and irreplaceable role in the modern chemical industry due to the wide-ranging applications of chlorine gas. However, the large overpotential and low selectivity of current chlorine evolution reaction (CER) electrocatalysts result in significant energy consumption during chlorine production. Herein, we report a highly active oxygen-coordinated ruthenium single-atom catalyst for the electrosynthesis of chlorine in seawater-like solutions. As a result, the as-prepared single-atom catalyst with Ru-O_4_ moiety (Ru-O_4_ SAM) exhibits an overpotential of only ~30 mV to achieve a current density of 10 mA cm^−2^ in an acidic medium (pH = 1) containing 1 M NaCl. Impressively, the flow cell equipped with Ru-O_4_ SAM electrode displays excellent stability and Cl_2_ selectivity over 1000 h continuous electrocatalysis at a high current density of 1000 mA cm^−2^. Operando characterizations and computational analysis reveal that compared with the benchmark RuO_2_ electrode, chloride ions preferentially adsorb directly onto the surface of Ru atoms on Ru-O_4_ SAM, thereby leading to a reduction in Gibbs free-energy barrier and an improvement in Cl_2_ selectivity during CER. This finding not only offers fundamental insights into the mechanisms of electrocatalysis but also provides a promising avenue for the electrochemical synthesis of chlorine from seawater electrocatalysis.

## Abstract (web summary)

Chlor-alkali process plays an important role in the chemical industry. However, large overpotential and low selectivity of currently used catalysts lead to high energy consumption. Here the authors report Ru-O_4_ single site catalysts for chlorination evolution with 1000 h stability at 1000 mA cm^−2^ in a seawater-like environment.

## Introduction

Chlorine plays a pivotal role in the global chemical industries, including in water treatments, organic chemistry, disinfection goods production, and pharmaceutical manufacture^1–3^. Among many, electrocatalytic saturated brine to produce chlorine has been considered as the most efficient and feasible approach. In the past half a century, dimensional stable anode (DSA) consisting of RuO_2_ and TiO_2_ is predominantly used as the electrocatalyst to reduce the energy consumption during the Chlor-alkali process^4^. However, the intrinsic poor electron transfer property and low surface area of the metal oxides greatly limit their electrocatalytic activity as well as mass transfer and diffusion of both reactants and electrolytes^5–8^. Furthermore, the RuO_2_ particles in DSA are highly active for the oxygen evolution reaction (OER), exhibiting a scaling relationship between the CER and OER. This relationship suggests that two reactions are catalyzed on a similar active site of the RuO_2_ or form a common surface intermediate species, unavoidably decreasing the selectivity and activity of the CER^9,10^. Even though the pH of the electrolyte under industrial conditions has been adjusted to 2 to inhibit OER, the selectivity for Cl_2_ in the process of Chlor-alkali over DSA is only ~95%^11^. And, considering the electrocatalysis is an interfacial reaction, the majority of Ru in bulk DSA can not be accessible, resulting in its utilization efficiency being insufficient^12^.

Carbon-supported single-atom catalysts (CS-SACs) have attracted increasing attention in various heterogeneous catalysis and energy-related applications owing to their unique physicochemical properties such as the high atom utilization efficiency (~100%), low-coordination environment of metal centers and distinct structure^13, 14^. Recent experimental and theoretical works suggested that CS-SACs could provide the ideal platform for both performance improvement and catalytic mechanism study^15^. In particular, the component and structure regulation on metal-coordination atom moieties is vital to promote the performance of catalysts and extend their application. Recently, various single atomic sites like M-N_x_, M-P_x_N_y_, and M-S_x_N_y_ with different configurations are fabricated via the coordination engineering strategies and successfully used as highly active and/or selective electrocatalysts for the advanced chemical conversion reactions including the hydrogen/oxygen evolution reactions and CO_2_/oxygen reduction reactions^16–20^. Though atomically dispersed cluster-based catalysts are recently used for electrocatalytic chloride oxidation^21, 22^, the design of single-atom nanomaterials for CER and the related mechanism study has been rarely reported to date^23^.

Herein, the two-dimensional CS-SACs (2D CS-SACs) with a single atomic moiety of Ru-O_4_ (Ru-O_4_ SAM) are successfully synthesized through a thermal reduction approach to in situ anchor the single atoms onto the surface of the oxygen-group enriched ultrathin metal-organic frameworks (MOF) nanosheet derivatives (MOFNDs). And the as-prepared Ru-O_4_ SAM is used as electrocatalysts for CER, exhibiting an extremely low overpotential (30 mV) at 10 mA cm^−2^ and high selectivity (99%) in the 1 M NaCl acidic solution (pH = 1) at room temperature, superior to the commercial DSA (85 mV and 95.5%) and the reported works under identical conditions. Remarkably, the flow cell equipped with the Ru-O_4_ SAM anode possesses excellent stability over 1000 h at a current density of 1000 mA cm^−2^ with Cl_2_ selectivity over 98%. Operando spectroscopy characterizations and density functional theory (DFT) calculations unveil that different from the bulk RuO_2_, the Ru-O_4_ SAM facilitates the direct adsorption of the chlorides to form the M-Cl intermediates, leading to the Gibbs free-energy change of the CER greatly decreased. These findings open the avenue toward the design and construction of high-performance CER electrocatalysts at the atomic level.

## Result and discussion

### Synthesis and structure characterizations of Ru-O_4_ SAM

**Fig. 1:**
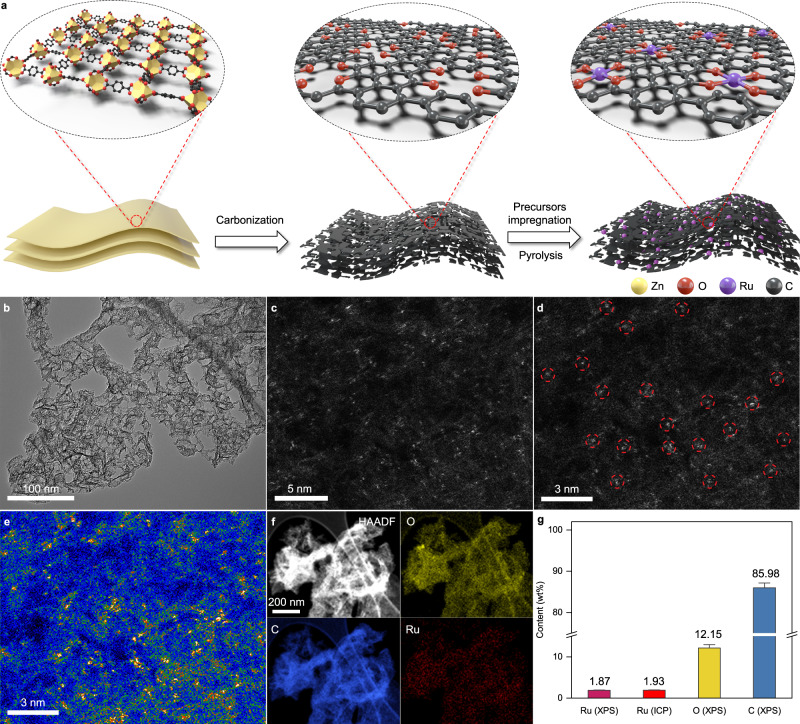
The synthesis strategy and characterizations. **a** Schematic illustration of the synthetic strategy of Ru-O_4_ SAM. Yellow, black, red, and purple spheres represent Zn, C, O, and Ru atoms, respectively. **b** TEM image of Ru-O_4_ SAM. **c**, **d** AC HAADF-STEM image and the enlarged image of Ru-O_4_ SAM. SAs were highlighted by red circles. **e** Corresponding intensity maps of Ru-O_4_ SAM in **d**. **f** EDS mapping images for 2D CS-SACs. **g** Elemental content of Ru-O_4_ SAM, obtained from XPS and ICP-AES.

Figure 1a shows the synthetic schematics of oxygen-coordinated Ru-based single-atom catalysts Ru-O_4_ SAM via a wetness impregnation, followed by pyrolytic progress at 750 °C. The ultrathin MOFNDs are employed as the porous carbon matrix to disperse and immobilize Ru(acac)_3_ due to the large surface area, abundant pores, and excellent permeability (Supplementary Figs. 1 and 2). Also, the in situ formed single atoms during the pyrolysis could be uniformly anchored on the oxygen defects enriched 2D MOFNDs (Supplementary Fig. 3). Subsequently, a series of characterizations including transmission electron microscopy (TEM), X-ray photoelectron spectroscopy (XPS) and inductively coupled plasma atomic emission spectrometry (ICP-AES) were carried out to investigate the physical structure of the resultant samples. The morphology of as-synthesized Ru-O_4_ SAM is well discerned by TEM, in which porous ultrathin structure (Fig. 1b) inherits from the feature of the MOF precursors (Supplementary Fig. 4). High-resolution TEM (HRTEM) and high-angle annular dark-field scanning TEM (HAADF-STEM) images of the as-prepared samples (Supplementary Figs. 5 and 6) confirm no obvious Ru nanoparticles or clusters, which is consistent with the result from X-ray diffraction (XRD) measurement (Supplementary Fig. 7). The monodispersed Ru can be directly observed by the aberration-corrected (AC) HAADF-STEM (Fig. 1c–e and Supplementary Fig. 8). The Ru atoms are confirmed by isolated bright dots in the high-magnification HAADF-STEM image. For comparison, Ru nanoparticles anchored on 2D CS (CS-Ru NPs) are prepared through a similar method. The XRD patterns, TEM images, and XPS demonstrated the successful synthesis of CS-Ru NPs (Supplementary Figs. 9–11). The composition analysis by XPS spectrum (Supplementary Fig. 12) confirms that the obtained Ru-O_4_ SAM is composed of C, O, and Ru without other impurities. The element mapping on the nanosheets (Fig. 1f) suggests the uniform distribution of these elements throughout the entire sample surface. The quantitative measurement by ICP-AES reveals that Ru content in the as-prepared sample is ~1.93 wt% (Fig. 1g), which is very close to the measured values based on XPS (1.87 wt%). And, the average content of C and O in Ru-O_4_ SAM is 85.98 wt% and 12.15 wt%, respectively. Additionally, N_2_ adsorption-desorption isotherm further demonstrates the as-prepared Ru-O_4_ SAM possesses a high specific surface area of 1320 m^2^ g^−1^ (Supplementary Figs. 13 and 14) with the hierarchical pores, benefiting the mass transfer and diffusion of gas evolution evolving reactions.

### Local electronic and atomic structure analysis of Ru-O_4_ SAM

**Fig. 2:**
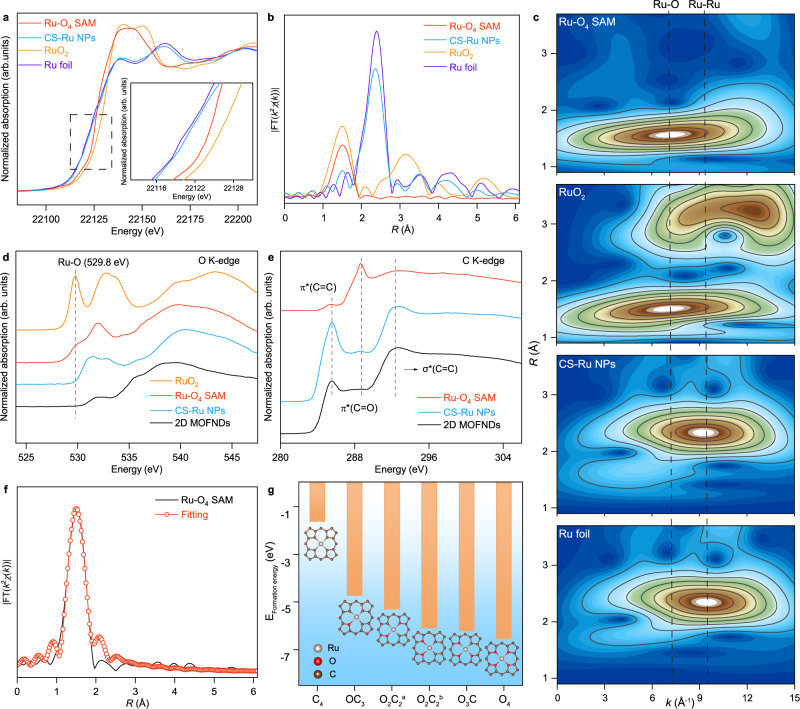
Chemical state and atomic coordination environment of Ru-O_4_ SAM. **a**, **b** Normalized XANES and *k*^2^-weight FT-EXAFS curves of Ru-O_4_ SAM at Ru *K*-edge. **c** WT-EXAFS plots of Ru-O_4_ SAM, RuO_2_, CS-Ru NPs, and Ru foil, respectively. **d**, **e** O *K*-edge and C *K*-edge XANES spectra of Ru-O_4_ SAM. **f** *k*^2^-weight FT-EXAFS fitting curves of Ru-O_4_ SAM at Ru *K*-edge. **g** DFT calculated formation energies of various RuO_*x*_C_y_ (X + Y = 4, X = 1, 2, 3, and 4; *a*, two O atoms are opposite. *b*, two oxygen atoms are adjacent).

To investigate the electronic structure and coordination environment of Ru-O_4_ SAM, the X-ray absorption near edge structure (XANES) and extended X-ray absorption fine structure (EXAFS) spectra were carried out. Ru *K*-edge XANES spectra (Fig. 2a) show the position of the absorption edge and the intensity of the white-line peak of Ru-O_4_ SAM locates between the Ru foil (metallic state) and RuO_2_ (+4) standards. The quantitative linear combination fitting analysis further verifies the chemical valence of Ru in Ru-O_4_ SAM is +3.2. Also, the geometric structure information of Ru-O_4_ SAM at the atomic level is revealed by Fourier-transformed *k*^2^-weighted EXAFS (FT-EXAFS) analysis (Fig. 2b). As shown in Fig. 2b, Ru-O_4_ SAM exhibits only one dominant peak at 1.5 Å caused by the nearest shell coordination of Ru-O bonding. Notably, there is an obvious peak originating from Ru-Ru coordination at 2.3 Å for both Ru foil and CS-Ru NPs while no related signal is observed in the FT-EXAFS spectrum of Ru-O_4_ SAM, indicating that Ru atoms are anchored on the surface of 2D MOFNDs in isolation, in agreement with the HAADF image in Fig. 1d. To provide both *R*- and *k*-space information and discriminate the backscattering atoms, the wavelet transform (WT) analysis of EXAFS spectra was carried out (Fig. 2c). For the pronounced intensity corresponding to the FT-EXAFS peak at 1.5 Å in Fig. 2b, a contour intensity maximum originating from Ru-O scattering is observed at 7.0 Å^−1^ in WT-EXAFS spectra of Ru-O_4_ SAM and RuO_2_. And, compared with the WT signals of Ru foil and CS-Ru NPs, no Ru-Ru coordination can be observed in Ru-O_4_ SAM, suggesting the isolated Ru atoms are immobilized by the O atoms of the carbon framework.

To confirm the Ru-O bonding configuration of Ru single atoms in Ru-O_4_ SAM, we employed synchrotron radiation-based soft XANES measurements to observe the local coordination environment of Ru atoms. XANES is an excellent method for this purpose because of its local structure sensitivity and element specificity. The O *K*-edge spectrum (Fig. 2d) of Ru-O_4_ SAM shows a sharply enhanced peak at 529.8 eV, which can be ascribed to the excitation of O 1*s* core electrons into hybridized states between O 2*p* and Ru 4*d*^24,25^. Meanwhile, three peaks at 285.6, 288.6, and 292.8 eV, which are assigned to the dipole transition of the C 1*s* into 2*p*-derived π*(C=C), π*(C=O), and σ*(C=C), respectively are observed in C *K*-edge spectrum (Fig. 2e)^26^. And, the significantly enhanced peak at 288.6 eV suggests a perturbed bond between C and O, which is likely attributed to a strong chemical interaction occurring between Ru atoms and O atoms on the carbon substrate, demonstrating the formation of C-O-Ru bonds in Ru-O_4_ SAM^27^. The existence of Ru-O-C moiety in Ru-O_4_ SAM deduced from soft XANES spectra is also verified by XPS analysis (Supplementary Figs. 15 and 16). To quantify the structural parameters of Ru-O-C configuration (i.e., bond length and coordination number) the least-square EXAFS fitting was carried out by using Ru-O backscattering path (Fig. 2f, Supplementary Fig. 17, and Supplementary Table 1). The calculated average coordination number of surrounding coordination O atoms is ~3.8 with a bond length of 1.99 Å (Supplementary Table 1). To further validate the proposed structure, DFT calculations (Fig. 2g, Supplementary Figs. 18, 19 and Supplementary Table 2) were conducted by considering the possible models of RuO_x_C_4-x_C_10_/RuO_x_C_4-x_C_12_ (X = 0, 1, 2, 3, and 4). The results show that RuO_4_ (−6.5 eV) is much more energetic favorable than RuO_3_C (−6.2 eV), RuO_2_C_2_^a^ (−5.3 eV), RuO_2_C_2_^b^ (−6.1 eV), RuOC_3_ (−4.7 eV) and RuC_4_ (−1.6 eV), signifying that the Ru-O_4_ moiety is the most stable structure.

### Evaluation of electrochemical activity

**Fig. 3:**
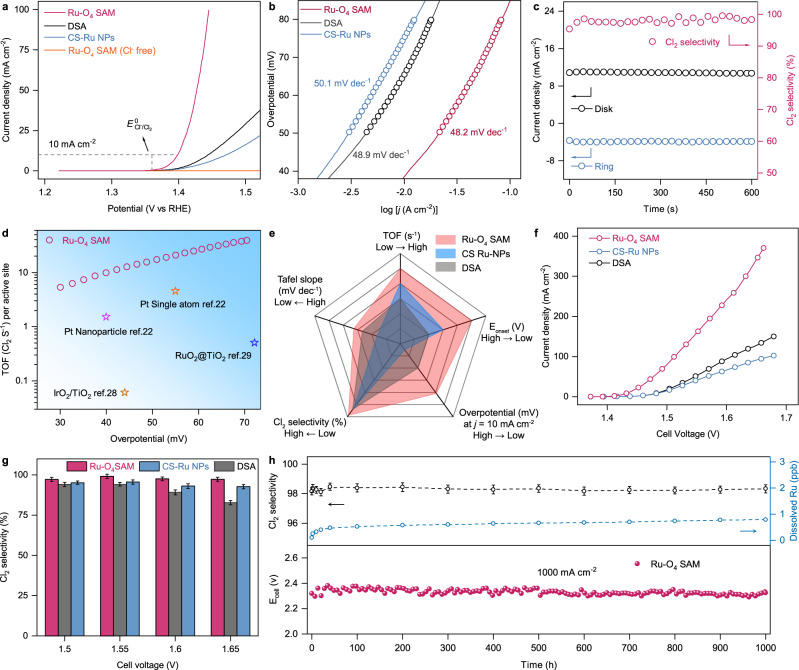
CER electrochemical activity of Ru-O_4_ SAM. **a** Polarization curves of Ru-O_4_ SAM, CS-Ru NPs, and DSA in 1 M NaCl solution with pH of 1 at a scan rate of 5 mV s^−1^ and an electrode rotation speed of 1600 rpm. The polarization curve of Ru-O_4_ SAM in Cl^−^-free environment measured in 1 M NaClO_4_ with a pH of 1. The DSA is measured without electrode rotation. All polarization curves were corrected with 95% *iR* compensation. **b** Tafel plots of Ru-O_4_ SAM, CS-Ru NPS, and DSA in 1 M NaCl solution. **c** Cl_2_ selectivity testing of Ru-O_4_ SAM using rotating ring-disk electrode (RRDE) technique in Ar-saturated 1 M NaCl solution with pH of 1. When a constant potential is applied to the disk electrode for Cl_2_ generation for 600 s, a ring current caused by Cl_2_ reduction is detected immediately. **d** Turnover frequency (TOF) of the Ru-O_4_ SAM calculated based on the loaded Ru atoms at different overpotentials along with some recently reported CER catalysts. **e** Comparison of TOF at an overpotential of 50 mV, onset potential (*E*_onset_), overpotential to reach 10 mA cm^−2^, Tafel slope, and Cl_2_ selectivity. **f** Current densities against cell voltages on Ru-O_4_ SAM, CS-Ru NPs, and DSA using a home-made flow cell system. **g** Corresponding Cl_2_ selectivity of Ru-O_4_ SAM, CS-Ru NPs, and DSA at different cell voltages measured by iodometric titration. **h** Stability test of Ru-O_4_ SAM measured in 1 M NaCl using a home-made flow cell system. The current density of the flow cell is maintained at 1000 mA cm^−2^.

The intrinsic CER performance of Ru-O_4_ SAM, CS-Ru NPs, and DSA was evaluated in a three-electrode H-type cell containing 1 M NaCl solution (pH = 1). The potential of the reference electrode was checked before the CER test (Supplementary Fig. 20), while both rotating ring-disk electrode and iodometric titration were performed to confirm the Cl_2_ formation (Supplementary Video 1 and Supplementary Table 3). Linear sweep voltammetry is employed to record polarization curves of Ru-O_4_ SAM, CS-Ru NPs, and commercial DSA. As Fig. 3a and Supplementary Fig. 21 shown, a sharp increased anodic current response starts from an onset potential (*E*_onset_) (defined as the potential required to reach a current density of 1 mA cm^−2^)^18^ of 1.37 V for Ru-O_4_ SAM, indicating a superior catalytic activity compared with those of CS-Ru NPs (1.40 V) and commercial DSA (1.39 V). On contrary, no obvious current is detected for Ru-O_4_ SAM electrode in Cl^−^-free electrolyte, demonstrating the signal is from CER rather than water oxidation. Besides, the overpotential required to achieve a current density of 10 mA cm^−2^ is another essential parameter for CER performance evaluation. Ru-O_4_ SAM exhibits an extremely low overpotential of ~30 mV at 10 mA cm^−2^, significantly smaller than that of DSA (85 mV), CS-Ru NP_S_ (110 mV), and recently reported CER catalysts under identical conditions (Supplementary Table 4). The electrochemical surface area (ECSA) measurements show the intrinsically improved CER activity on the Ru-O_4_ SAM with 0.02 mA ${{{\mbox{cm}}}}_{{{\mbox{ECSA}}}}^{-2}$ at an overpotential of 50 mV, which is higher than that of DSA (0.01 mA ${{{\mbox{cm}}}}_{{{\mbox{ECSA}}}}^{-2}$) and CS-NPs (0.002 mA ${{{\mbox{cm}}}}_{{{\mbox{ECSA}}}}^{-2}$) (Supplementary Fig. 22). The catalytic kinetic of Ru-O_4_ SAM was further assessed by Tafel plots in 1 M NaCl solution.

As shown in Fig. 3b, the resultant Tafel slope of Ru-O_4_ SAM (48.2 mV dec^−1^) is similar to those of DSA (48.9 mV dec^−1^) and CS-Ru NPS (50.1 mV dec^−1^) within an overpotential range of 50–80 mV, demonstrating that the superior CER activity of Ru-O_4_ SAM is traced to a higher exchange current density. To investigate the selectivity of the Ru-O_4_ SAM, RRDE measurement and iodometric titration were employed (Fig. 3c and Supplementary Table 3). When a constant potential was applied to maintain the current density in disk electrode higher than 10 mA cm^−2^ (~10.8 mA cm^−2^) for 600 s to generate Cl_2_, a ring current of 4.1 mA cm^−2^ caused by Cl_2_ reduction can be detected immediately, representing a high Cl_2_ selectivity of 99% (Fig. 3c and Supplementary Fig. 23)^28^. The superior CER selectivity of as-prepared Ru-O_4_ SAM are further demonstrated at a higher pH value (Supplementary Figs. 24 and 25). The turnover frequency (TOF) values of Ru-O_4_ SAM are calculated based on the loaded Ru atoms. Ru-O_4_ SAM exhibits a TOF value of 17.8 s^−1^ per Ru atom at an overpotential of 50 mV and a production rate of 1.6 mmol cm^−2^ h^−1^ at 1.43 V, which is significantly higher than the recently reported CER catalysts (Fig. 3d, e). In addition, the stability of as-prepared catalysts was examined by chronoamperometry at an initial current density of 10 mA cm^−2^ (Supplementary Fig. 26). After 12 h operation, the current density (10 mA cm^−2^) at Ru-O_4_ SAM electrode retains around 95%, which is better than those of CS-Ru NPs (80%) and DSA (86%).

Also, a home-made flow cell equipped with Ru-O_4_ SAM electrode (Supplementary Fig. 27) was fabricated to evaluate its scalability for chlorine production. As shown in Fig. 3f, the voltage for the flow cell with Ru-O_4_ SAM electrode only requires 1.52 V when it reaches a commercially used current density of 100 mA cm^−2^ in the Chlor-alkali process^29^, which is significantly smaller than those of CS-Ru NPs (1.64 V) and DSA (1.58 V) under the identical conditions. Moreover, the selectivity of the flow cell with Ru-O_4_ SAM electrode can maintain over 97.5% within a wide range of applied potentials (Fig. 3g). And, under a constant current density of 100 mA cm^−2^, only 4.5% cell voltage shift is observed after 100 h continuous electrolysis (Supplementary Fig. 28). Additionally, the morphology and electronic structure of Ru-O_4_ SAM are completely maintained after the long-term test (Supplementary Figs. 29 and 30), substantiating the excellent stability.

The feasibility of practical applications of Ru-O_4_ SAM for industrial-scale Cl_2_ production has been further investigated (Supplementary Fig. 31). As shown in the bottom of Fig. 3h, the fabricated cell requires an initial cell voltage of only 2.32 V to obtain 1000 mA cm^−2^. Moreover, there is no significant decline after ~1000 h of continuous operation. Besides, selectivity is another critical parameter for evaluating the performance of the electrocatalysts. Figure 3h (black dotted circle) indicates the Ru-O_4_ SAM exhibits an excellent Cl_2_ selectivity of over 98% throughout the entire operation period. Inductively coupled plasma mass spectrometry (ICP-MS) measurements were conducted to monitor the dissolved Ru ions in the electrolytes during CER. Figure 3h (blue dotted circle) shows that the concentration of dissolved Ru in Ru-O_4_ SAM is lower than 1 ppb even after 1000 h of electrolysis, verifying its practical application under high current density. As comparison, the performance of DSA for large-scale Cl_2_ production was also obtained. As shown in Supplementary Fig. 32, the initial voltage (2.9 V) for the flow cell with the DSA shifts to 3.1 V after 1000 h electrocatalysis under a given current density of 1000 mA cm^−2^ whereas no obvious voltage change can be observed for the Ru-O_4_ SAM electrode. And, Supplementary Fig. 32 (black dotted circle) shows that the selectivity of DSA can maintain only around 94% Cl_2_ throughout the entire performance period. The ICP-MS measurement (blue dotted circle in Supplementary Fig. 32) indicates that the concentration of dissolved Ru in DSA is ~ 5 ppb after 1000 h of electrolysis.

To explore the structure-stability relationship, we opted to perform ex-situ X-ray absorption spectroscopy (XAS) characterizations of the post-reacted Ru-O_4_ SAM. Supplementary Fig. 33 show the Ru *K*-edge in the XANES spectra of the samples collected at the initial stage, 100 h electrolysis, and 1000 h electrolysis. As the operation time increases, the position of the Ru *K*-edge for the sample after 1000 h electrocatalysis is only 0.8 eV higher than that of the pristine sample, indicating a slightly elevated oxidation state of the Ru during the CER reaction. Notably, the chemical valence of Ru (+3.5) for the post-reacted sample is much lower than that of RuO_2_ (+4). It is well established that the Ru will be dissolved when its oxidation state is higher than Ru^4+^. Therefore, it can be concluded that the stability of Ru-O_4_ SAM is ascribed to its robust electronic structure. Besides, the geometric structure of Ru-O_4_ SAM before and after the reaction was investigated by FT-EXAFS (Supplementary Fig. 34a–c). As demonstrated in the FT-EXAFS spectrum of the Ru-O_4_ SAM, all three samples displayed a single dominant peak at ~1.5 Å, which is attributed to the nearest shell coordination of Ru-O bonds. No evident peak arising from Ru-Ru coordination at 2.3 Å is observed in the FT-EXAFS spectrum of the Ru-O_4_ SAM, demonstrating that the Ru atoms remain atomically dispersed and unaggregated during the CER process. To determine the structural parameters of the Ru-O configuration, a least-square EXAFS fitting was performed using the Ru-O backscattering path. The calculated average coordination number of surrounding O atoms was approximately 3.8 and 3.9 for the samples after 100 h and 1000 h of operation, respectively. The comprehensive fitting parameters can be found in Supplementary Table 5. In addition, AC HAADF-STEM images of post-CER samples confirm the absence of prominent Ru nanoparticles or clusters (Supplementary Fig. 34d–f). The monodispersed Ru atoms can be directly visualized as isolated bright dots. Therefore, it can be concluded that the excellent stability of Ru-O_4_ SAM is attributed to its robust electronic and geometric structure.

### Operando characterizations and DFT calculations

**Fig. 4:**
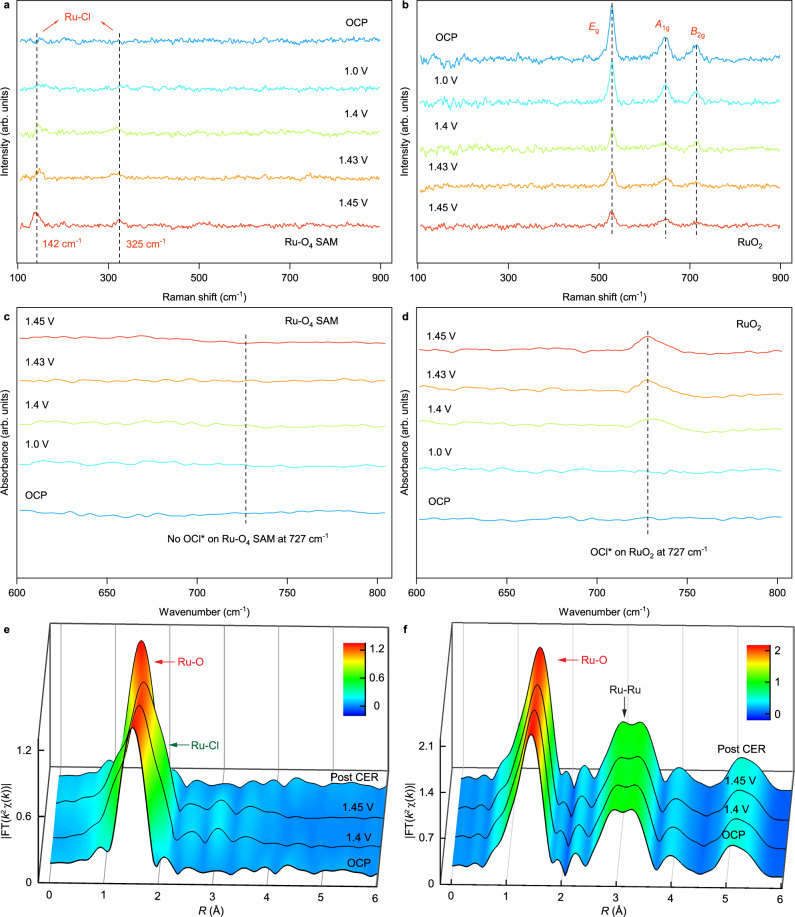
Operando characterization. **a**, **b** In situ Raman spectra collected on (**a**) Ru-O_4_ SAM and (**b**) RuO_2_ electrode from OCP to 1.45 V vs RHE in 1 M NaCl with pH of 1. **c**, **d** In situ SR-FTIR spectra collected on (**c**) Ru-O_4_ SAM and (**d**) RuO_2_ electrode from OCP to 1.45 V vs RHE in an acidic solution (pH = 1) with 1 M NaCl. **e**, **f** Comparison of Ru *K*-edge *k*^2^-weighted FT-EXAFS signals (**e**) Ru-O_4_ SAM and (**f**) RuO_2_ electrode recorded at different potentials.

To explore the origin of the high electrocatalytic activity and possible reaction pathway of Ru-O_4_ SAM during CER, in situ Raman and synchrotron Fourier transform infrared (SR-FTIR) measurements were conducted. Raman spectra obtained at OCP (Fig. 4a, b) show there is no detectable signal for Ru-O_4_ SAM and RuO_2_ samples at 142 and 325 cm^−1^. Interestingly, a pair of peaks gradually appear in the Raman spectra of Ru-O_4_ SAM with the applied potential increased (Fig. 4a), while no signal can be monitored for the RuO_2_ sample (Fig. 4b). Then, we utilized density DFT calculations to verify the Raman bands for the potential configuration. Supplementary Table 6 indicates that the Raman band associated with the Cl^−^ adsorption in the Cl-Ru-O_4_ configuration aligns with the newly detected peak (142 and 325 cm^−1^) in the in situ Raman spectra, which is consistent with the literature^30^. This suggests that the intermediate species originates from the direct adsorption of Cl^−^^31, 32^. On the contrary, the in situ Raman spectra of RuO_2_ possess three peaks at 530, 647, and 714 cm^−1^, which correspond to the *E*_g_, *A*_1g_, and *B*_2g_ models of nanocrystalline RuO_2_, respectively^33^. And, the intensity of *E*_g_, *A*_1g_, and *B*_2g_ bands of RuO_2_ is decreased owing to the formation of O_ot_Cl* intermediate, suggesting an indirect reaction pathway^34, 35^. And, the in situ SR-FTIR spectra (Fig. 4c, d) show the peak originated from O_ot_Cl* stretching at 727 cm^−1^ appears at the RuO_2_ electrode when the applied potential rises to 1.4 V, and the signal is stronger and stronger as the applied bias increases, which is consistent with the results in situ Raman^36, 37^.

Subsequently, operando Ru *K*-edge XAS spectroscopy measurement was performed to detect local electronic and atomic structural changes of the Ru sites inside Ru-O_4_ SAM during the electrocatalytic CER. As shown in Supplementary Figs. 35 and 36, the absorption edge gradually shifts to the higher energy position as the applied potential increases, suggesting a higher oxidation state of Ru formed. And, the Ru *K*-edge shift for Ru-O_4_ SAM is more obvious than that of the RuO_2_ electrode under the same applied potential, indicating the higher activity of Ru-O_4_ SAM during CER^38^. EXAFS and the best-fit analysis in two *k* space (*k*^2^ and *k*^3^) (Fig. 4e, Supplementary Figs. 37–41 and Supplementary Tables 7 and 8) indicate Ru sites in Ru-O_4_ SAM well maintain their original coordination environment with 4 O coordination under OCP conditions. When the applied potential is raised to 1.4 V and 1.45 V, the first coordination shell of Ru has significantly changed, with a new characteristic Ru-Cl peak in the larger bond distance (red area in Supplementary Fig. 37) side of the Ru-O coordinate peak (blue area in Supplementary Fig. 37) and the formed new peak will be more obvious stronger as the applied potential increases. On the contrary, only the peak from the Ru-O coordination is observed in the EXAFS of RuO_2_ and no obvious shift even the applied potential up to 1.45 V (Fig. 4f, Supplementary Fig. 42, and Supplementary Table 9). To demonstrate the reversibility of the formed structure in the catalytic state, we recorded the XAFS spectra of Ru-O_4_ SAM and RuO_2_ after CER. XAFS spectra (Fig. 4e, f, Supplementary Figs. 37 and 42) show the electronic structure of Ru in both Ru-O_4_ SAM and RuO_2_ could back to the original state once the applied bias is removed. EXAFS analysis indicates the formed Cl-Ru peak in Ru-O_4_ SAM will disappear and the coordination structure of Ru will recover after CER, suggesting the structural reversibility of Ru-O_4_ SAM. Additionally, we conducted electrochemical approach to demonstrate the proposed mechanism on Ru-O_4_ SAM and commercial RuO_2_. Supplementary Fig. 43 displays the cyclic voltammetry (CV) curves over Ru-O_4_ SAM and commercial RuO_2_ in 1 M NaCl electrolyte. A noticeable cathodic peak (P1) appears at ~1.36 V vs RHE, which is close to the reversible Cl_2_/Cl^−^ electrode potential (*E*_CER_ = 1.36 V vs RHE). The P1 is ascribed to the directly reduction of Cl_2_. In contrast, the CV curve for commercial RuO_2_ exhibits a distinct cathodic peak (P2) located at ~1.08 V vs RHE, which is noticeably offset from the *E*_eq_ (Cl_2_/Cl^−^). According to relevant literature^39,40^, this peak is ascribed to the reduction of Cl_2_ on the oxygen sites that are adsorbed on the electrode surface. Together with the operando Raman, SR-FTIR, XAS analysis, and electrochemical investigation, it is concluded that different from the indirect adsorption mechanism on RuO_2_ to form the *O_ot_Cl intermediate, Cl can directly adsorb on the Ru sites in Ru-O_4_ SAM during CER.

To further elucidate the reaction mechanism and activity origin of Ru-O_4_ SAM, DFT calculations were carried out. Two possible configurations of RuO_4_C_10_ and RuO_4_C_12_ were built based on EXAFS fitting and calculated formation energy results (Fig. 2g, Supplementary Figs. 18, 19, 44 and 45). The rutile RuO_2_ (110) facet is modeled for comparison (Supplementary Fig. 46), where terraces expose a fully coordinated bridge ruthenium site (Ru_BRI_) and a coordinatively unsaturated site (Ru_CUS_) with fivefold coordination^41, 42^. Subsequently, we constructed all the probable adsorbate structures under CER process for Ru-O_4_ SAM and RuO_2_ based on our operando experiment results in Fig. 4 (Supplementary Figs. 47–53). For Ru-O_4_ SAM, Cl^−^ directly adsorbate on the single Ru active site is determined as the most probable adsorbate structure (*Cl) during CER process (Supplementary Figs. 47 and 48)^43^. In contrast, for the typical rutile RuO_2_ (110) structure, the in situ results revealed that the adsorption of chlorine on the oxygen bonded on-top of Ru_CUS_ atoms (O_ot_) surface forming *OCl adsorbate structure was the most possible adsorbate structure during CER, in good agreement with the proposed results in the literature (Supplementary Figs. 49 and 50)^9, 10, 35^. It should be noted that, despite our operando experiments have already directly observed the most possible adsorbate structure of Ru-O_4_ SAM moiety during CER process is *Cl, we also modeled the *OCl adsorbed structure of Ru-O_4_ moiety during CER to have a more comprehensive theoretically evaluation (Supplementary Figs. 51 and 52).

**Fig. 5:**
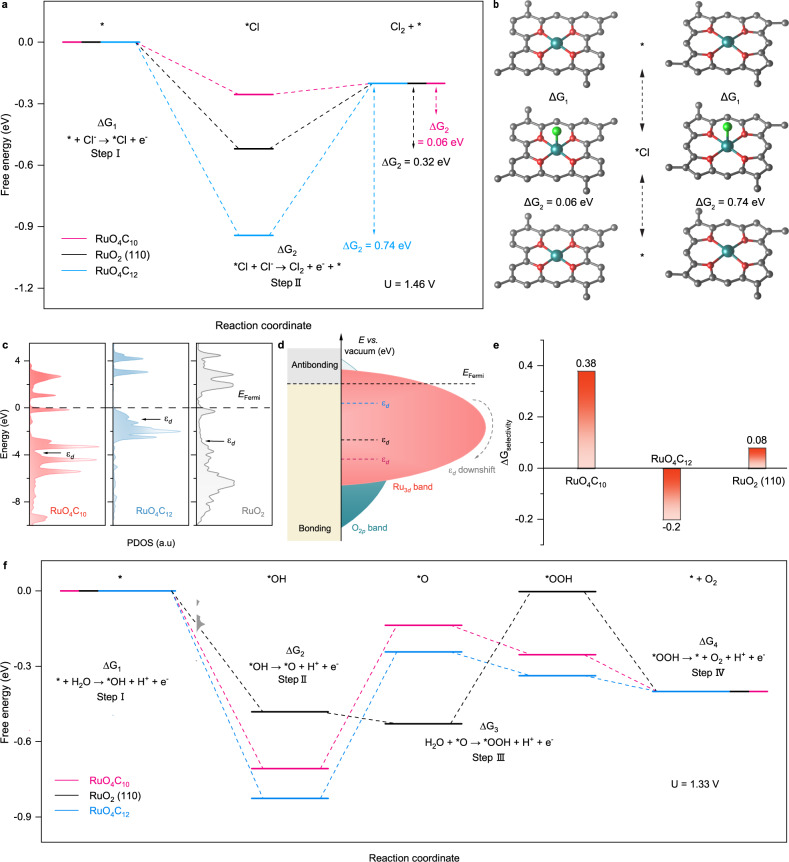
Catalytic mechanism study of Ru-O_4_ SAM and RuO_2_ (110) for the CER. **a** Gibbs free-energy diagram for CER over RuO_4_C_10_, RuO_4_C_12_, and RuO_2_ (110). **b** Proposed CER reaction path on RuO_4_C_10_ and RuO_4_C_12_. Considering the equilibrium potential of the CER U = 1.36 V, at zero overpotential. **c** Calculated Ru projected density of states for RuO_4_C_10_, RuO_4_C_12_, and RuO_2_ (110). **d** *d* band center (ε_d_) shift of RuO_4_C_10_ (red dash line), RuO_4_C_12_ (blue dash line), and RuO_2_ (110) (black dash line). **e** Differences of theoretical overpotential between CER and OER of the RuO_4_C_12_, RuO_4_C_10_, and RuO_2_ (110). **f** Gibbs free-energy diagram for OER over RuO_4_C_10_, RuO_4_C_12_, and RuO_2_ (110). Considering the equilibrium potentials of the OER and CER are U = 1.23 V and U = 1.36 V, at zero overpotential.

The relative free-energy diagrams for possible adsorbate structures (*Cl, and *OCl) for CER over RuO_4_C_10_, RuO_4_C_12_, and RuO_2_ (110) catalysts were further calculated under U = 0 V and U = 1.46 V (Fig. 5a, b, and Supplementary Figs. 53–55). The calculated Gibbs free-energy changes along two reaction steps (ΔG_1_ and ΔG_2_) illustrate that the second step accompanying the formation of molecular Cl_2_ through the recombing process of *Cl/*OCl with another Cl^−^ (*Cl/*OCl +Cl^−^ → */*O + Cl_2_ + e^−^) is the potential-determining step (PDS) for both Ru-O_4_ SAM (ΔG_2_ = 0.06 eV for RuO_4_C_10_ and ΔG_2_ = 0.74 eV for RuO_4_C_12_) and rutile RuO_2_ (110) structures (ΔG_2_ = 0.32 eV) due to their higher Gibbs free-energy difference (Fig. 5a)^44, 45^. RuO_4_C_10_ is identified as the most probable structure for the CER with *Cl intermediates owing to its lowest Gibbs free-energy change of PDS (ΔG_2_ = 0.06 eV) with a thermodynamic overpotential (${{{{{{\rm{\eta }}}}}}}_{{{{{{\rm{TD}}}}}}\left({{{{{\rm{CER}}}}}}\right)}$) of 0.16 V (Fig. 5b). As shown in Fig. 5a, b, RuO_4_C_12_ displays the lowest Gibbs free-energy change for direct adsorption of Cl^−^ in step 1 (* + Cl^−^ → *Cl + e^−^), suggesting the strong adsorption between Cl^−^ and Ru atoms. However, the strongly absorbed intermediates on the surface of the RuO_4_C_12_ result in a substantially increased Gibbs free-energy difference in the second step (ΔG_2_ = 0.74 eV), with a high theoretical overpotential of 0.84 V. In contrast, RuO_4_C_10_ with Cl^−^ directly adsorption exhibits the balanced Gibbs free-energy change between step 1 and step 2 (Fig. 5b). The optimized Gibbs free-energy difference enables *Cl to be easily recombing another Cl^−^ in bulk electrolyte and disporting from the active centers. The theoretical calculation results are consistent with the experimental conclusion from in situ XAS that Cl^−^ directly adsorbed on atomically dispersed Ru atoms in Ru-O_4_ SAM.

To further investigate the binding strengths of the absorbates on the samples, partial density of states (PDOS) calculations were performed. Figure 5c shows the *d* band center (*ε*_d_) values for RuO_4_C_10_, RuO_4_C_12_, and rutile RuO_2_ (110) are −3.83 eV, −0.98 eV, and −2.85 eV, respectively. The more negative *ε*_d_ indicates the weaker chemical bond between intermediate species and Ru sites, thereby leading to lower binding energy and Gibbs free-energy change during CER (Fig. 5d and Supplementary Fig. 56)^46^. Therefore, it can be concluded that RuO_4_C_10_ moiety has advantages in balancing the binding strength of adsorbates and promoting the quick release of molecular Cl_2_^47,48^.

To evaluate the CER selectivity over RuO_4_C_10_, RuO_4_C_12_, and rutile RuO_2_ (110) catalysts, the Gibbs free-energy changes of their OER process were investigated (Fig. 5e, f). As illustrated in Fig. 5f and Supplementary Figs. 57–60, the adsorption-free energies of *O, *OH, and *OOH were calculated. The *O formation step (*OH → *O + H^+^ + e^−^) is defined as the PDS for both RuO_4_C_10_ and RuO_4_C_12_ catalysts, whereas the PDS for rutile RuO_2_ (110) is the *OOH formation (*O + H_2_O → *OOH + H^+^ + e^−^). The theoretical OER overpotential on the surface of RuO_4_C_10_, RuO_4_C_12_, and rutile RuO_2_ (110) are 0.67 V, 0.77 V, and 0.63 V, respectively, indicating the maximal inhibition of RuO_4_C_12_ towards OER. As we know, the selectivity of a catalyst is evaluated by the linear scaling relationship between OER and CER. To directly measure the CER selectivity, we calculated the difference between the thermodynamic overpotential of OER and CER, which can be defined as $\Delta {{{{{{\rm{G}}}}}}}_{{{{{{\rm{Selectivit}}}}}}}={{{{{{\rm{\eta }}}}}}}_{{{{{{\rm{TD}}}}}}({{{{{\rm{OER}}}}}})}-{{{{{{\rm{\eta }}}}}}}_{{{{{{\rm{TD}}}}}}\left({{{{{\rm{CER}}}}}}\right)}-0.13$. As shown in Fig. 5e, OER and CER gap for RuO_4_C_10_ moiety is 0.38 V, much higher than that of RuO_4_C_12_ (−0.2 V) and RuO_2_ (0.08 V), signifying a superior CER selectivity.

In summary, the 2D CS-SACs with Ru-O_4_ SAM are prepared as highly efficient catalysts for the electrosynthesis of chlorine in a low chloride concentration solution. Impressively, the flow cell equipped with the Ru-O_4_ SAM exhibits extremely low overpotential and excellent stability over 1000 h at a current density of 1000 mA cm^−2^ with Cl_2_ selectivity over 98% in simulated seawater media, displaying a huge potential for practical application. Moreover, the well-defined atomic architecture of Ru-O_4_ SAM allows us to explore the origin of the high electrocatalytic activity. Operando characterizations combined with computational analysis reveal the as-prepared Ru-O_4_ SAM facilitates the formation of Cl*-Ru intermediates through direct chloride adsorption, which benefits the catalytic performance enhancement in both activity and selectivity. These findings open an avenue toward the design and construction of high-performance CER electrocatalysts at the atomic level.

## Methods

### Chemicals

Ruthenium (III) acetylacetonate (Ru(acac)_3_), ruthenium (IV) oxide, zinc chloride (ZnCl_2_), sodium chloride (NaCl), sodium hydroxide (NaOH), potassium iodide (KI), sodium perchlorate (NaClO_4_), sodium thiosulfate (Na_2_S_2_O_3_), hydrochloric acid (HCl), perchloric acid (HClO_4_), benzene-1,4-dicarboxylic acid (BDC), triethylamine (TEA), ethanol, *N, N*-dimethylformamide (DMF), Nafion solution (5 wt%) were bought from Sigma-Aldrich. Commercial dimensionally stable anode (DSA) was bought from Baoji Zhiming Special Metal Co., LTD (China). The deionized (DI) water was produced using a Millipore Milli-Q grade, with a resistivity of 18.2 MΩ cm. All chemicals were used directly without further purification.

### Synthesis of Zn MOFs nanosheets (Zn-BDC)

First, a mixture solution was made by adding DMF (64 mL), ethanol (4 mL), and DI water (4 mL) in a 100 mL polytetrafluoroethylene (PE) tube. Then, amount of BDC (0.25 g) was added to the above-mixed solution under ultrasonication for 30 min. Subsequently, 0.21 g ZnCl_2_ salts were dissolved into the solution. Afterward, 1.6 ml TEA was injected into the solution with continuous 8 h ultrasonication (40 kHz) under ambient conditions. Finally, the product was obtained via centrifugation, washed with ethanol 5 times, and dried under vacuum at 60 °C for overnight.

### Synthesis of 2D MOFNDs

The as-synthesized Zn-BDC was heated to 950 °C with a heating rate of 5 °C min^−1^ and maintained for 3 h under an argon atmosphere in a tube furnace. After the tube furnace was naturally cooled to room temperature, the calcined product was mixed with 1 M HCl, and the mixture was stirred for 12 h at room temperature. Finally, the suspension was filtered and washed with excessive amounts of DI water until the filtrate reached a pH of 7 and dried under vacuum at 60 °C for overnight.

### Synthesis of Ru-O_4_ SAM

50 mg 2D MOFNDs were uniformly dispersed in DI water, followed by the addition of 50 ml Ru(acac)_3_ solution (3 wt%). After overnight stirring, the suspension was filtered and washed with DI water and ethanol for five times and dried under vacuum at 60 °C for overnight. Finally, the products were heated to 750 °C with a heating rate of 2 °C min^−1^ and maintained for 3 h under argon atmosphere in a tube furnace. After cooling, the Ru-O_4_ SAM was obtained and directly used as the catalysts without further treatment. The synthesis process for CS-Ru NP was the same as that of Ru-O_4_ SAM, except that the concentration of Ru(acac)_3_ was changed to 6 wt%.

### Characterizations

XRD patterns were obtained using a PANalytical X’Pert diffractometer with a Cu Kα source (*λ* = 1.5406 Å). Scanning electron microscopy (SEM) images were observed on a Zeiss Ultra Plus microscope. TEM and HAADF-STEM images were performed on an FEI Themis-Z microscope at 200 kV. XPS survey was carried out on a Thermo Fisher K-Alpha+ spectrometer equipped with an Al Kα source (1486.3 eV). N_2_ adsorption-desorption isotherms were obtained on an Autosorb iQ gas sorption analyzer (Quantachrome Instruments). ICP-AES was performed on a Varian Vista Pro instrument.

### Electrochemical measurements

The catalysts ink was prepared using a mixture of 2.5 mg catalysts, 300 μl DI water, 700 μl ethanol, and 50 μl 5 wt% Nafion solution. Then, the slurry was ultrasonically dispersed for 2 h. 5 μl catalysts ink was deposited onto a glassy carbon disk (5.5 mm inside diameter), which was used as the working electrode with a loading of 0.05 mg cm^−2^.

The electrochemical measurements were carried out by using an electrochemical workstation (CHI 760E, CH Instrument) at room temperature and atmospheric pressure. The test is performed in an H-type cell with three electrodes using prepared catalysts, Pt mesh, and Ag/AgCl electrode (saturated KCl) as work electrode, counter electrode, and reference electrode, respectively. The H-type cell was separated into two compartments by Nafion 324 membrane. All the potential measured was calibrated into reversible hydrogen electrode (RHE) by using the below equation:

1 $${{\it{E}}}_{{{{{{\rm{RHE}}}}}}}={{\it{E}}}_{{{{{{\rm{Ag}}}}}}/{{{{{\rm{AgCl}}}}}}}+0.197{{{{{\rm{V}}}}}}+0.0591\times {{{{{\rm{pH}}}}}}$$

where the pH was measured by the pH meter (PHS-3E, Yoke Instrument). Prior to the electrochemical performance test, pure argon gas was continuously purged into the electrolyte for 30 min to remove any dissolved oxygen gas. Cyclic voltammetry measurement was conducted between 1.2 V and 1.5 V at a scan rate of 10 mV s^−1^ to obtain stable curves. The polarization curves for the prepared catalyst were measured at a scan rate of 5 mV s^−1^ with a rotation speed pf 1600 rpm. All the results for the polarization curves were corrected with 95% *iR* compensation, where *i* and *R* represent the current and resistance, respectively. The resistance (~6.8 Ω) was determined via open-circuit voltage. The equilibrium potential of CER was determined to ~1.36 V vs RHE based on the Nernst equation^22, 23, 47^.

The standard reduction potential for CER: ${{{{{{\rm{E}}}}}}}_{{{{{{\rm{CER}}}}}}}^{0}=1.358{{{{{\rm{V\; vs}}}}}}.{{{{{\rm{SHE}}}}}}.$ However, the reduction potentials also can be affected by temperature. Thus, it can be modified by the following equation:

2 $${{{\mbox{E}}}}_{{{\mbox{CER}}}}^{0}{{\mbox{=}}}1{{\mbox{.}}}358\,{{\mbox{V}}}-\left(0{{\mbox{.}}}001248\times \frac{\partial {{\mbox{E}}}}{\partial {{\mbox{T}}}}\right)\times \left({{\mbox{T-}}}298{{\mbox{.}}}15{{\mbox{K}}}\right){{{{{\rm{vs}}}}}}.{{{{{\rm{SHE}}}}}}$$

The equilibrium potential of CER under the experimental conditions (1 M NaCl, with continuously bubbling pure argon gas during experiment) can be calculated based on the Nernst equation.

3 $${{{\mbox{E}}}}_{{{\mbox{CER}}}}\text {=} 	1{{\mbox{.}}}358{{\mbox{V-}}}\left(0{{\mbox{.}}}001248\times \frac{\partial {{\mbox{E}}}}{\partial {{\mbox{T}}}}\right)\times \left({{\mbox{T}}}-298{{\mbox{.}}}15{{\mbox{K}}}\right)\\ 	-\left(\frac{{{\mbox{RT}}}}{{{\mbox{F}}}}\right)\times {{{{\mathrm{ln}}}}} {{{{{\rm{\alpha }}}}}}\left({{{\mbox{Cl}}}}^{-}\right)+\left(\frac{{{\mbox{RT}}}}{2{{\mbox{F}}}}\right)\times {{{{\mathrm{ln}}}}}{{{{{\rm{\alpha }}}}}}\left({{{\mbox{Cl}}}}_{2}\right){{{{{\rm{vs}}}}}}.{{{{{\rm{SHE}}}}}}$$

All the reference electrodes have been converted into RHE through the whole manuscript. The equilibrium potential of CER thus is shown below:

4 $${{{{{{\rm{E}}}}}}}_{{{{{{\rm{CER}}}}}}}=	1.358{{{{{\rm{V}}}}}}-\left(0.001248\times \frac{\partial {{{{{\rm{E}}}}}}}{\partial {{{{{\rm{T}}}}}}}\right)\times \left({{{{{\rm{T}}}}}}-298.15{{{{{\rm{K}}}}}}\right)-\left(\frac{{{{{{\rm{RT}}}}}}}{{{{{{\rm{F}}}}}}}\right)\times {{{{{\rm{ln}}}}}}{{{{{\rm{\alpha }}}}}}\left({{{{{{\rm{Cl}}}}}}}^{-}\right) \\ 	+\left(\frac{{{{{{\rm{RT}}}}}}}{2{{{{{\rm{F}}}}}}}\right)\times {{{{{\rm{ln}}}}}}{{{{{\rm{\alpha }}}}}}\left({{{{{{\rm{Cl}}}}}}}_{2}\right)+\left(\frac{{{{{{\rm{RT}}}}}}{{{{{\rm{ln}}}}}}10}{{{{{{\rm{F}}}}}}}\times {{{{{\rm{pH}}}}}}\right){{{{{\rm{vs}}}}}}.{{{{{\rm{RHE}}}}}}$$

Where *T*, *R*, and *F* are the temperature (*k*), gas constant (8.314 J K^−1^ mol^−1^), and Faraday constant (96485.3 C mol^−1^). The activity of chloride α(Cl^−^) is determined as 1 since 1 M NaCl solution is used as the electrolyte during experiment. The partial pressure of Cl_2_ is 0.01 based on the previous literature and the experimental condition (continuously bubbling pure argon gas during experiment)^49^. The pH is adjusted to 1 by few dropping HClO_4_ and measured by the pH meter (PHS = 3E, Yoke Instrument). Thus, The CER equilibrium potential is determined as *E*_CER_ = 1.36 V vs RHE.

The Cl_2_ selectivity for the prepared catalysts was measured by RRDE and iodometric titration methods^29, 50^. For RRDE measurements, the Cl_2_ selectivity was calculated through the equation below:

5 $${{{{{{\rm{Cl}}}}}}}_{2}\left(\%\right)=100\times 2\times \frac{{{\it{I}}}_{{{{{{\rm{ring}}}}}}}/{\it{N}}}{{{\it{I}}}_{{{{{{\rm{disk}}}}}}}+{{\it{I}}}_{{{{{{\rm{ring}}}}}}}/{\it{N}}}$$

Where *N* is the calibrated collection coefficient (~0.38), *I*_ring_ is the collected current from the Pt ring at a fixed potential of 0.95 V, and *I*_disk_ is the obtained current from the disk electrode. For iodometric titration methods, chronoamperometry (CA) was performed at a current density of 10 mA cm^−2^ for 200 s. Then, 5 ml anodic electrolyte was collected and transferred into a 50 ml flask containing large-excessed KI. 0.01 M Na_2_S_2_O_3_ was used to titrate the solution with starch as the indicator. The Cl_2_ selectivity can be calculated by following equation:

6 $${{{{{{\rm{Cl}}}}}}}_{2}\left(\%\right)=100\times \frac{{{{{{\rm{Experiement}}}}}}\,{{{{{\rm{generated}}}}}}\,{{{{{{\rm{Cl}}}}}}}_{2}}{{{{{{\rm{Theoretical}}}}}}\; {{{{{\rm{generated}}}}}}\,{{{{{{\rm{Cl}}}}}}}_{2}}=\frac{\frac{0.01\,{{{{{\rm{M}}}}}}\times {{{{{{\rm{V}}}}}}}_{{{{{{{\rm{Na}}}}}}}_{2}{{{{{{\rm{S}}}}}}}_{2}{{{{{{\rm{O}}}}}}}_{3}}}{2}}{\frac{{{{{{\rm{i}}}}}}\times {{{{{\rm{t}}}}}}}{2{{{{{\rm{F}}}}}}}}$$

where *i* is the current, *t* is the time, *F* is the faraday constant (96,485 C mol^−1^), and ${{{{{{\rm{V}}}}}}}_{{{{{{{\rm{Na}}}}}}}_{2}{{{{{{\rm{S}}}}}}}_{2}{{{{{{\rm{O}}}}}}}_{3}}$ is the volume of the Na_2_S_2_O_3_ solution used.

The TOF values were calculated using the following equation:

7 $${{{{{\rm{TOF}}}}}}=\frac{{\it{J}}\times {\it{A}}}{2\times {\it{F}}\times {\it{m}}}$$

Where *J* is the obtained current density at the different overpotentials, *A* is the geometric area of the electrode, *F* is the faraday constant (96,485 C mol^−1^), and *m* is the number of moles of Ru loaded on the electrode.

For the flow cell test, Nafion 324 membrane was used to separate two compartments. 0.1 mg cm^−2^ catalysts loaded gas diffusion layer electrode was used as the anode with Pt/C doped on carbon paper as the cathode. The geometric surface area of the catalyst is 1 cm^2^. The flow rate of NaCl in the anode was controlled at 10 ml min^−1^ by using a syringe pump. During the electrochemical measurement, argon gas is continuously flowing into the anode electrolyte to bring Cl_2_-generated outside. The generated Cl_2_ product was detected through the iodometric titration method.

### In situ Raman and SR-FTIR measurements

The in situ Raman spectra were obtained through a Raman spectrometer with 532 nm excitation laser (LabRAM HR, HORIBA Jobin Yvon Inc.). A rough Au electrode with 0.5 mg cm^−2^ catalysts loading was used as the work electrode. Pt mesh and Ag/AgCl electrode were used as the counter electrode and reference electrode, respectively.

The in situ SR-FTIR was measured at beamline BL01B in National Synchrotron Laboratory. The infrared beamline is extracted through a self-made top-plate cell reflection infrared device assembled with ZnSe infrared transmission window. The end station is equipped with a Bruker FTIR spectrometer with a KBr beam splitter. Liquid nitrogen-cooled mercury cadmium telluride detector was used in this study. The sample was uniformly coated on the carbon paper as the working electrode, and the working electrode was tightly compacted by the ZnSe crystal window to reduce the loss of infrared light.

### XAS data collection and analysis

The Ru *K*-edge XAS spectra were measured at the BL 14W1 beamline at the Shanghai Synchrotron Radiation Facility (SSRF), China. Operando XAS measurements were performed by using a homemade cell. The XAS spectra were collected through the solid-state detector to obtain weak signals in the electrochemical reaction process. The catalysts were uniformly and stably distributed over carbon paper as the working electrode, and the back of the carbon paper is fixed with caption film to ensure that all electrocatalysts can react with the electrolyte.

XAS analysis was processed based on the standard procedures using the ATHENA and ARTEMIS modules implemented in the IFEFFIT software packages^51, 52^. The EXAFS signal was first collected by background subtraction and normalization, then the *k*^2^-weighted χ(k) data were Fourier-transformed to real (*R*) space using a Hanning window. The quantitative structural parameters around Ru atoms were obtained through a least-squares curve-fitting, which was carried out based on the EXAFS χ(k) data in *R*-space.

### Computational method

The first-principles calculations in the framework of DFT including structural and electronic performances were carried out based on the Cambridge Sequential Total Energy Package known as CASTEP^53^. The exchange-correlation functional under the generalized gradient approximation (GGA)^54^ with norm-conserving pseudopotentials and Perdew–Burke–Ernzerhof functional was adopted to describe the electron-electron interaction^55^. An energy cutoff of 750 eV was used and a k-point sampling set of 7 × 7 × 1 was tested to be converged. A force tolerance of 0.01 eV Å^−1^, energy tolerance of 5.0 × 10^−7^ eV per atom and maximum displacement of 5.0 × 10^−4^ Å were considered. The surfaces of RuO_x_C_4-x_C_10_ (001), RuO_x_C_4-x_C_12_ (001), and RuO_2_ (110) were built with the vacuum space along the z-direction set to be 18 Å, which is enough to avoid interaction between the two neighboring images. All atoms were relaxed for RuO_x_C_4–x_C_10_ (001) and RuO_x_C_4-x_C_12_ (001) systems, the bottom three atom layers were fixed and three top atom layers were relaxed for RuO_2_(110) systems. Then the intermediates of Cl, OH, O, and OOH groups were absorbed on the surface of RuO_x_C_4–x_C_10_ (001), RuO_x_C_4–x_C_12_ (001), and RuO_2_ (110) substrates. A moderate on-site Coulomb repulsion *U* = 4.2 eV was applied for the Ru atom.

The formation energies of RuO_x_C_4–x_ moieties in graphene were calculated according to the following equation:^56^

8 $${{\it{E}}}_{{{{{{\rm{f}}}}}}}={\it{E}}\left[{{{{{\rm{Ru}}}}}}{{{{{{\rm{O}}}}}}}_{{{{{{\rm{x}}}}}}}{{{{{{\rm{C}}}}}}}_{4-{{{{{\rm{x}}}}}}}\right]-{{{{{\rm{n}}}}}}{{{{{{\rm{\mu }}}}}}}_{{{{{{\rm{C}}}}}}}-{{{{{\rm{m}}}}}}{{{{{{\rm{\mu }}}}}}}_{{{{{{\rm{O}}}}}}}-{{\it{E}}}_{{{{{{\rm{Ru}}}}}}}$$

where E[RuO_x_C_4–x_] is the total energy of RuO_x_C_4–x_ systems; *μ*_C_ and *μ*_O_ are the chemical potentials of C and O, defined as the total energy per atom in graphene and oxygen molecules; *E*_Ru_ is the total energy of an isolated Ru atom. *n* and *m* are the numbers of C and O atoms in RuO_x_C_4-x_ systems. The energy of O_2_ gas is calculated by [2*E*(H_2_O) − 2*E*(H_2_) + 4.92] due to the negative experimental energy of the formation of two water molecules (−2${\Delta }_{{{{{{{\rm{H}}}}}}}_{2}{{{{{\rm{O}}}}}}}^{{{\exp }}}$ = 4.92 eV)^57^.

Adsorption energy Δ*E* of A group on the surface of substrates was defined as:

9 $$\Delta {\it{E}}={{\it{E}}}_{*{\it{A}}}-({{\it{E}}}_{*}+{{\it{E}}}_{{\it{A}}})$$

where *A and * denote the adsorption of A group on substrates and the bare substrates, *E*_A_ denotes the energy of A group.

Gibbs free-energy change (Δ*G*) of each chemical reaction is calculated by^58^:

10 $$\Delta {\it{G}}=\Delta {\it{E}}+\Delta {\it{ZPE}}-{\it{T}}\Delta {\it{S}}$$

where *E*, ZPE, *T*, and *S* denote the calculated total energy, zero point energy, temperature, and entropy, respectively. Here, *T* = 300 K is considered.

## Supplementary information

Supplementary Information

Peer review file

Description of Additional Supplementary Information

Supplementary Movie 1

## Data Availability

Data that support the findings of this study are from the corresponding author on reasonable request.
