# Supplementary material for: Electrosynthesis of chlorine from seawater-like solution through single-atom catalysts
Source: Nat Commun. 2023 Apr 29;14:2475. doi: 10.1038/s41467-023-38129-w (PMC10148798; doi:10.1038/s41467-023-38129-w)
Supplement: Supplementary file 1 — Supplementary Information [file 41467_2023_38129_MOESM1_ESM.pdf]

Supplementary Information

Electrosynthesis of chlorine from seawater-like solution through single-atom catalysts

Yangyang Liu,[1,2,10] Can Li,[3,10] Chunhui Tan,[2,10] Zengxia Pei,[2] Tao Yang,[4] Shuzhen Zhang,[2] Qianwei Huang,[5] Yihan Wang,[1,2] Zheng Zhou,[2] Xiaozhou Liao,[5] Juncai Dong,[6] Hao Tan,[7]* Wensheng Yan,[7] Huajie Yin,[8] Zhao-Qing Liu,[9] Jun Huang,[2]* Shenlong Zhao[1,2]*

[1]CAS Key Laboratory of Nanosystem and Hierarchical Fabrication, CAS Center for Excellence in Nanoscience, National Center for Nanoscience and Technology, Beijing 100190, China.

[2]School of Chemical and Biomolecular Engineering, The University of Sydney, Sydney 2006, Australia.

[3]Key Laboratory of Rare Earth Optoelectronic Materials and Devices of Zhejiang Province, College of Optical and Electronic Technology, China Jiliang University, Hangzhou 310018, China.

[4]Department of Mechanical Engineering, University of Aveiro, Aveiro 3810-93, Portugal.

[5]School of Aerospace, Mechanical and Mechatronic Engineering, The University of Sydney, Sydney 2006, Australia.

[6]Beijing Synchrotron Radiation Facility, Institute of High Energy Physics, Chinese Academy of Sciences, Beijing 100049, China.

[7]National Synchrotron Radiation Laboratory, University of Science and Technology of China, Hefei 230029, China.

[8]Institute of Solid-State Physics, Chinese Academy of Sciences, Hefei 230031, China

[9]School of Chemistry and Chemical Engineering/Guangzhou Key Laboratory for Clean Energy and Materials/Key Laboratory for Water Quality and Conservation of the Pearl River Delta, Ministry of Education, Guangzhou University, Guangzhou 510006, China

[10]These authors contributed equally to this manuscript.

Supplementary Figures & Tables

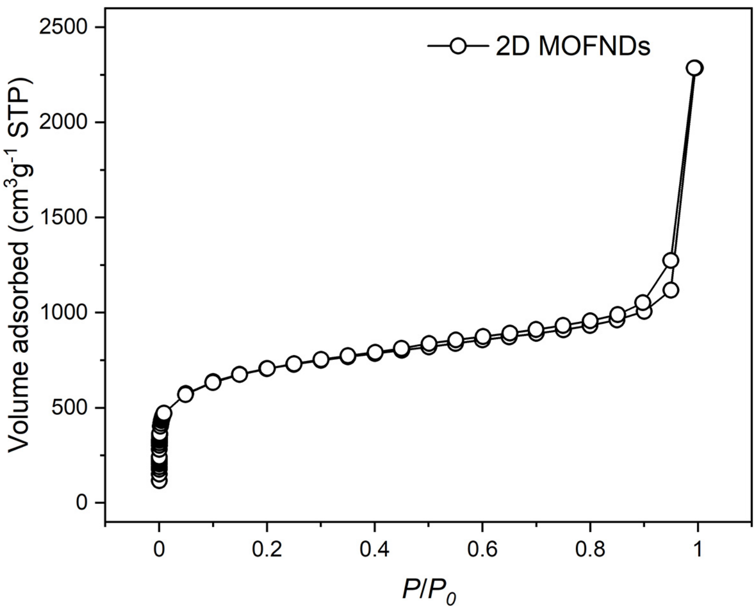

Supplementary Figure 1 | N_2 adsorption-desorption isotherm of 2D MOFNDs.

STP, standard temperature and pressure; *P* and P_0, pressure under the testing condition and the saturated vapour pressure, respectively. The result shows that the BET surface area of the 2D MOFNDs is ~1807.2 m^2g^{-1}, demonstrating this porous carbon matrix has a large surface area.

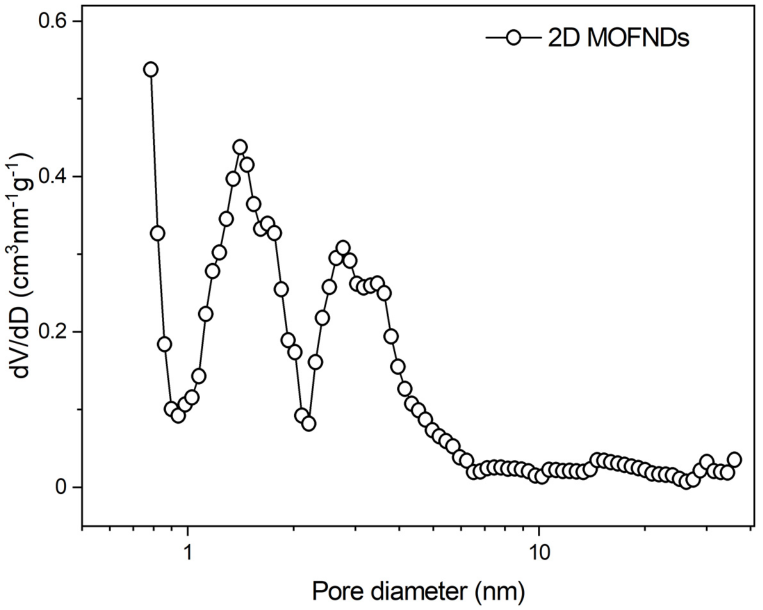

Supplementary Figure 2 | Pore size distribution for the 2D MOFNDs.

The 2D MOFNDs exhibit abundant micropore structures with pore size about 0.75 nm, which could be attributed to the carbonization of Zn-BDC and evaporation of metallic zinc.

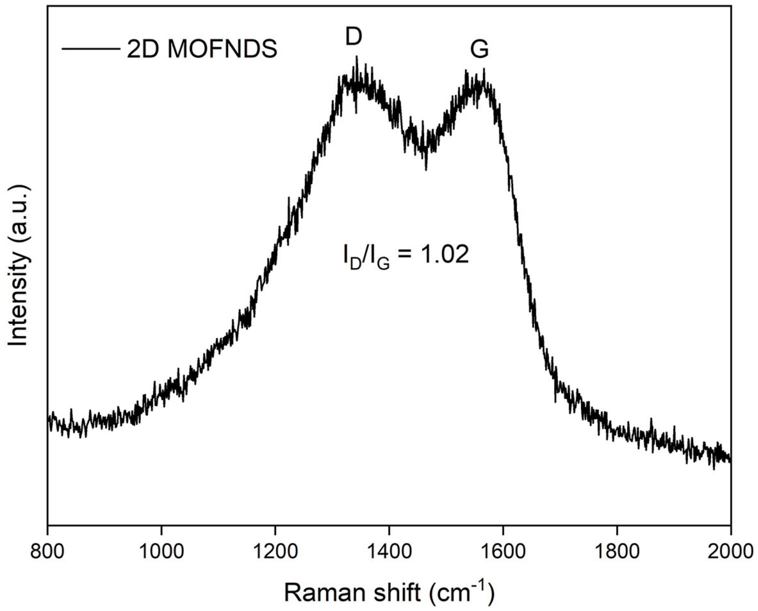

Supplementary Figure 3 | Raman spectra of 2D MOFNDs.

Only two characteristic peaks of carbon at 1350 cm^{-1} (D band, disordered/defective carbon) and 1592 cm^{-1} (G bane, graphitic carbon) were detected. The intensity ratios of the D-band to G-band (I_D/I_G) were calculated to be 1.02, suggesting the high oxygen defects enriched property.

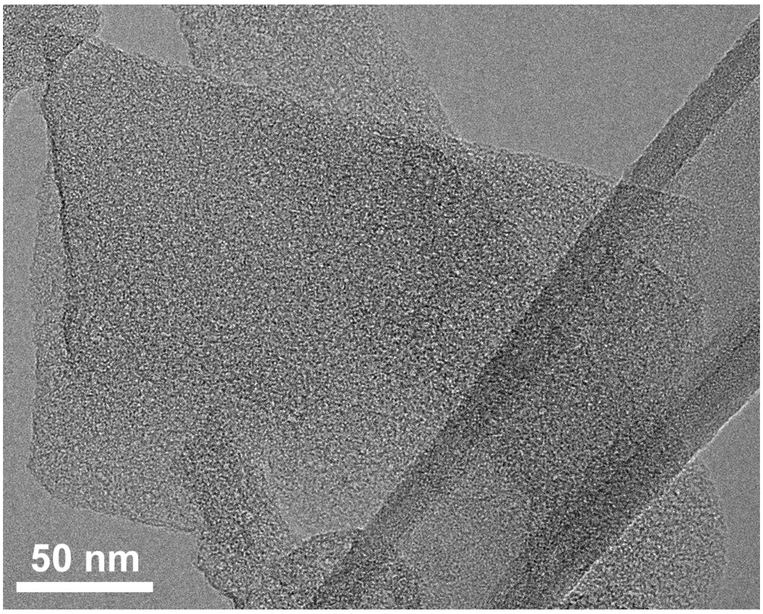

Supplementary Figure 4 | TEM images of 2D MOFNDs.

The 2D morphology of 2D Zn-BDC derivatives can be discerned in 2D MOFNDs by TEM survey.

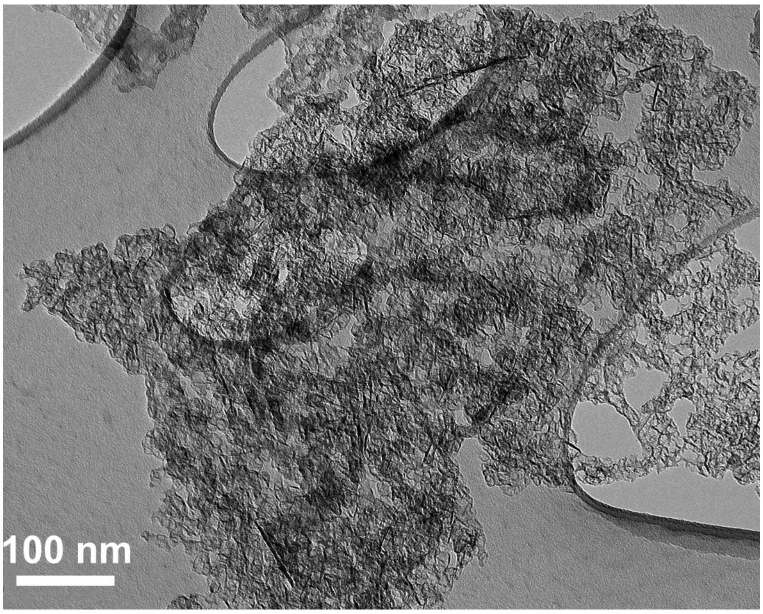

Supplementary Figure 5 | HRTEM images of the as prepared Ru-O_4 SAM.

Supplementary Fig. 5 shows that the as-prepared Ru-O_4 SAM exhibits similar 2D morphology of 2D MOFNDs. No obvious Ru nanoparticle or cluster were discerned.

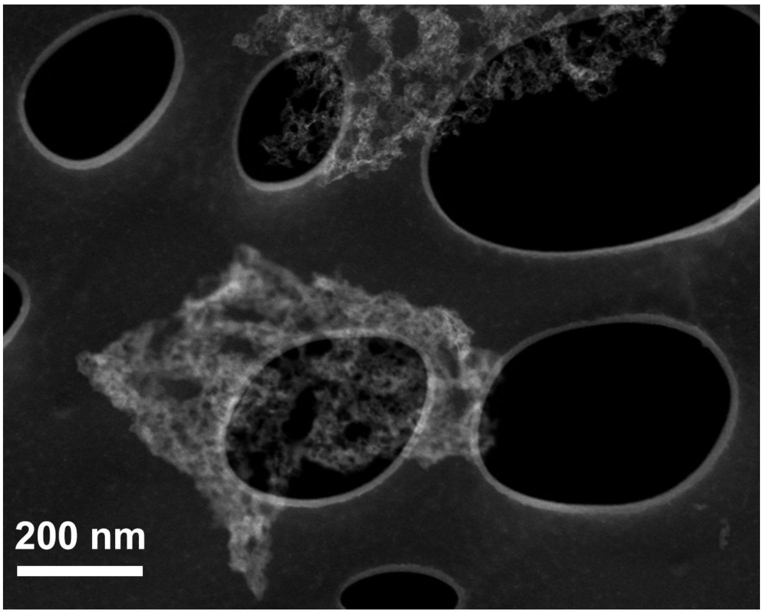

Supplementary Figure 6 | HAADF-STEM image of Ru-O_4 SAM.

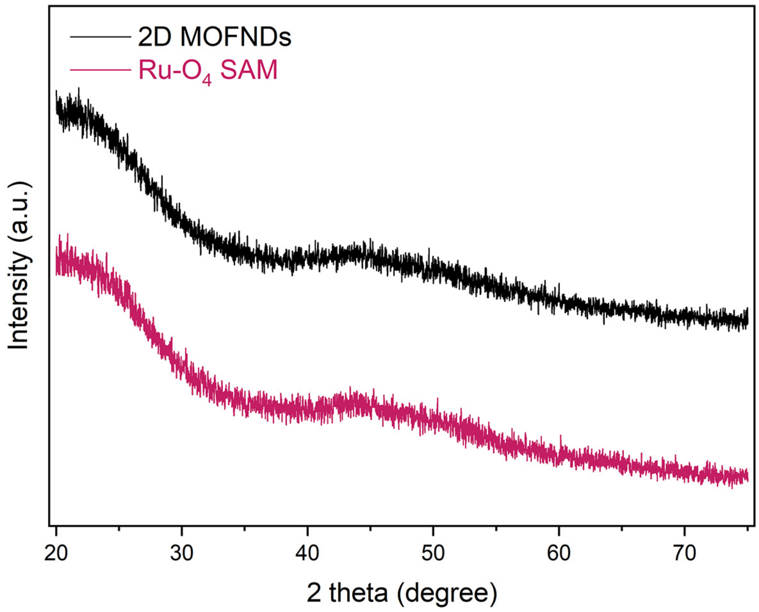

Supplementary Figure 7 | XRD for Ru-O_4 SAM and 2D MOFNDs.

Both Ru-O_4 SAM and 2D MOFNDs exhibit two peaks at 25° and 44°, which are assigned to the (002) and (101) facets of graphite carbon[1]. No peaks related to Ru crystals is observed.

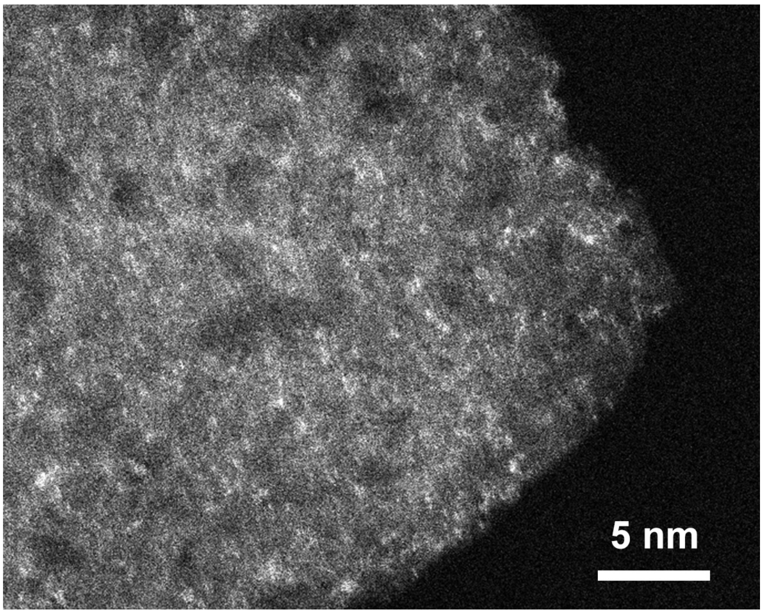

Supplementary Figure 8 | AC HAADF-STEM images of Ru-O_4 SAM.

Supplementary Fig. 8 demonstrates the Ru atoms are individually dispersed in the carbon substrate without Ru nanoparticles or cluster.

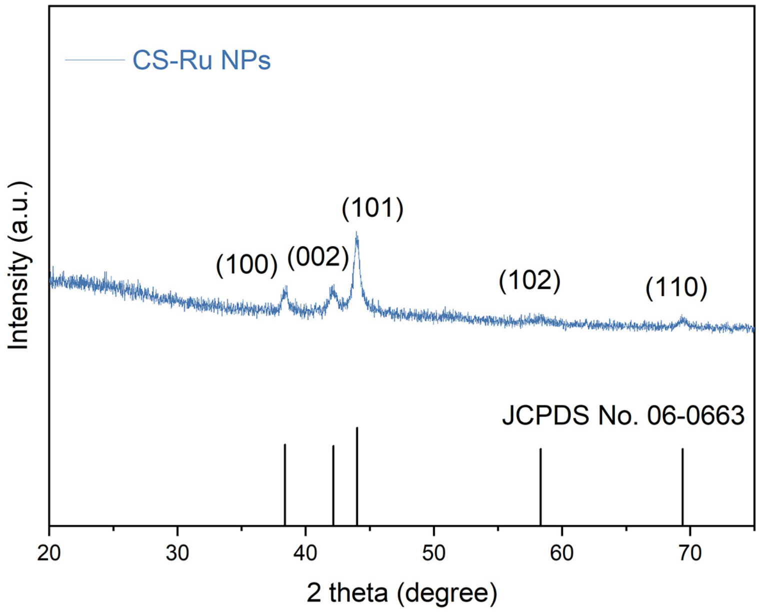

Supplementary Figure 9 | XRD for CS-Ru NPs.

The XRD pattern of CS-Ru NPs exhibits five diffraction peaks at 38°, 42°, 44°, 58°, and 68°, which are well attributed to the (100), (002), (101), (102), and (110) facet of metal Ruthenium (JCPDS No, 06-0663), respectively.

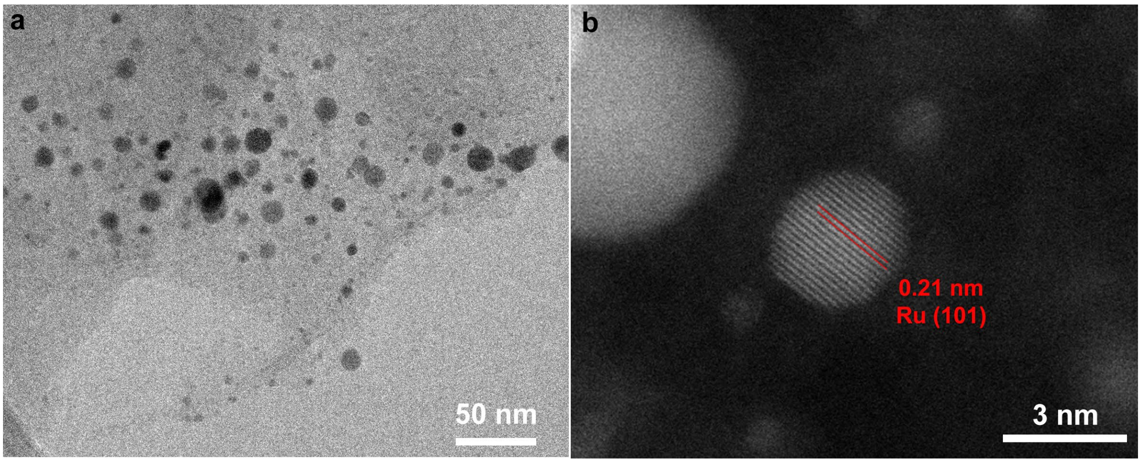

Supplementary Figure 10 | TEM studies of CS-Ru NPs. a, TEM images of CS-Ru NPs. **b,** HAADF-STEM images of CS-Ru NPs.

Supplementary Fig. 10b exhibited well-defined lattice fringes, with interplanar spacing of 0.21 nm that are characteristic of the (101) planes of *hcp* Ru (JCPDS No, 06-0663). The result was consistent to the result from XRD measurement (Supplementary Fig. 9).

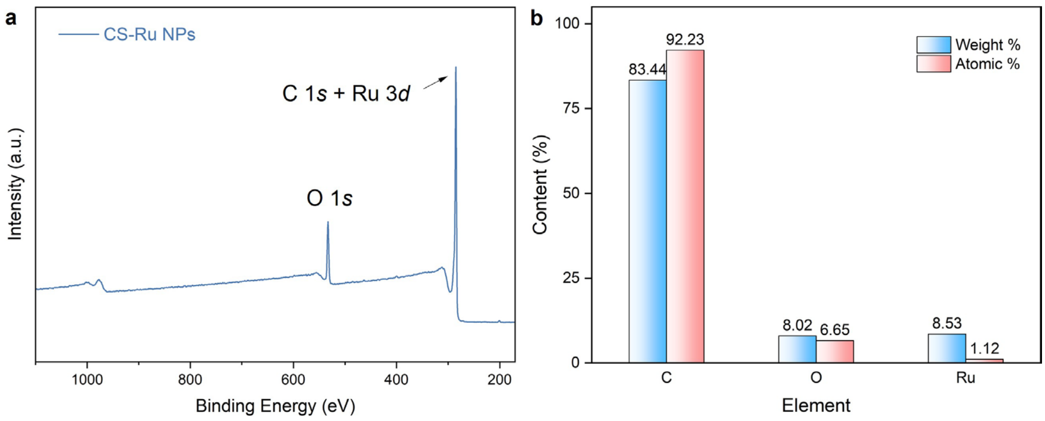

Supplementary Figure 11 | XPS spectrum of CS-Ru NPs.

The XPS survey spectra confirmed the obtained CS-Ru NPs are composed of C, O and Ru species without other impurities. The corresponding contents of C, O and Ru species are summarized in Supplementary Fig. 11b.

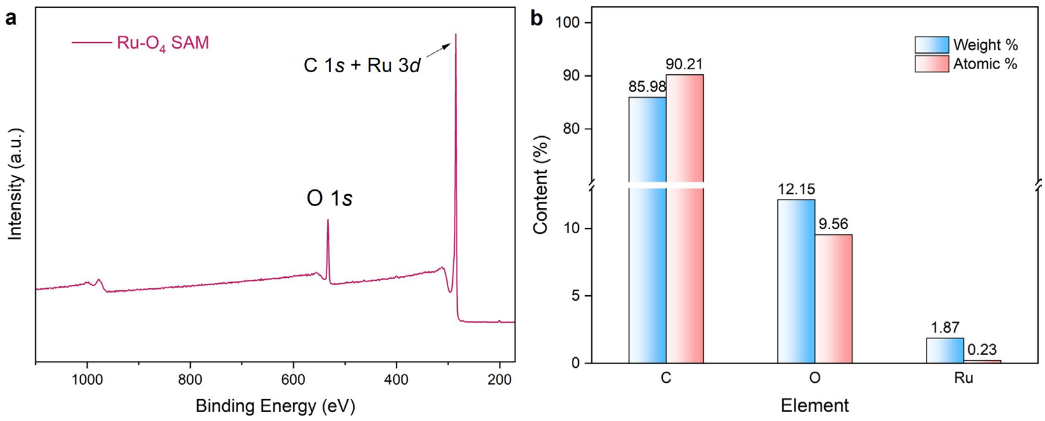

Supplementary Figure 12 | XPS spectrum of Ru-O₄ SAM.

The XPS survey spectra confirmed the obtained Ru-O_4 SAM are composed of C, O and Ru species without other impurities. The corresponding contents of C, O and Ru species were summarized in the Supplementary Fig. 12b.

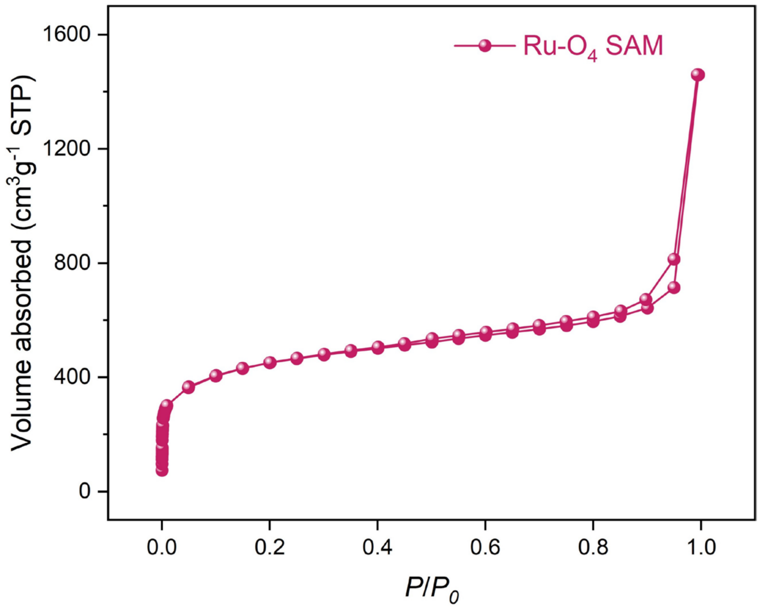

Supplementary Figure 13 | N_2 adsorption-desorption isotherm isotherm of Ru-O_4 SAM.

STP, standard temperature and pressure; *P* and P_0, pressure under the testing condition and the saturated vapour pressure, respectively. The result shows that the BET surface area of the Ru-O_4 SAM is ~1320 m^2g^{-1}, demonstrating its large surface area.

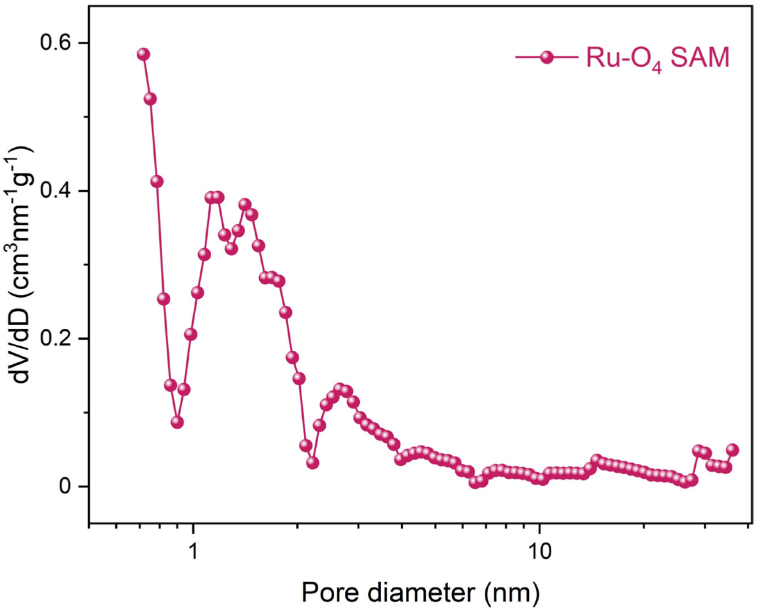

Supplementary Figure 14 | Pore size distribution for the Ru-O_4 SAM.

The Ru-O_4 SAM exhibits abundant micropore structures with pore size of ~ 0.75 nm, which could be attributed to the carbonization of Zn-BDC and evaporation of metallic zinc.

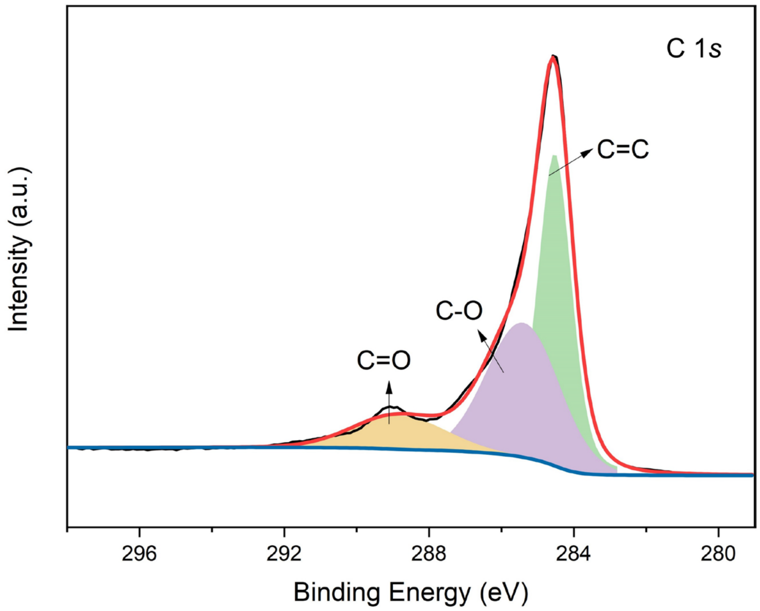

Supplementary Figure 15 | The XPS detailed scan of the carbon region of the Ru-O_4 SAM.

The C 1*s* spectrum can be deconvoluted to three characteristic peaks at 284.8, 285.8, and 289.7 eV, respectively. These peaks can be attribute to C=C, C-O, and C=O[2]. No peaks located at 296.8 eV relating to Ru-C bonds is observed[3].

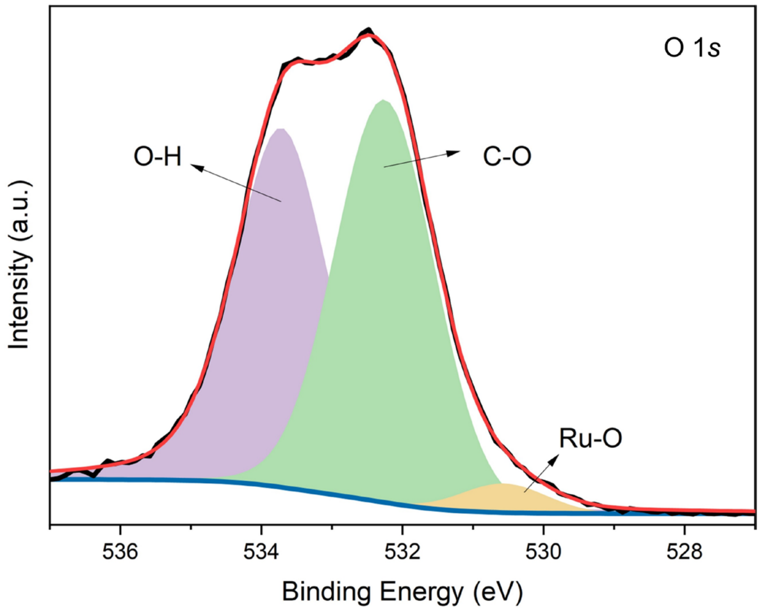

Supplementary Figure 16 | The XPS detailed scan of the oxygen region of the Ru-O_4 SAM.

The O 1*s* spectrum can be deconvoluted to three characteristic peaks at 530.5, 532.3, and 533.8 eV, respectively. Besides the peaks corresponding to C-O (epoxy and hydroxyl) and O-H (carboxyl and phenolic oxygen), and additional peak at 530.5 eV that is attributed to Ru-O bonds[4].

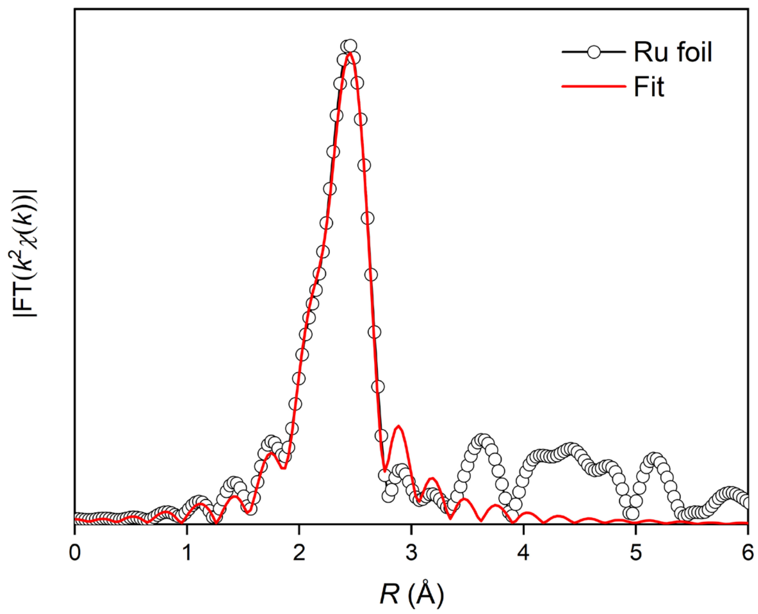

Supplementary Figure 17 | Ru *K*-edge EXAFS spectra of Ru foil.

Ru *K*-edge EXAFS spectra and the Fourier-transformed magnitudes for Ru foils. The calculated spectra are matched well with the experimental results. The best fitting parameters are shown in Supplementary Table 1.

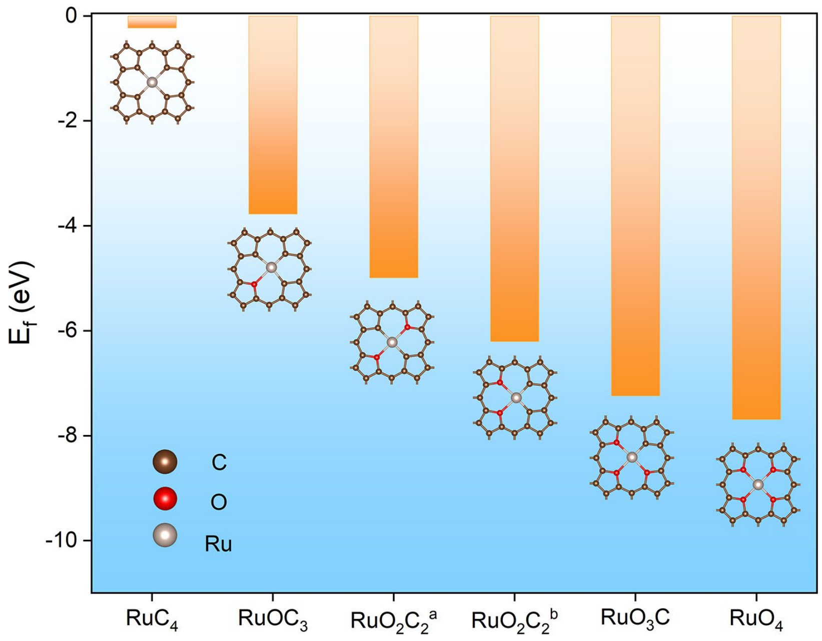

Supplementary Figure 18 | DFT calculated formation energies of various RuO_xC_y. (X+Y=4, X=1, 2, 3, and 4; a represents two O atoms are opposite. B represents two oxygen atoms are adjacent).

The results show the RuO_4 (-7.7 eV) is much more energetic favorable than RuO_3C (-7.2 eV), $RuO_2C_2^a$ (-5.0 eV), $RuO_2C_2^b$ (-6.2 eV), $RuOC_3$ (-3.8 eV) and RuC_4 (-0.23 eV), suggesting that the Ru-O_4 moiety is the most stable structure.

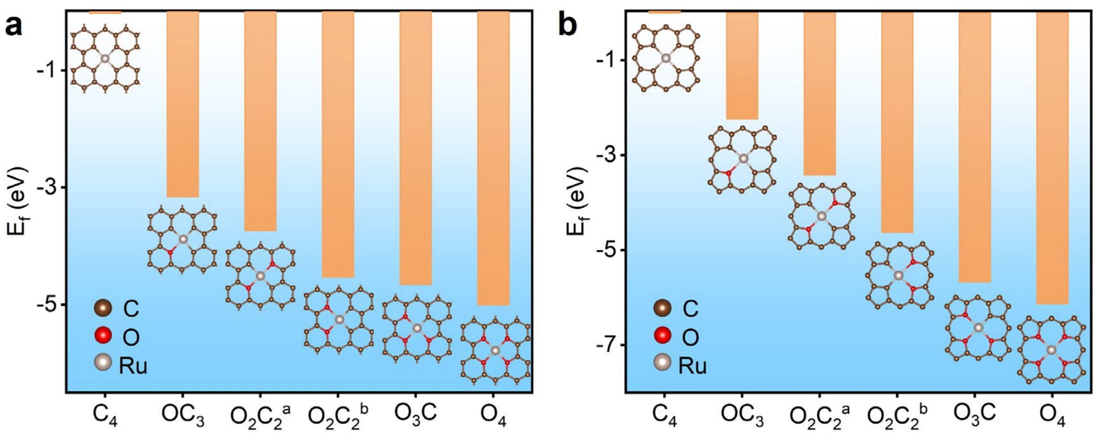

Supplementary Figure 19 | DFT calculated formation energies of various RuO_xC_y under CER condition (X+Y=4, X=1, 2, 3, and 4; a represents two O atoms are opposite. B represents two oxygen atoms are adjacent).

As the Supplementary Fig. 19 shows, both Ru-O_4-C_{10}/C_{12} are more energetically favourable (-5 eV and -6.1 eV) than other structures RuO_3C-C_{10}/C_{12} (-4.7 eV/-5.7 eV), $RuO_2C_2^a$-C_{10}/C_{12} (-3.7 eV/-3.4 eV), $RuO_2C_2^b$-C_{10}/C_{12} (-4.5 eV/-4.6 eV), $RuOC_3$-C_{10}/C_{12} (-3.2 eV/-2.2 eV) and RuC_4-C_{10}/C_{12} (-0.02 eV/1.3 eV), suggesting that Ru-O_4 moiety is still the most thermodynamic structure under CER condition

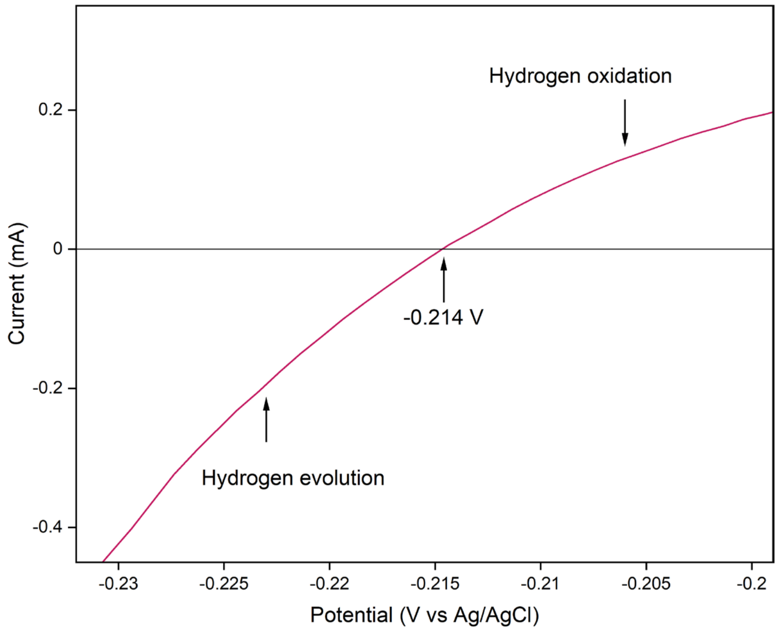

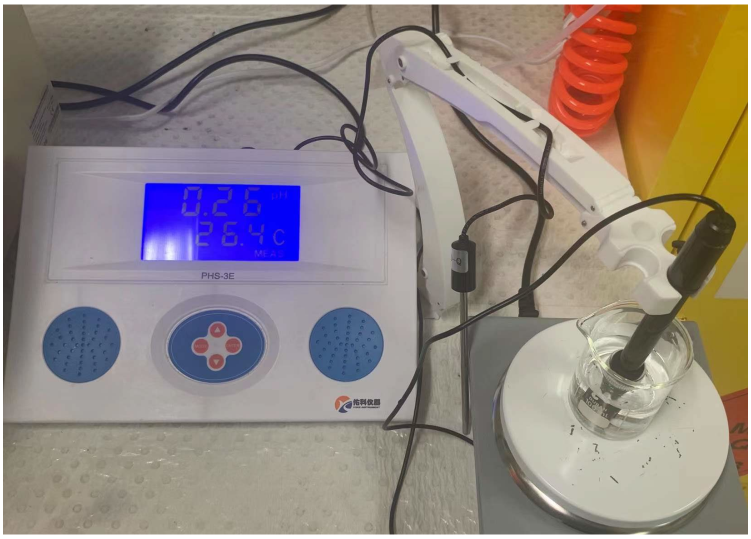

Supplementary Figure 20 | Reference electrode calibration.

The calibration of used Ag/AgCl electrode was performed in a three-electrode system with 0.5 M H_2SO_4 solution with a scan rate of 1 mV s^{-1}. Pt foil, Pt mesh, and Ag/AgCl served as the working, counter, and reference electrode, respectively. As shown in supplementary Fig. 19, the zero current point is at around -0.214 V in 0.5 M H_2SO_4 solution, the pH of 0.5 M H_2SO_4 is measured as 0.26. Hence, the potential for the used Ag/AgCl electrode is around 0.199 V, which is almost the same as the standard potential (0.197 V) of Ag/AgCl/Saturated KCl electrode.

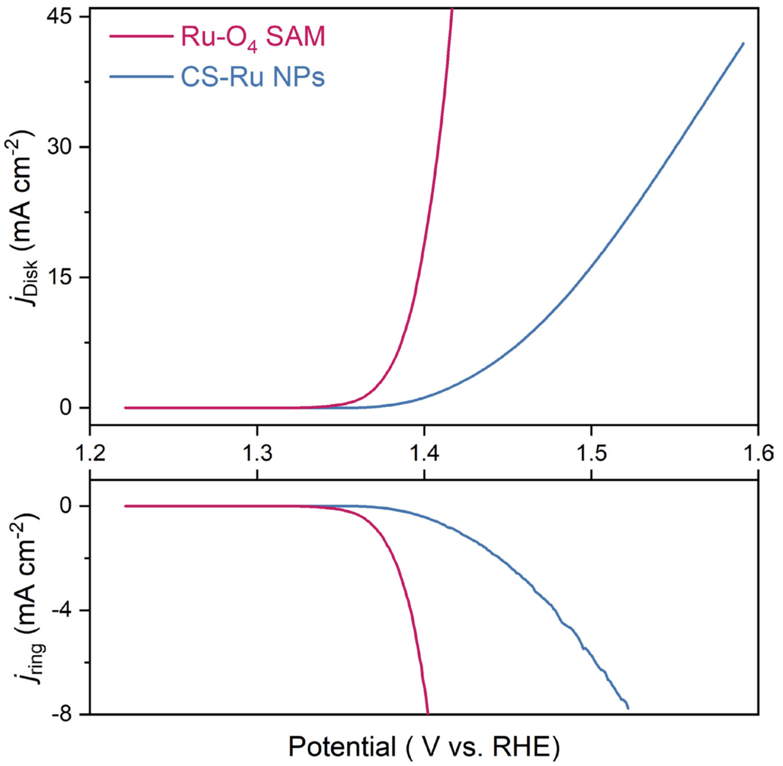

Supplementary Figure 21 | RRDE measurement of CER performance on Ru-O$_4$ SAM and CS-Ru NPs.

The polarization curves of Ru-O$_4$ SAM and CS-Ru NPs were measured in 1M NaCl solution with pH of 1 at a scan rate of 5 mV s^{-1} and an electrode rotation speed of 1600 rpm. The ring potential was fixed at 0.95 V.

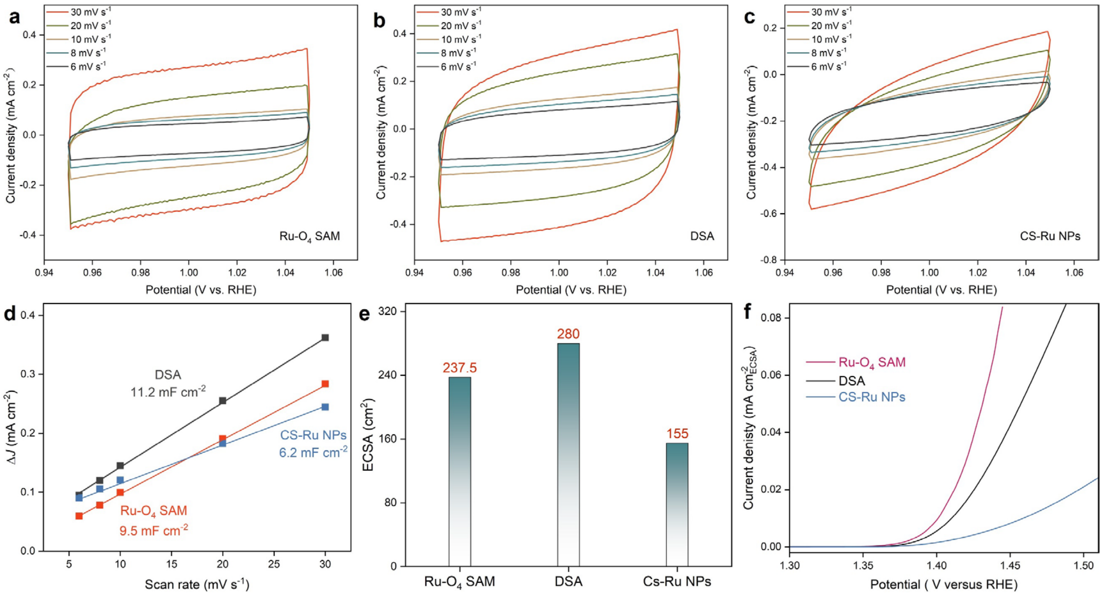

Supplementary Figure 22 | ECSA measurement of CER performance on Ru-O_4 SAM, CS-Ru NPs and DSA.

a-c, Cyclic voltammetry (CV) graphs for (**a**) Ru-O_4 SAM, (**b**) DSA, and (**c**) CS-Ru NPs catalysts at different scan rats from 6 to 30 mV s^{-1}, respectively. **d,** The plot of current density at 1.0 V vs. RHE against the scan rate. The slope is the double layer capacitance (C_{dl}). **e,** ECSA of Ru-O_4 SAM, DSA, and CS-Ru NPs catalysts. **f,** ECSA-normalized LSVs of Ru-O_4 SAM, DSA, and CS-Ru NPs catalysts.

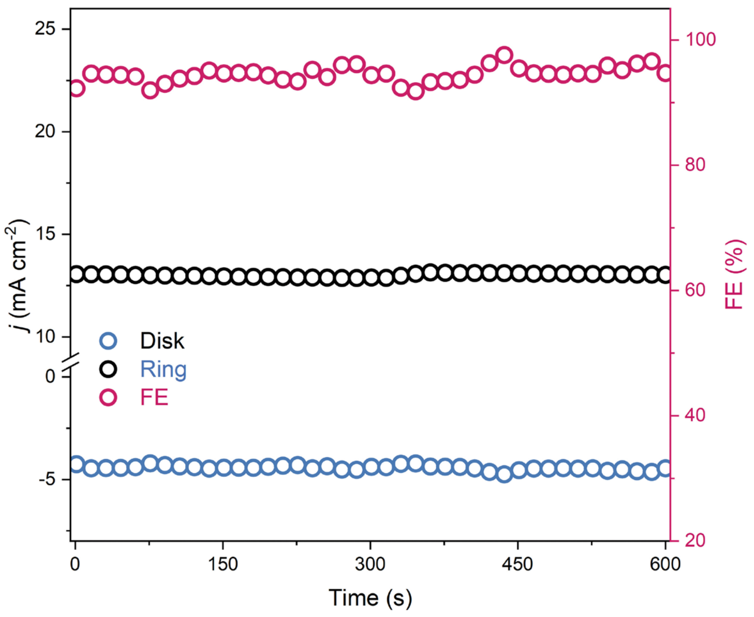

Supplementary Figure 23 | Cl_2 selectivity testing of CS-Ru NPs using RRDE methods.

Cl_2 selectivity testing of CS-Ru NPs using RRDE method was carried out in an Ar-saturated 1 M NaCl solution with pH of 1. When a constant potential is applied to the disk electrode for Cl_2 generation for 600 s, a ring current caused by Cl_2 reduction is detected immediately. The selectivity of CS-Ru NPs for CER is around 95%.

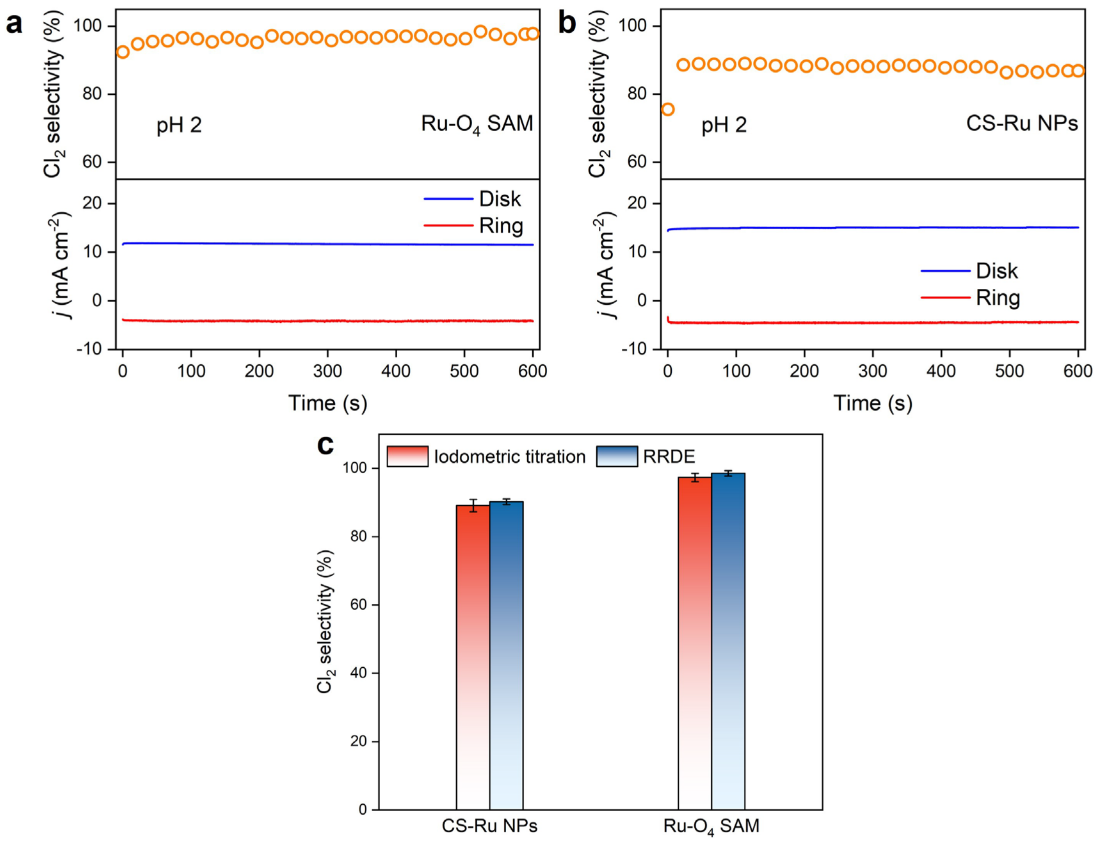

Supplementary Figure 24 | Cl_2 selectivity testing of Ru-O4 SAM, CS-Ru NPs and DSA at pH=2.

a,b, Cl_2 selectivity testing of (**a**) Ru-O_4 SAM and (**b**) CS-Ru NPs using RRDE technique in Ar-saturated 1 M NaCl solution with pH of 2. When a constant potential is applied to the disk electrode for Cl_2 generation for 600 s, a ring current caused by Cl_2 reduction is detected immediately. **c**. The Cl_2 selectivity test via iodometric titration method.

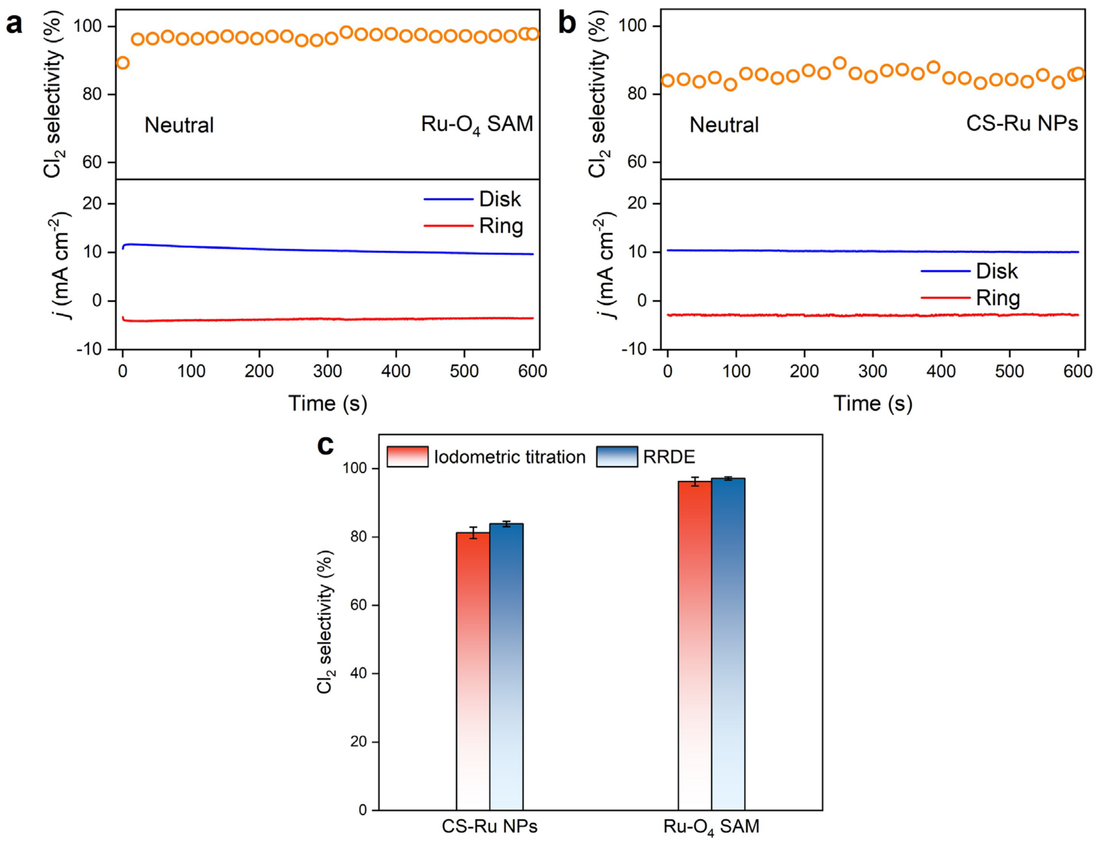

Supplementary Figure 25 | Cl_2 selectivity testing of Ru-O4 SAM, CS-Ru NPs and DSA at neutral pH.

a,b, Cl_2 selectivity testing of (**a**) Ru-O_4 SAM and (**b**) CS-Ru NPs using RRDE technique in Ar-saturated 1 M NaCl solution without pH adjustment. When a constant potential is applied to the disk electrode for Cl_2 generation for 600 s, a ring current caused by Cl_2 reduction is detected immediately. **c**. The Cl_2 selectivity test via iodometric titration method.

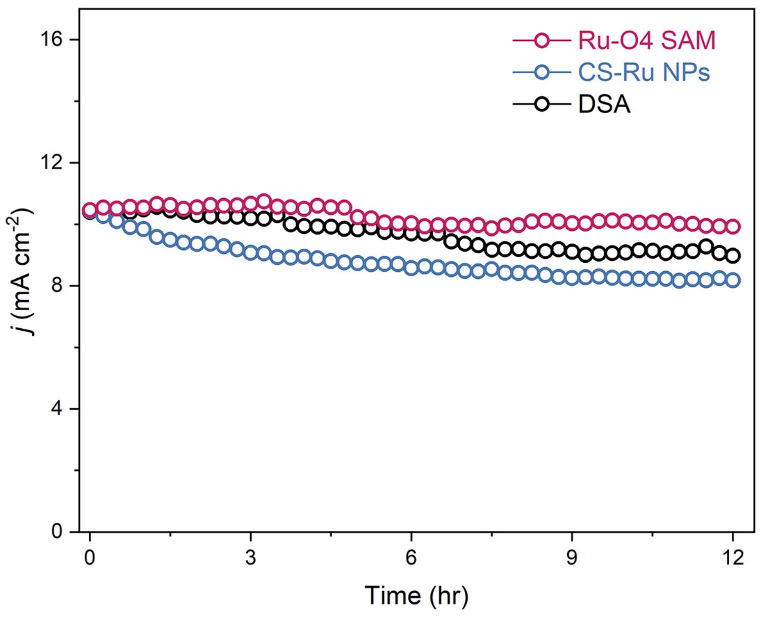

Supplementary Figure 26 | Stability measurement of Ru-O_4 SAM, CS-Ru NPs, and DSA.

The stability of prepared catalysts and DSA was examined by chronoamperometry at an initial current density of 10 mA cm^{-2}. After 12 hours operation, the current density (10 mA cm^{-2}) at Ru-O_4 SAM electrode retains around 95%, which is better than those of CS-Ru NPs (80%) and DSA (86%).

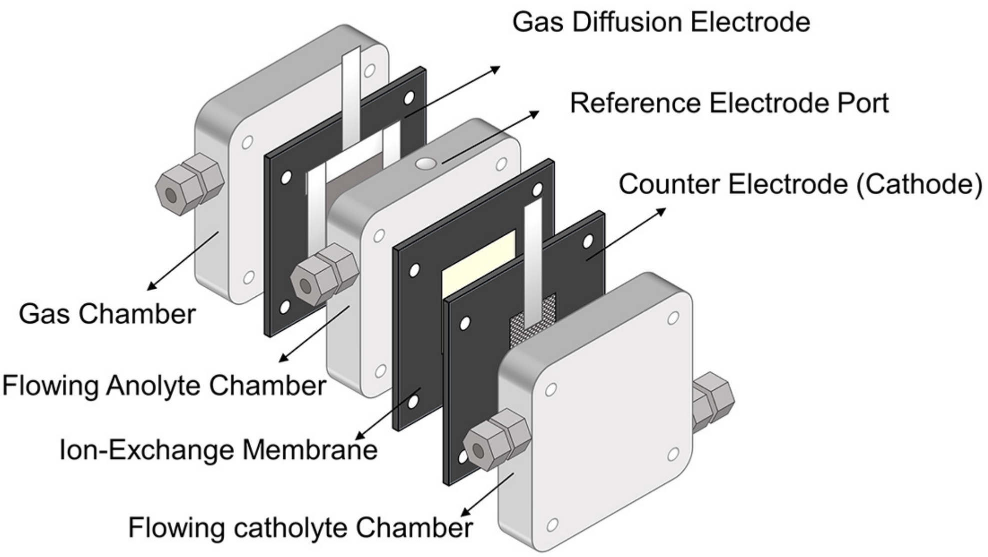

Supplementary Figure 27 | Isometric view of the exploded three-chamber cell showing individual components.

The flow cell system is equipped with Ru-O_4 doped gas diffusion electrode as the working electrode, Ag/AgCl as reference electrode, and Pt mesh as counter electrode. Pure Ar is continuingly purged into the cell through the gas chamber as the carrying gas. Produced Cl_2 could be purged out the cell and collected for quantitative analysis. Also, the anolyte is collected to quantify partially dissolved Cl_2 gas.

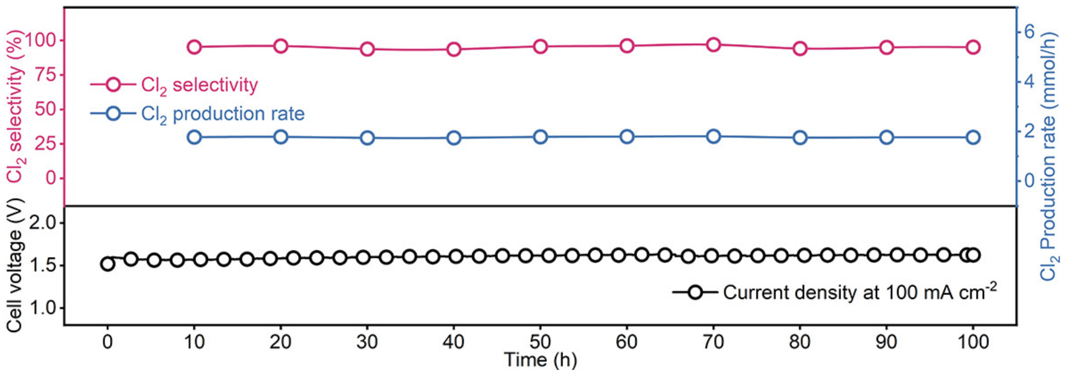

Supplementary Figure 28 | Stability test of Ru-O_4 SAM measured in 1 M NaCl using a home-made flow cell system. Current density of the flow cell is maintained at 100 mA cm^{-2}.

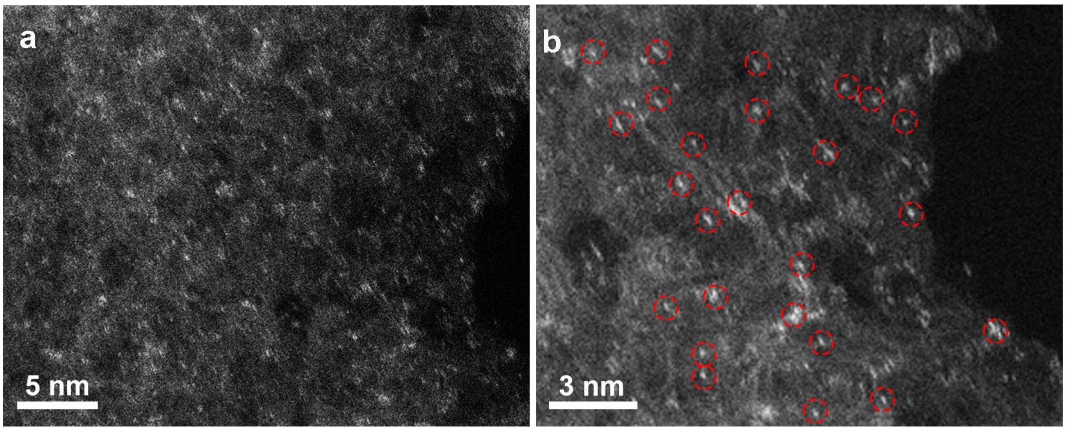

Supplementary Figure 29 | AC HAADF-STEM image of Ru-O_4 SAM after long stability test.

The monodispersion of Ru can be directly observed by the AC HAADF-STEM. The Ru atoms are confirmed by isolated bright dots in the high-magnification HAADF-STEM image. The morphology structure of Ru-O_4 SAM can be completely maintained after the long-term test.

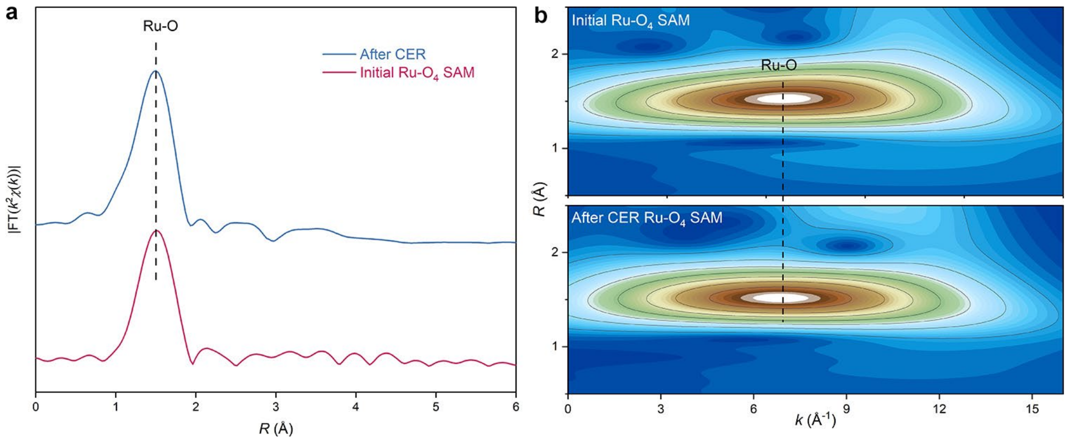

Supplementary Figure 30 | Atomic environment of Ru-O4 SAM after CER test. a, k^2-weight FT-EXAFS curves of Ru-O4 SAM before and after long-term test at Ru *K*-edge. **b,** WT-EXAFS plots of Ru-O4 SAM before and after long-term test.

The result shows that the atomic structure of Ru-O4 SAM is completely maintained after the long-term test. As shown in Supplementary Fig. 30a, Ru-O4 SAM exhibits only one dominant peak at 1.5 Å caused by the nearest shell coordination of Ru-O bonding. Notably, there is an obvious peak originated from Ru-Ru coordination at 2.3 Å for Ru foil while no related signal is observed in the FT-EXAFS spectrum, indicating that Ru atoms are still anchored on surface of 2D MOFNDs in isolation after CER test, in agreement with the AC HAADF-STEM image in Supplementary Fig 29. For the pronounced intensity corresponding to the EXAFS-FT peak at 1.5 Å in Supplementary Fig. 30b, a contour intensity maximum originated from Ru-O scattering is observed at 7.0 $Å^{-1}$ in ET-EXAFT spectra of initial and after CER test Ru-O4 SAM.

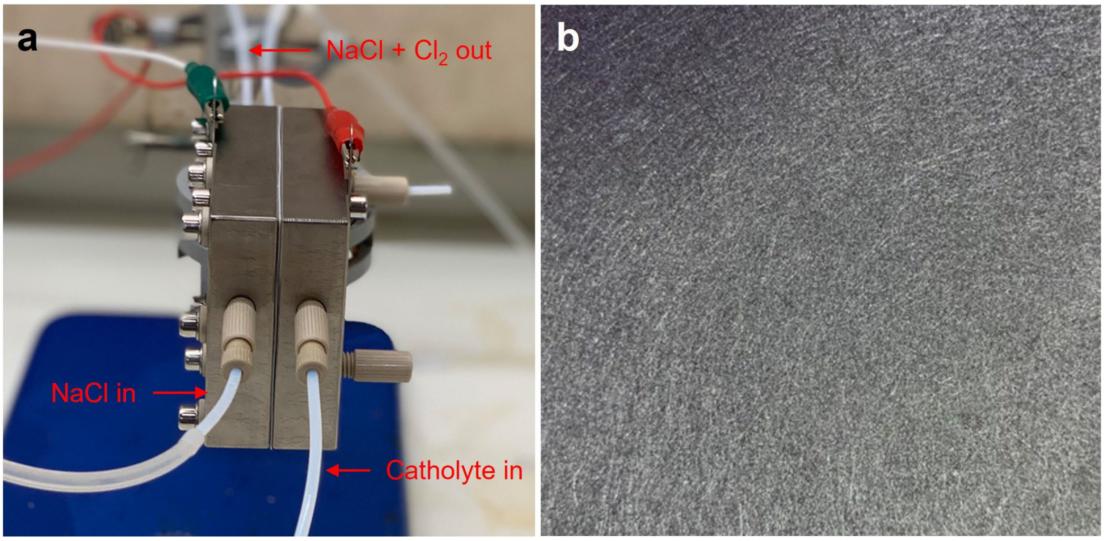

Supplementary Figure 31 | Photographs of electrolyser for large-scale Cl_2 production.

a, Photograph of the electrolyser for large-scale Cl_2 production. **b,** Photograph of the purchased high porosity titanium fibre felts as the anode substrate.

A homemade two-electrode electrolyser was assembled. Ru-O_4 SAM was drop-casted on a porous titanium fibre felts (Fuel Cell Store, 2 × 2cm) with a mass loading of ~5 mg cm-2 as the anode, and commercial Pt/C (Fuel Cell Store) coated on gas diffusion layer with a mass loading of 0.5 mg cm-2 was chosen as the cathode. The anode and cathode compartments are fully separated by a fabric-reinforced perfluorosulfonic acid membrane (GI-N417, Fuel Cell Store).

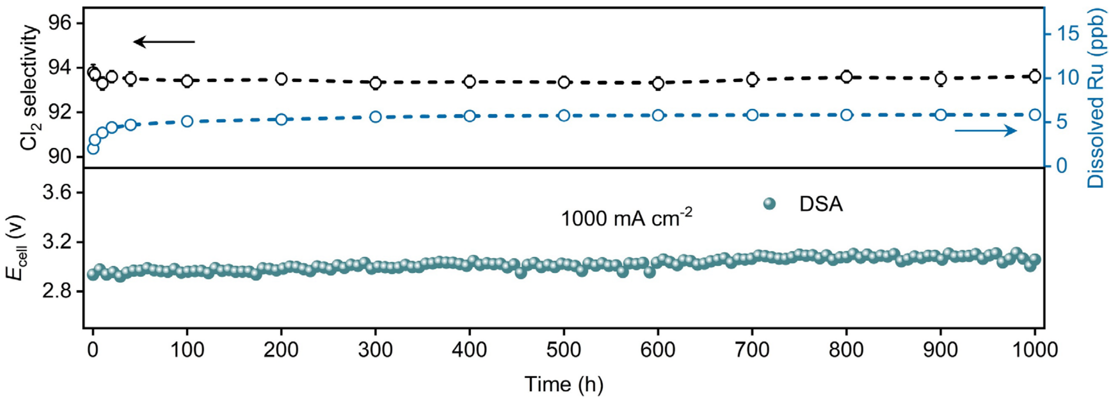

Supplementary Figure 32 | Stability test of DSA measured in 1 M NaCl using a home-made flow cell system. Current density of the flow cell is maintained at 1000 mA cm^{-2}.

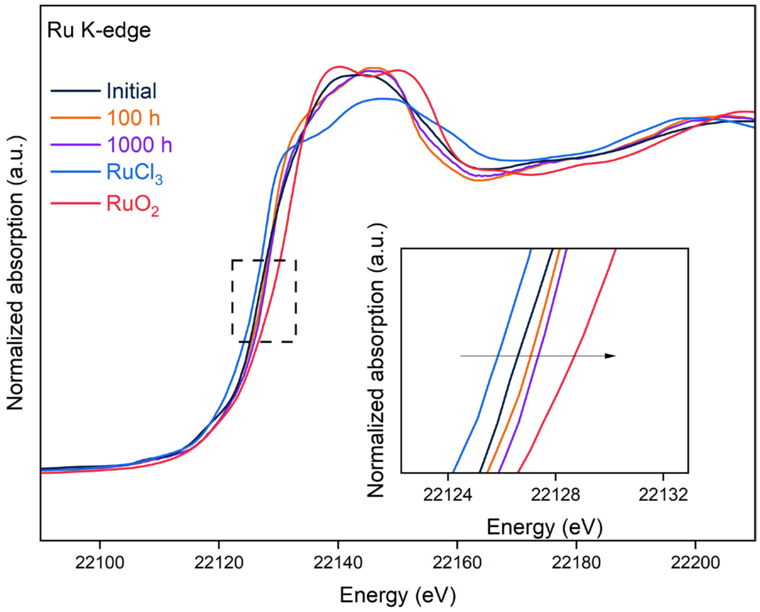

Supplementary Figure 33 | Ru *K*-edge XAS characterization of Ru-O_4 SAM after long-term operation.

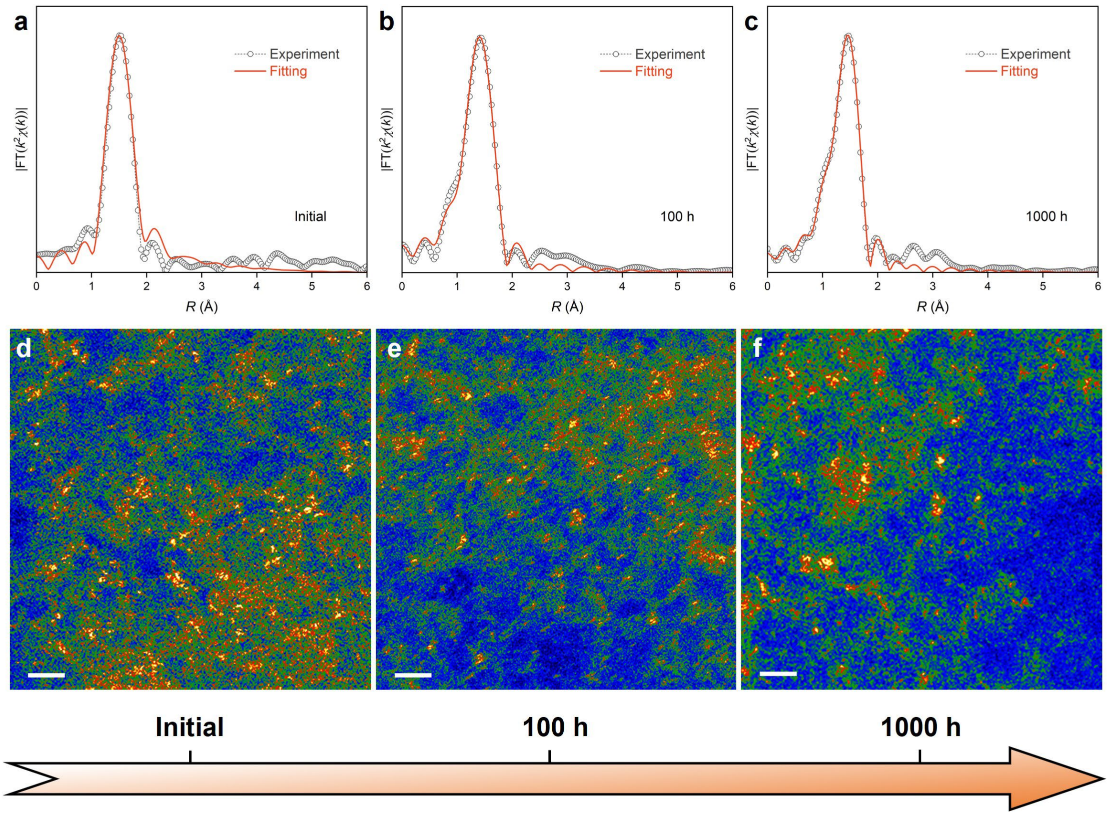

Supplementary Figure 34 | Long term characterizations for Ru-O_4 SAM.

a-c, k^2-weight FT-EXAFS fitting curves of Ru-O_4 SAM at Ru *K*-edge. **d-f**, AC-HAADF-STEM images of Ru-O_4 SAM, scale bar: 1nm.

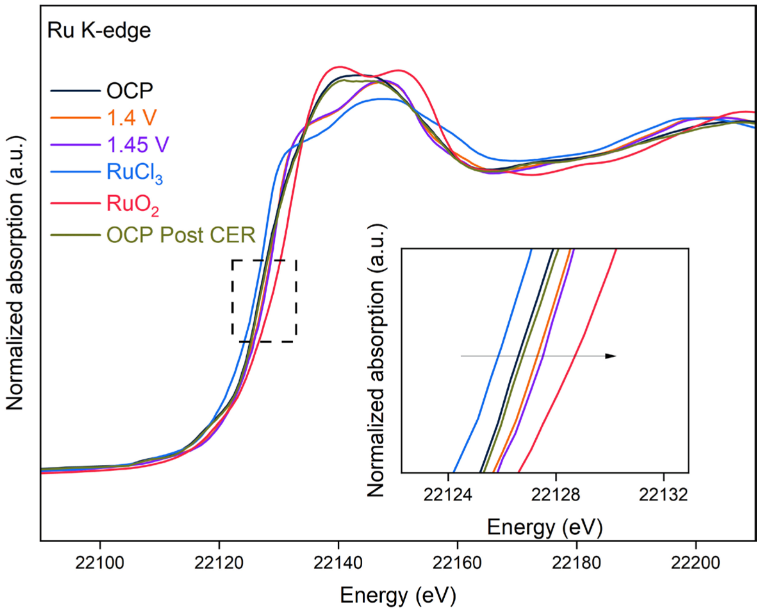

Supplementary Figure 35 | Operando Ru XANE spectra of Ru-O$_4$ SAM.

The Ru *K*-edge position in the XANES spectra slightly shifts towards higher energy when applied the reaction potential, suggesting the higher oxidation state of the Ru atom during the CER reaction. Interestingly, the oxidation state of Ru kept lower than that of RuO_2 (+4) during the whole CER process, which indicates avoiding the formation of unstable high valence species.

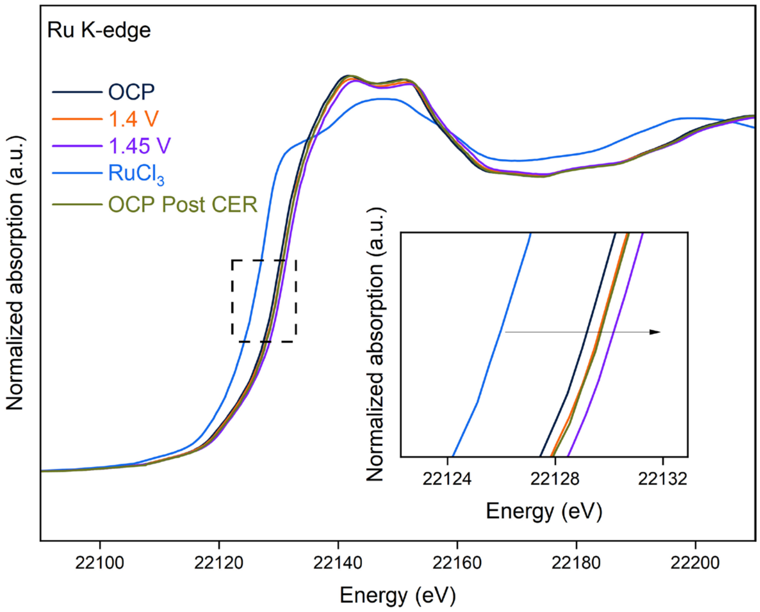

Supplementary Figure 36 | Operando Ru *K*-edge XAS characterization of RuO_2.

Supplementary Fig. 36 shows the Normalized XANES curves of RuO_2 and $RuCl_3$ at Ru *K*-edge from OCP condition to 1.45 V during CER process. Ru *K*-edge position in the XANES spectra shifts towards higher energy, suggesting the higher oxidation state of Ru. The formation of high oxidation state of Ru may cause the dissolve of RuO_2, which is consistent with our operando Raman measurement (RuO_2 lost its original surface structure)[5,6].

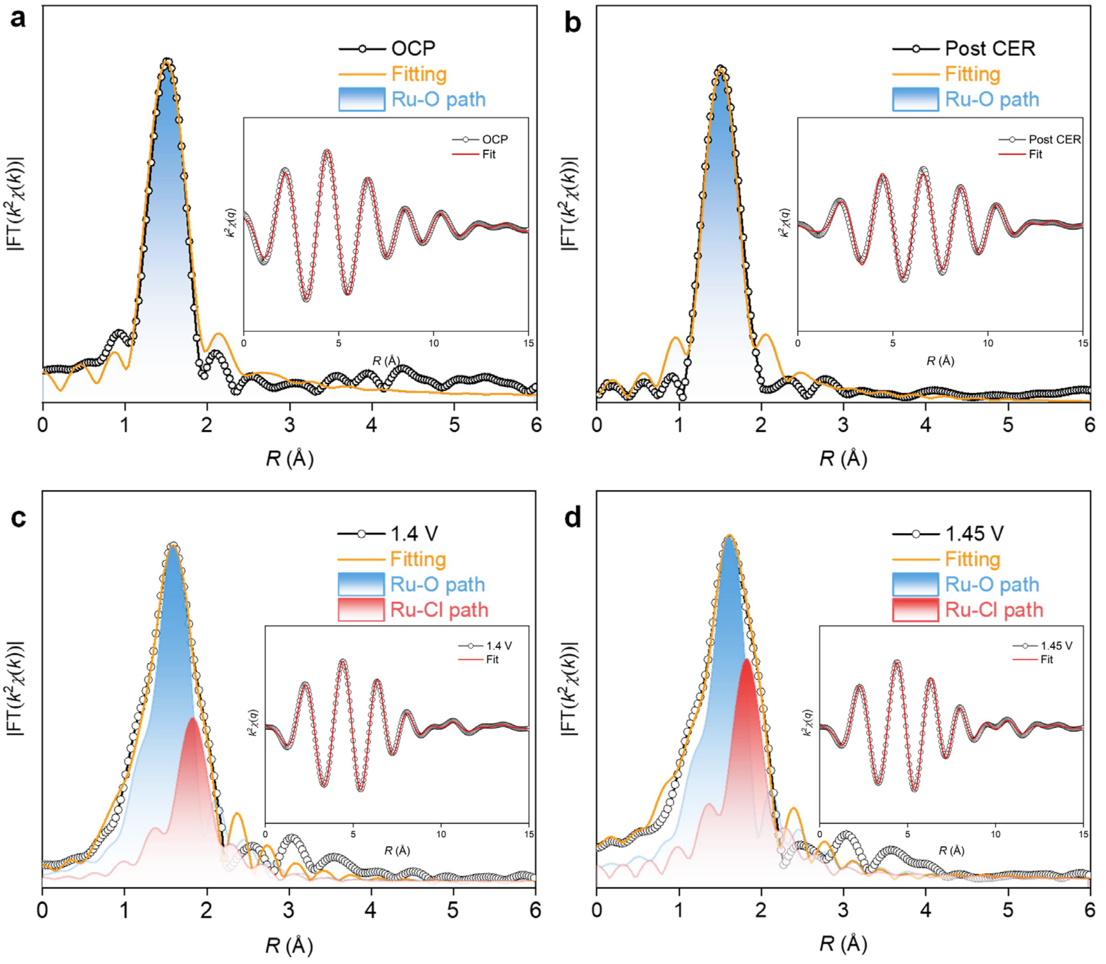

Supplementary Figure 37 | Operando Ru *K*-edge EXAFS spectra of Ru-O_4 SAM.

Operando k^2-weight Ru *K*-edge EXAFS spectra and the Fourier-transformed magnitudes for Ru-O_4 SAM are obtained from different applied potentials (Blue area represents the Ru-O path and red area represents the Ru-Cl path). The calculated spectra are matched well with the experimental results. The best fitting parameters are shown in Supplementary Table 7.

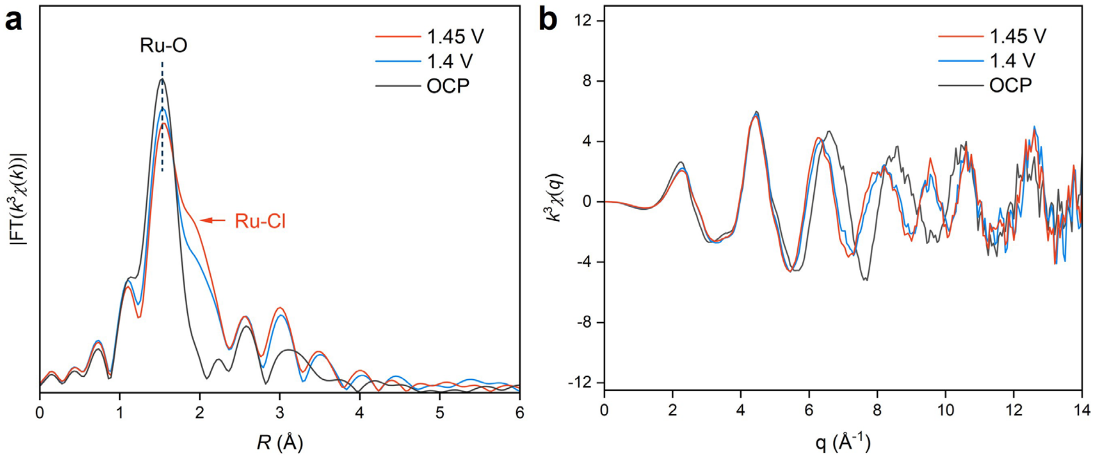

Supplementary Figure 38 | Operando k^3-weight Ru K-edge EXAFS spectra and the Fourier-transformed magnitudes for Ru-O_4 SAM at different applied potentials.

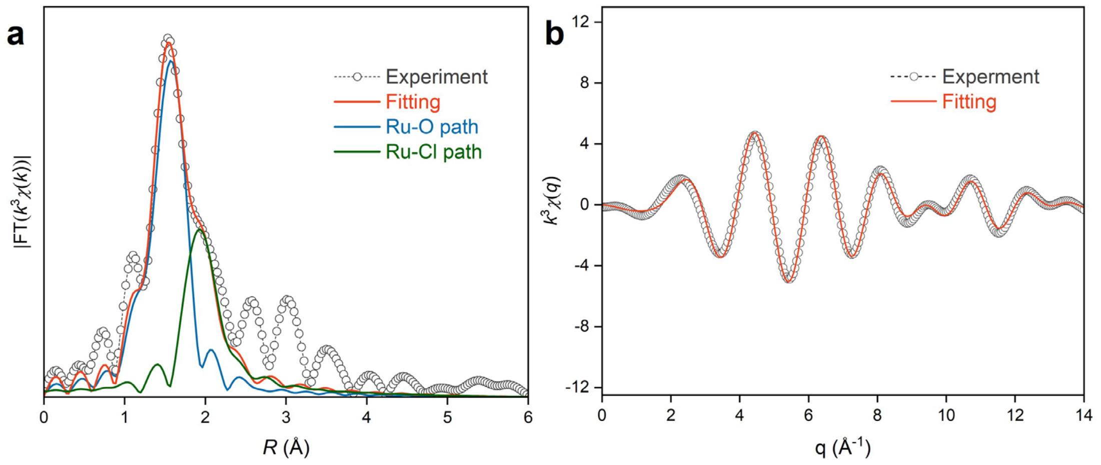

Supplementary Figure 39 | Fitting of k^3-weight Ru K-edge EXAFS curve for Ru-O_4 SAM at 1.4 V vs. RHE.

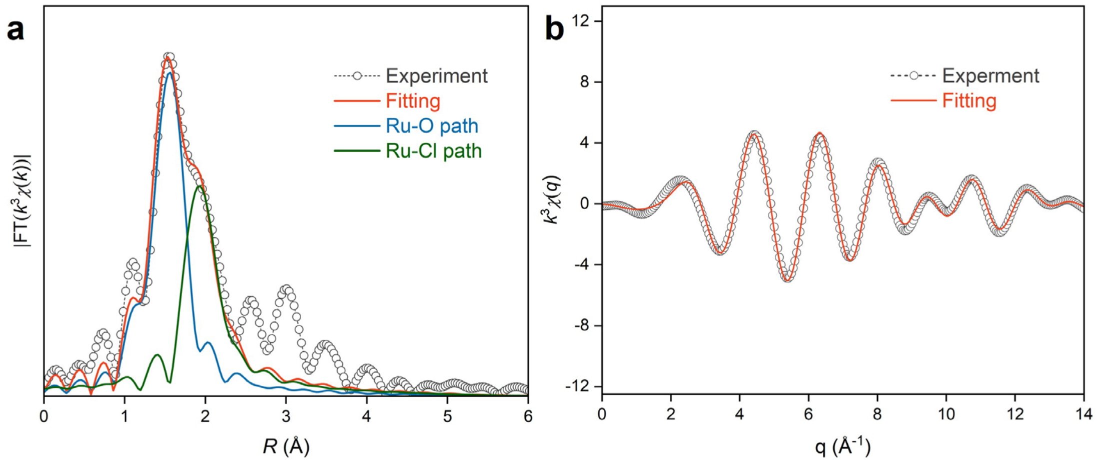

Supplementary Figure 40 | Fitting of k^3-weight Ru *K*-edge EXAFS curve for Ru-O_4 SAM at 1.45 V vs. RHE.

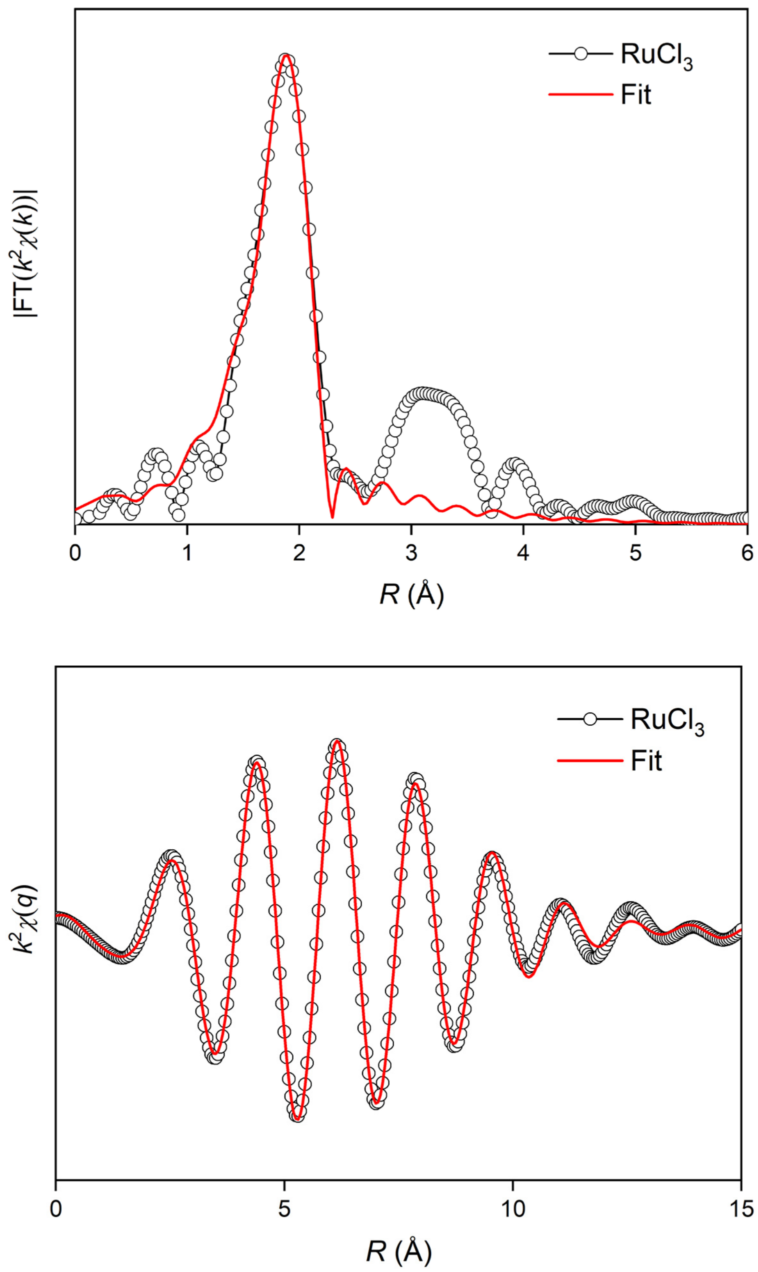

Supplementary Figure 41 | Ru *K*-edge EXAFS spectra of $RuCl_3$ standard sample. The calculated spectra are matched well with the experimental results. The best fitting parameters are shown in Supplementary Table 1.

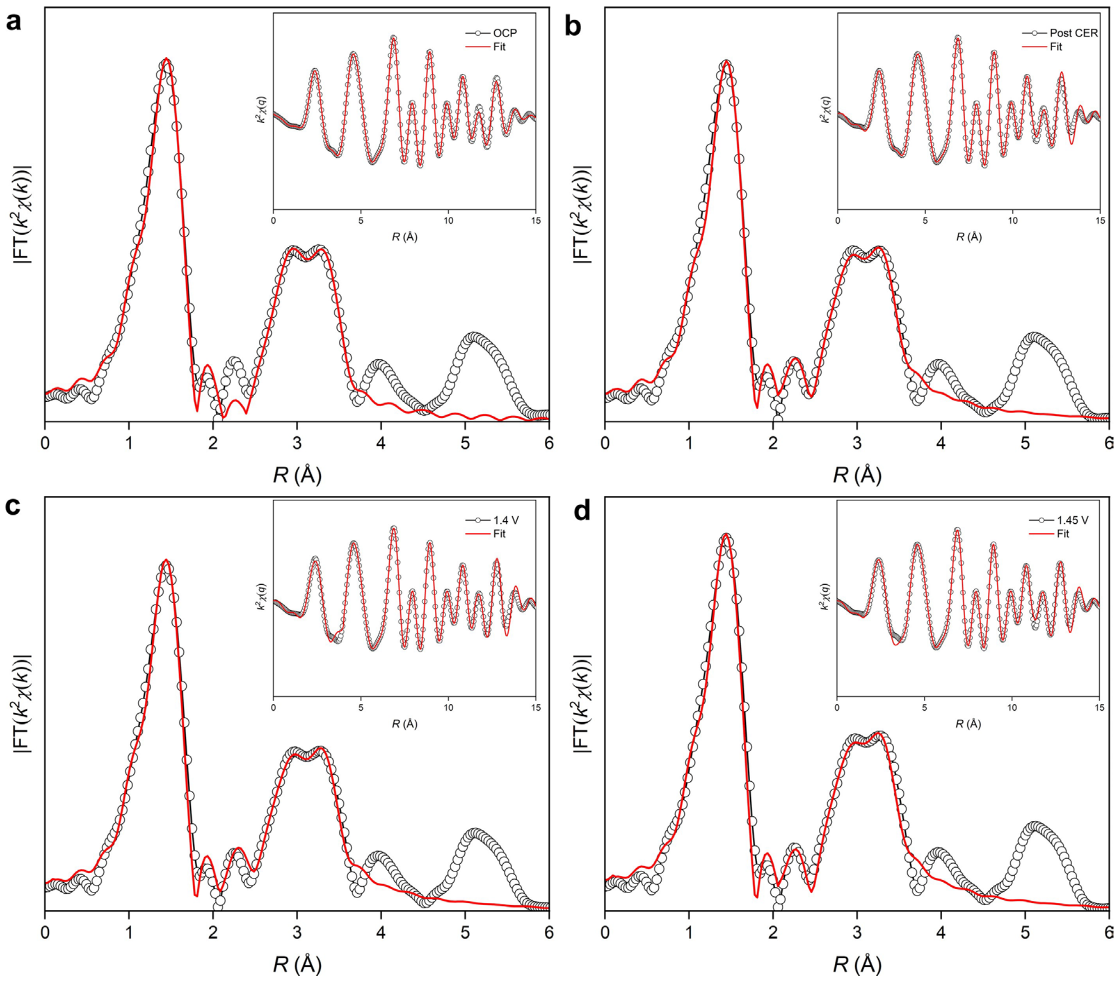

Supplementary Figure 42 | Operando Ru *K*-edge EXAFS spectra of RuO_2 at different applied potentials. The calculated spectra are matched well with the experimental results. The best fitting parameters are shown in Supplementary Table 9.

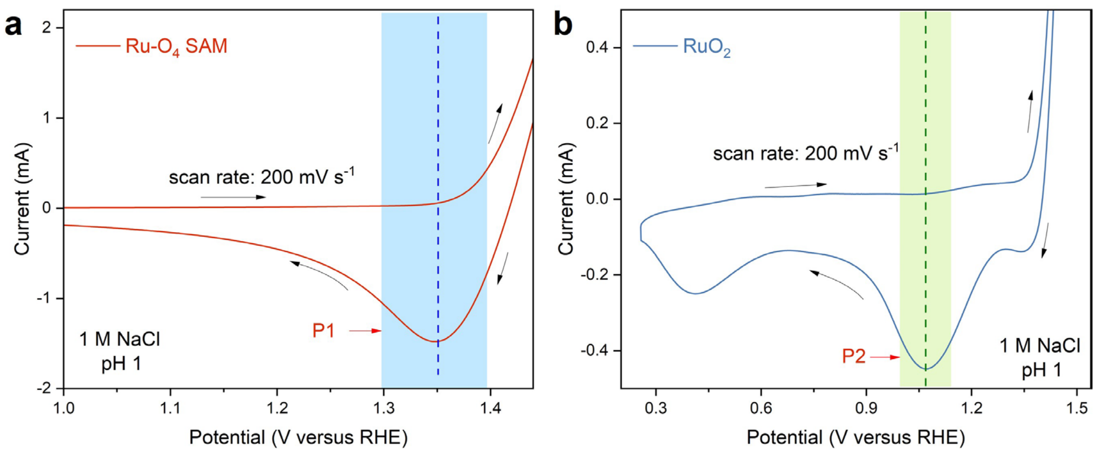

Supplementary Figure 43 | CV graph of Ru-O_4 SAM (a) and RuO_2 (b) in 1M NaCl with pH=1. The scan rate is 200 mV s^{-1} an electrode rotation speed of 1600 rpm.

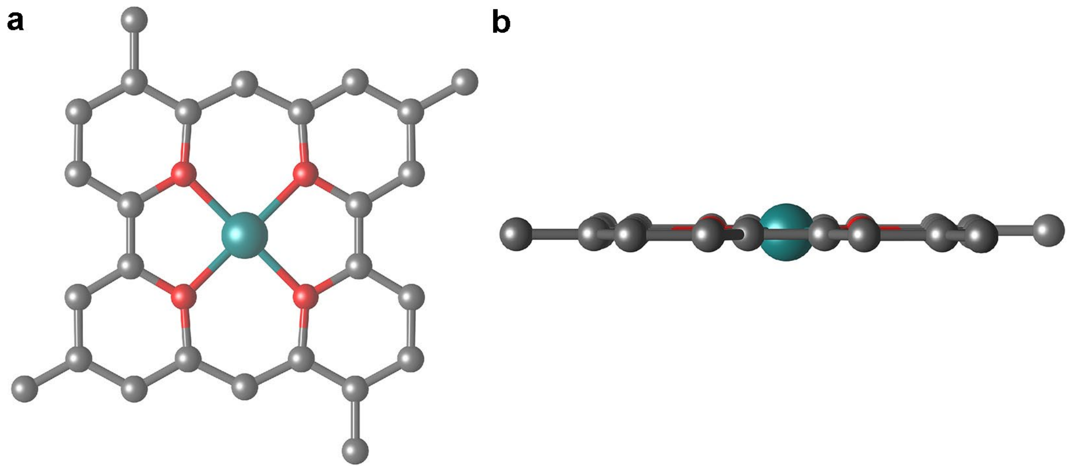

Supplementary Figure 44 | Modelled structure for RuO_4C_{10} moiety a, The top view of RuO_4C_{10} structure. **b,** The front view of RuO_4C_{10} structure. Supplementary Fig. 44 presents the modelled structure for RuO_4C_{10} moiety based on the EXAFs fitting and calculated formation energy. The grey, red, and dark cyan coloured spheres represent the carbon, oxygen, and ruthenium atoms, respectively.

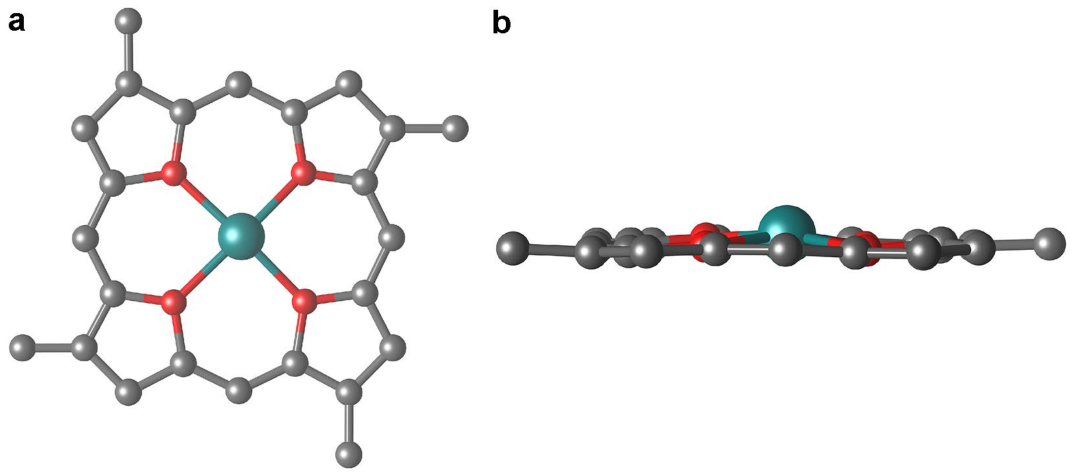

Supplementary Figure 45 | Modelled structure for RuO_4C_{12} moiety. a, Top view of RuO_4C_{12} structure. **b,** Front view of RuO_4C_{12} structure. Supplementary Fig. 45 presents the modelled structure for RuO_4C_{12} moiety based on the EXAFs fitting and calculated formation energy. The grey, red, and dark cyan coloured spheres represent the carbon, oxygen, and ruthenium atoms, respectively.

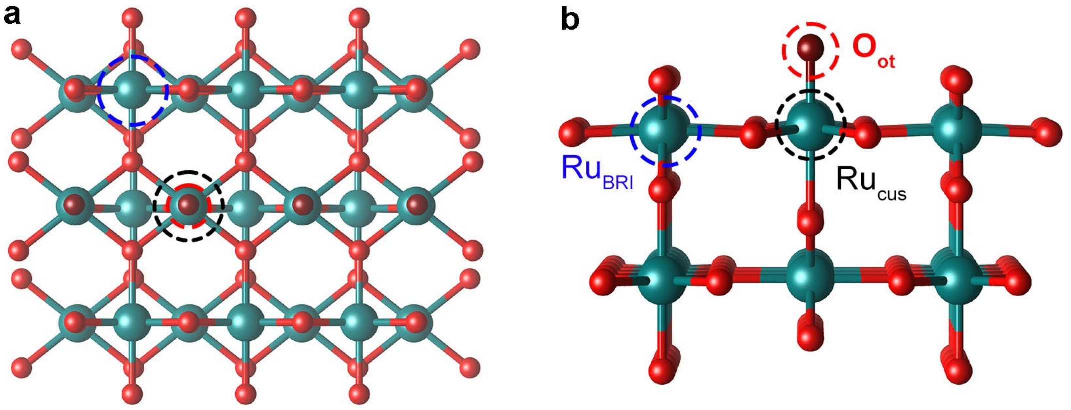

Supplementary Figure 46 | Modelled structure for RuO_2 (110). a, Top view of RuO_2 (110) structure. **b,** Left side view of RuO_2 (110) structure. Supplementary Fig. 46 presents the modelled structure for RuO_2 (110) structure, where terraces expose a fully bridge ruthenium site (Ru_{BRI}) and a coordinatively unsaturated site (Ru_{CUS}) with fivefold coordination. The red and dark cyan coloured spheres represent the oxygen and ruthenium atoms, respectively.

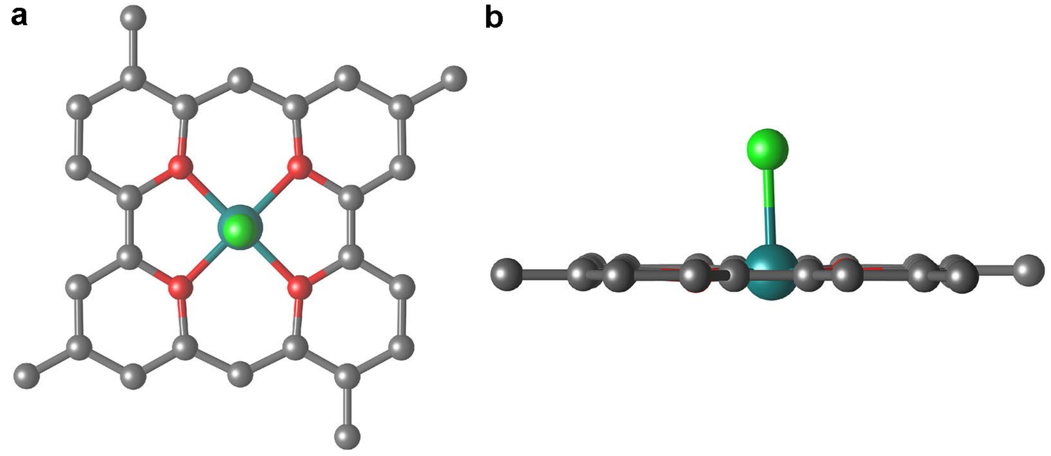

Supplementary Figure 47 | Modelled structure of *Cl intermediate over RuO_4C_{10} moiety. a, Top view of *Cl intermediate over RuO_4C_{10} moiety. **b,** Front view of *Cl intermediate over RuO_4C_{10} moiety. Supplementary Fig. 47 presents the modelled structure of *Cl intermediate over RuO_4C_{10} moiety based on operando experiment results. The grey, red, dark cyan, and green coloured spheres represent the carbon, oxygen, ruthenium, and chlorine atoms, respectively.

a b

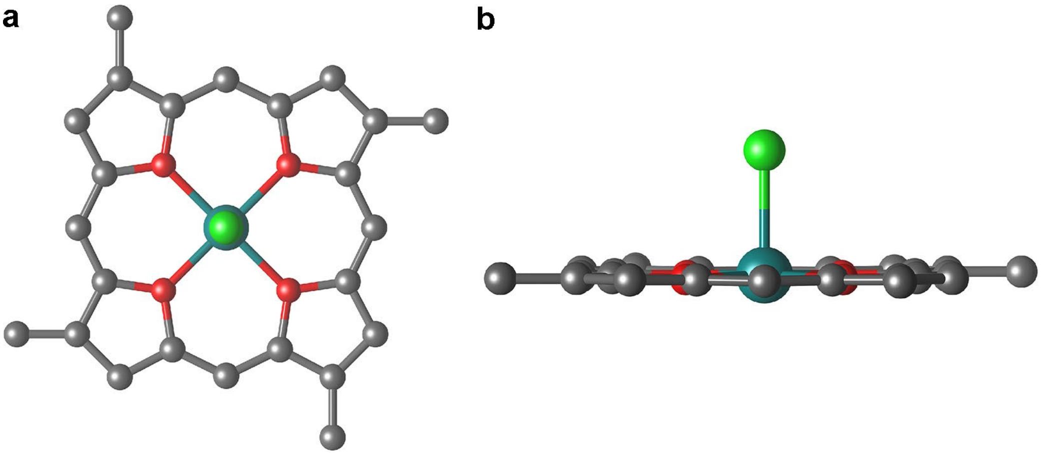

Supplementary Figure 48 | Modelled structure *Cl intermediate over RuO_4C_{12} moiety. a, Top view of *Cl intermediate over RuO_4C_{10} moiety. **b,** Front view of *Cl intermediate over RuO_4C_{10} moiety. Supplementary Fig. 48 presents the modelled structure of *Cl intermediate over RuO_4C_{12} moiety based on the operando experiment results. The grey, red, dark cyan, and green coloured spheres represent the carbon, oxygen, ruthenium, and chlorine atoms, respectively.

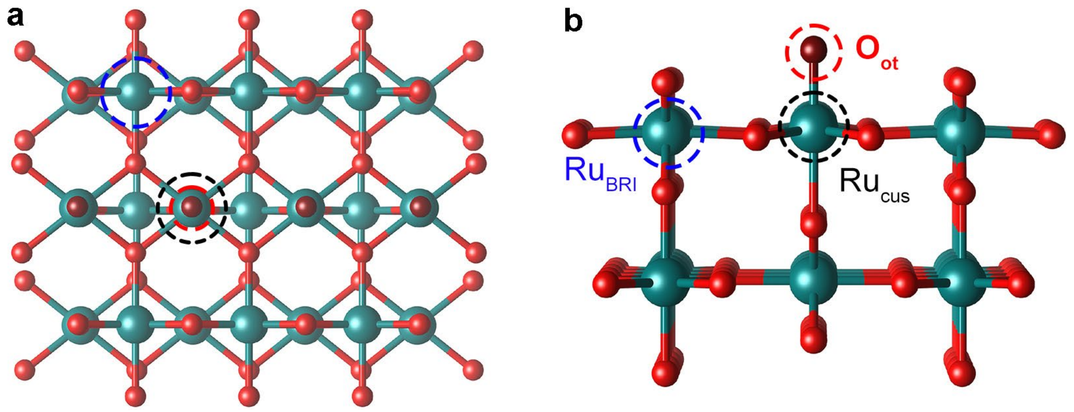

Supplementary Figure 49 | Modelled structure for RuO_2 (110) under CER conditions. a, Top view of RuO_2 (110) structure. **b,** Left side view of RuO_2 (110) structure under CER conditions. Supplementary Fig. 49 presents the modelled structure for RuO_2 (110) structure under typical CER operation conditions, where all coordinatively unsaturated Ru sites are capped by on-top oxygen O_{ot}. The red, dark red, and dark cyan coloured spheres represent the oxygen, adsorbed on-top oxygen, and ruthenium atoms, respectively.

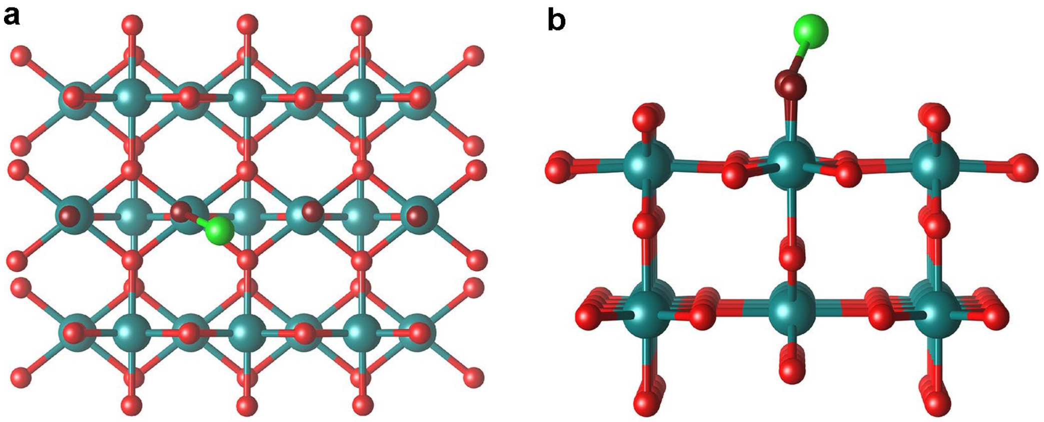

Supplementary Figure 50 | Modelled structure *OCl intermediate over RuO_2 (110).

a, Top view of *OCl intermediate over RuO_2 (110). **b,** Front view of *OCl intermediate over RuO_2 (110). Supplementary Fig. 50 presents the modelled structure of *OCl intermediate over RuO_2 (110) based on the operando experiment results and proposed literatures[7, 8]. The red, dark red, dark cyan, and green coloured spheres represent the oxygen, adsorbed on-top oxygen, ruthenium, and chlorine atoms, respectively.

a b

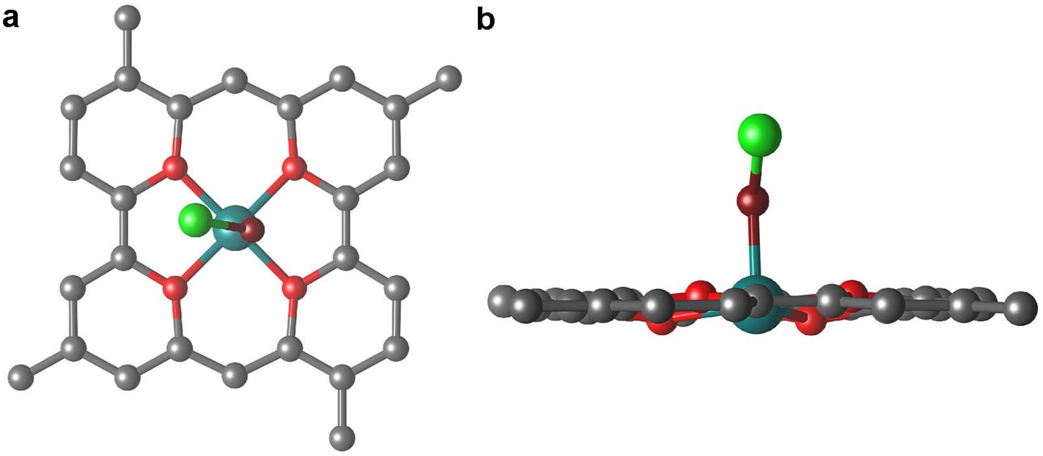

Supplementary Figure 51 | Modelled structure *OCl intermediate over RuO_4C_{10} moiety. a, Top view of *OCl intermediate over RuO_4C_{10} moiety. **b,** Front view of *OCl intermediate over RuO_4C_{10} moiety. The grey, red, dark red, dark cyan, and green coloured spheres represent the carbon, oxygen, adsorbed on-top oxygen, ruthenium, and chlorine atoms, respectively.

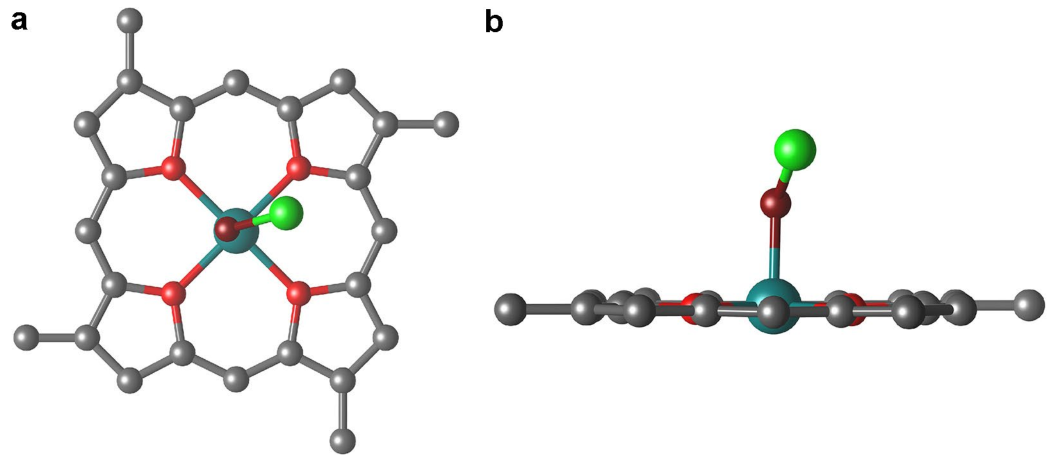

Supplementary Figure 52 | Modelled structure *OCl intermediate over RuO_4C_{12} moiety. a, Top view of *OCl intermediate over RuO_4C_{12} moiety. **b,** Front view of *OCl intermediate over RuO_4C_{12} moiety. The grey, red, dark red, dark cyan, and green coloured spheres represent the carbon, oxygen, adsorbed on-top oxygen, ruthenium, and chlorine atoms, respectively.

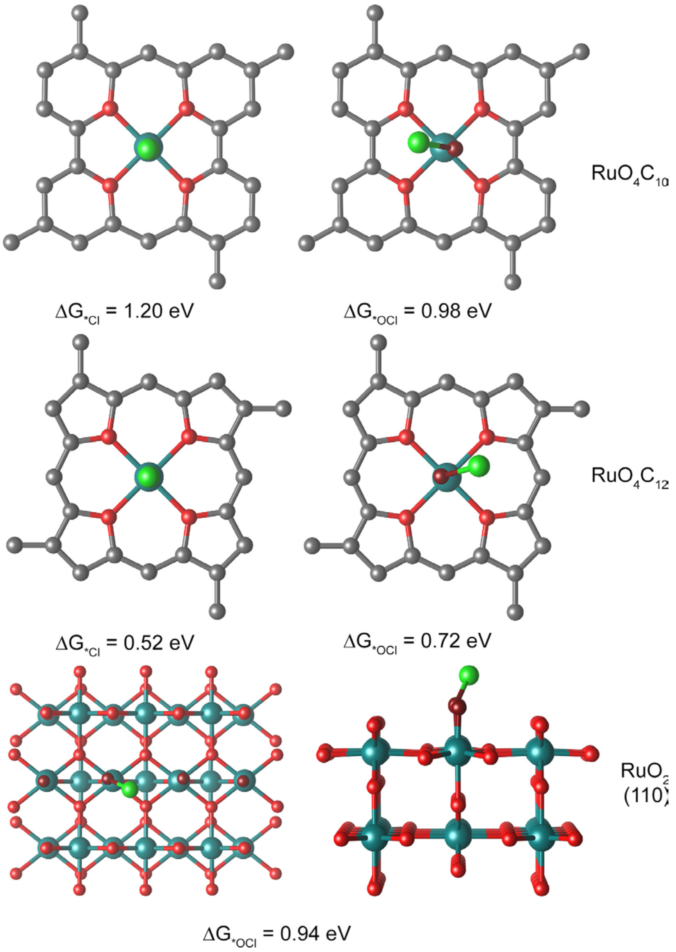

Supplementary Figure 53 | Modelled plausible structures of adsorbate intermediates (*Cl and *OCl) for RuO_4C_{10}, RuO_4C_{12}, and RuO_2 (110) during CER process and the corresponding Gibbs free energies of adsorbates.

The *Cl adsorbate intermediates for RuO_4C_{10} and RuO_4C_{12} during CER process are determined based on the operando experiment results. The *OCl adsorbate intermediate for RuO_2 (110) is modelled through both operando experiment results and previously reported literatures[8]. To have a more comprehensive comparison between RuO_4 SAM and RuO_2 (110), we also constructed the *OCl adsorbate intermediate for RuO_4 SAM. The corresponding Gibbs free energies of different adsorbates are shown in Supplementary Fig. 53. The grey, red, dark red, dark cyan, and green coloured spheres represent the carbon, oxygen, adsorbed on-top oxygen, ruthenium, and chlorine atoms, respectively.

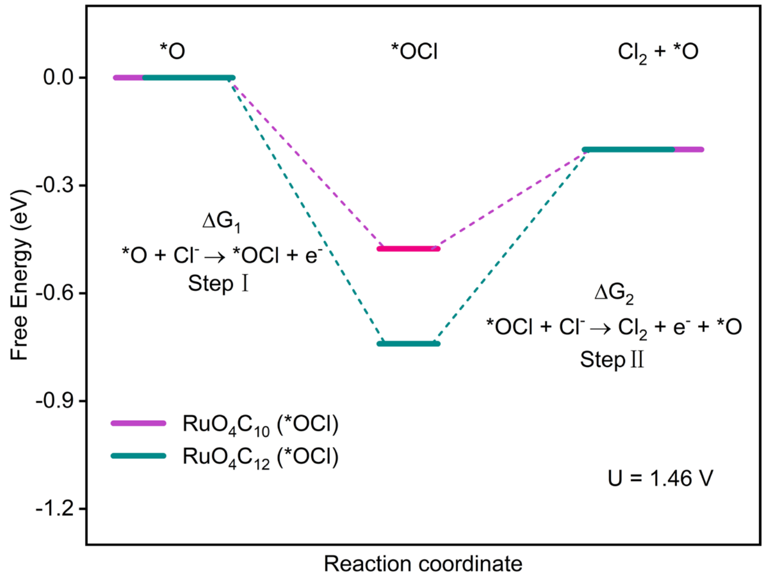

Supplementary Figure 54 | DFT calculations for evaluating the CER activity over RuO_4C_{10} and RuO_4C_{12} moiety through *OCl intermediate at the applied voltage of 1.46 V vs. RHE.

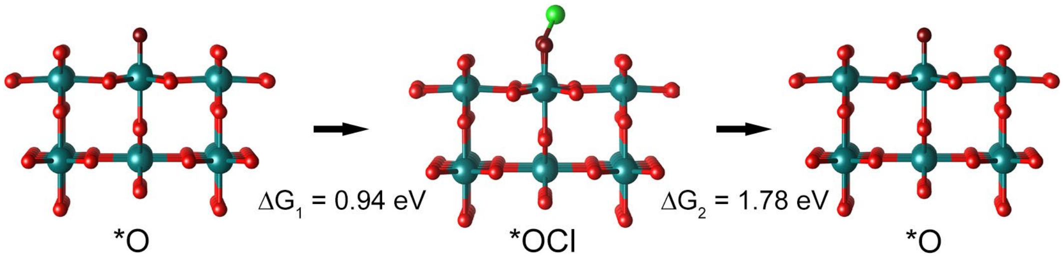

Supplementary Figure 55 | Modelled structures of adsorbate intermediates (*OCl) over RuO_2 (110) and free energy changes during CER process at an applied voltage of 0 V. The grey, red, dark red, dark cyan, and green coloured spheres represent the carbon, oxygen, adsorbed on-top oxygen, ruthenium, and chlorine atoms, respectively.

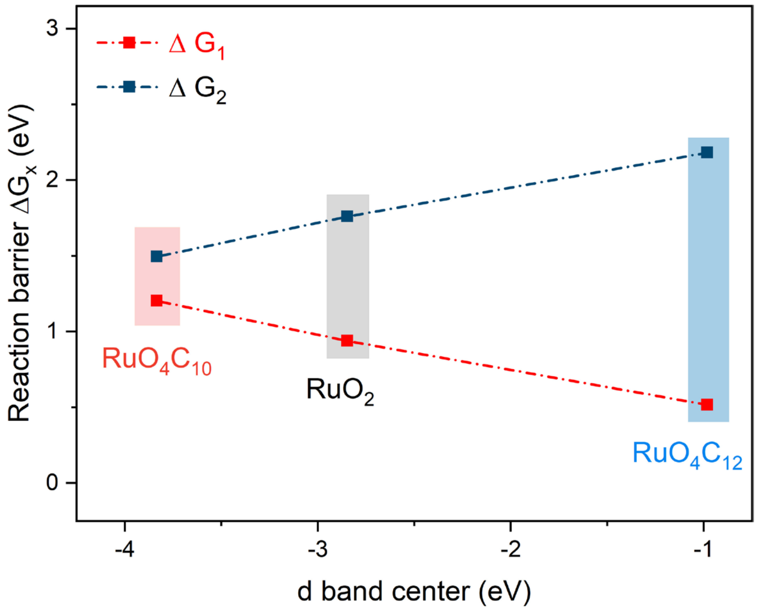

Supplementary Figure 56 | Gibbs free energy changes for two steps over the CER process versus *d* band center (ε_d) of the RuO_4C_{10}, RuO_4C_{12}, and RuO_2 (110).

Supplementary Fig. 56 exhibits the relations between the ε_d of Ru 4*d* orbitals and Gibbs free-energy changes of different steps, which indicates that the more negative ε_d leads to a weaker adsorption of chloride species and Ru active sites, thus resulting in a low Gibbs free-energy change for CER.

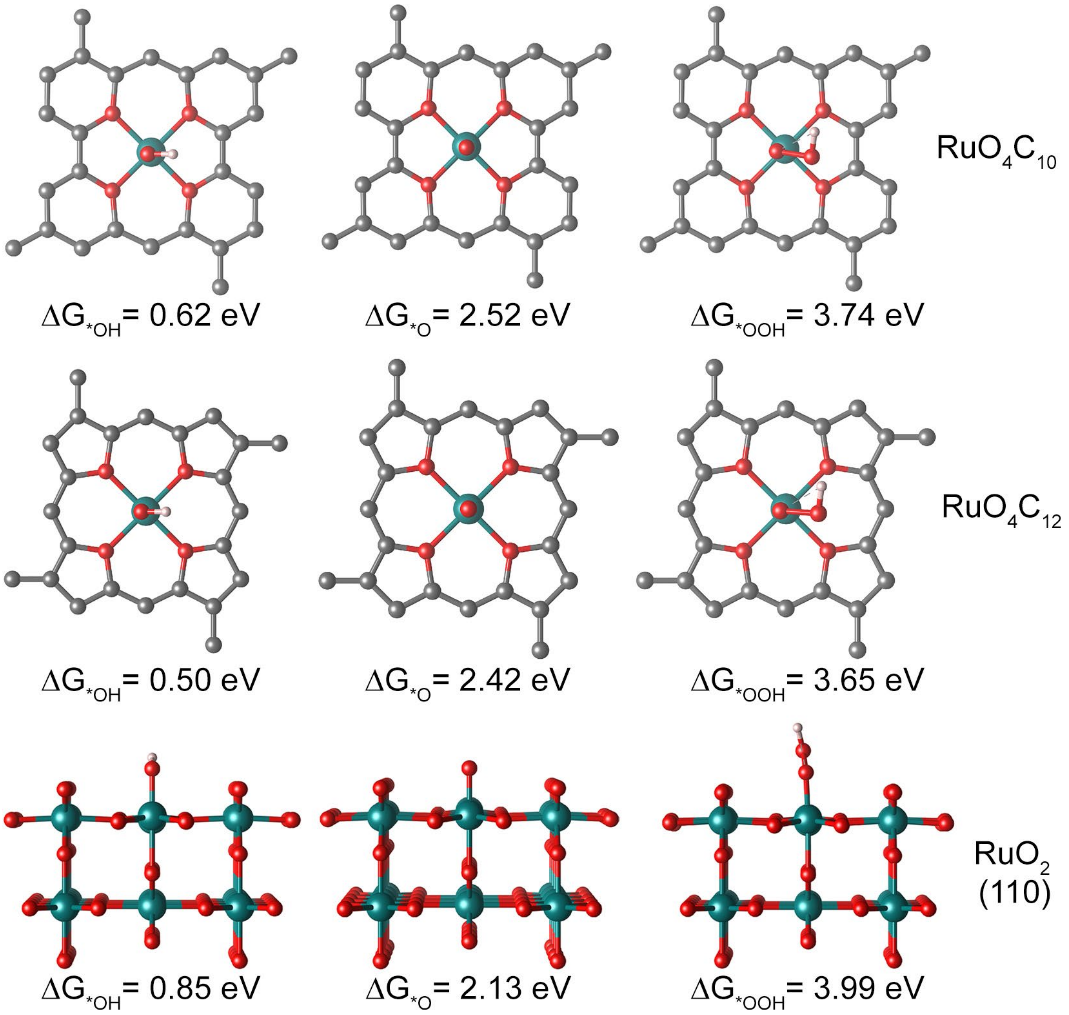

Supplementary Figure 57 | Modelled plausible structures of adsorbate intermediates (*OH, *O, and *OOH) for RuO_4C_{10}, RuO_4C_{12}, and RuO_2 (110) during OER process and the corresponding Gibbs free energies of adsorbates.

Supplementary Fig. 57 illustrates all the possible adsorbate intermediates of RuO_4C_{10}, RuO_4C_{12}, and RuO_2 (110) during OER process. The corresponding Gibbs free energies of different adsorbates are presented in Supplementary Fig. 45. The grey, red, pink, and dark cyan coloured spheres represent the carbon, oxygen, hydrogen, and ruthenium atoms, respectively.

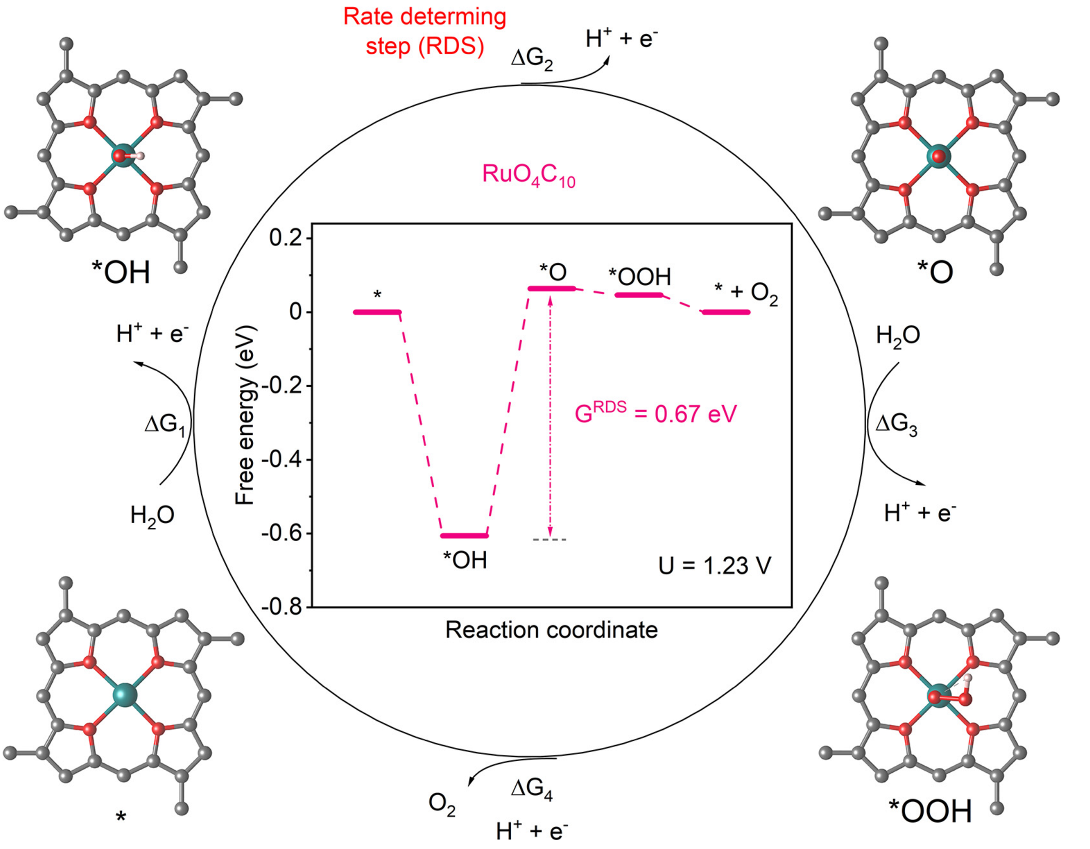

Supplementary Figure 58 | DFT calculations for evaluating the OER activity over RuO_4C_{10} moiety at an applied voltage of 1.23 V and the corresponding four-step OER reaction pathways.

Supplementary Fig. 58 presents the free energy diagram for OER over RuO_4C_{10} moiety. The formation of *O through *OH → *O + H^+ + e^- is determined as the potential-determining step (PDS) with ΔG of 0.67 eV.

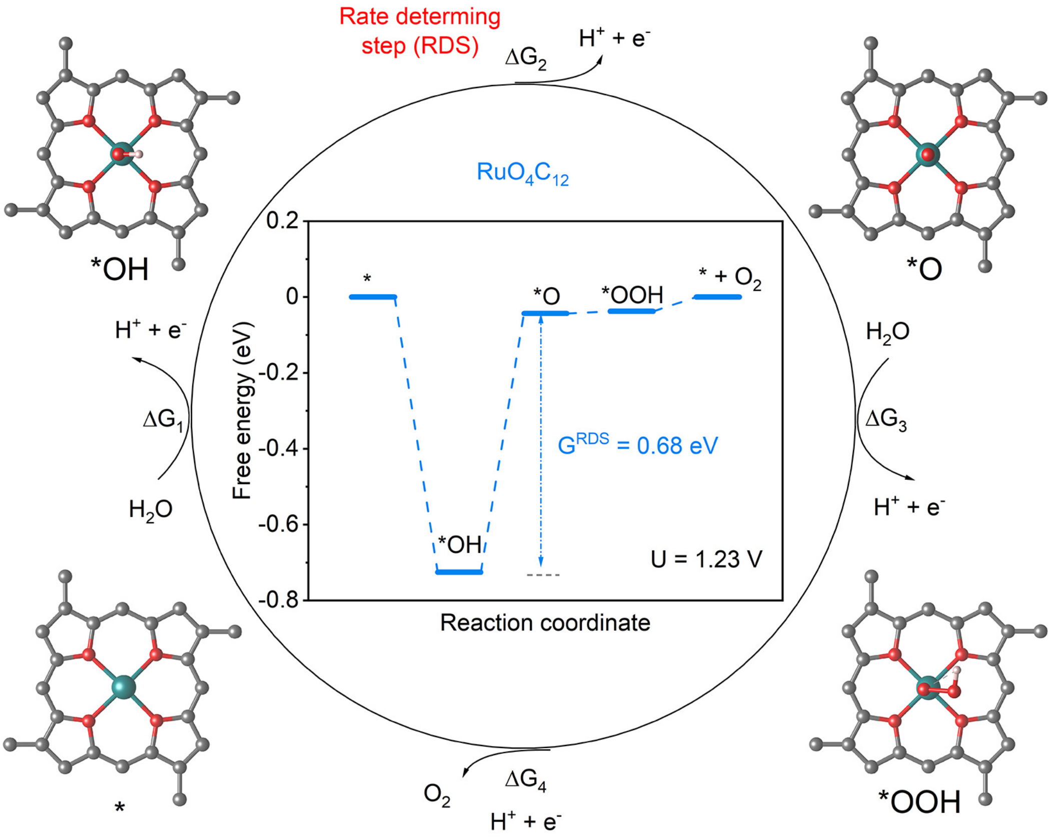

Supplementary Figure 59 | DFT calculations for evaluating the OER activity over RuO_4C_{10} moiety at an applied voltage of 1.23 V vs. RHE and the corresponding four-step OER reaction pathways.

Supplementary Fig. 59 presents the free energy diagram for OER over RuO_4C_{12} moiety. The formation of *O through *OH → *O + H^+ + e^- is determined as the PDS with ΔG of 0.68 eV.

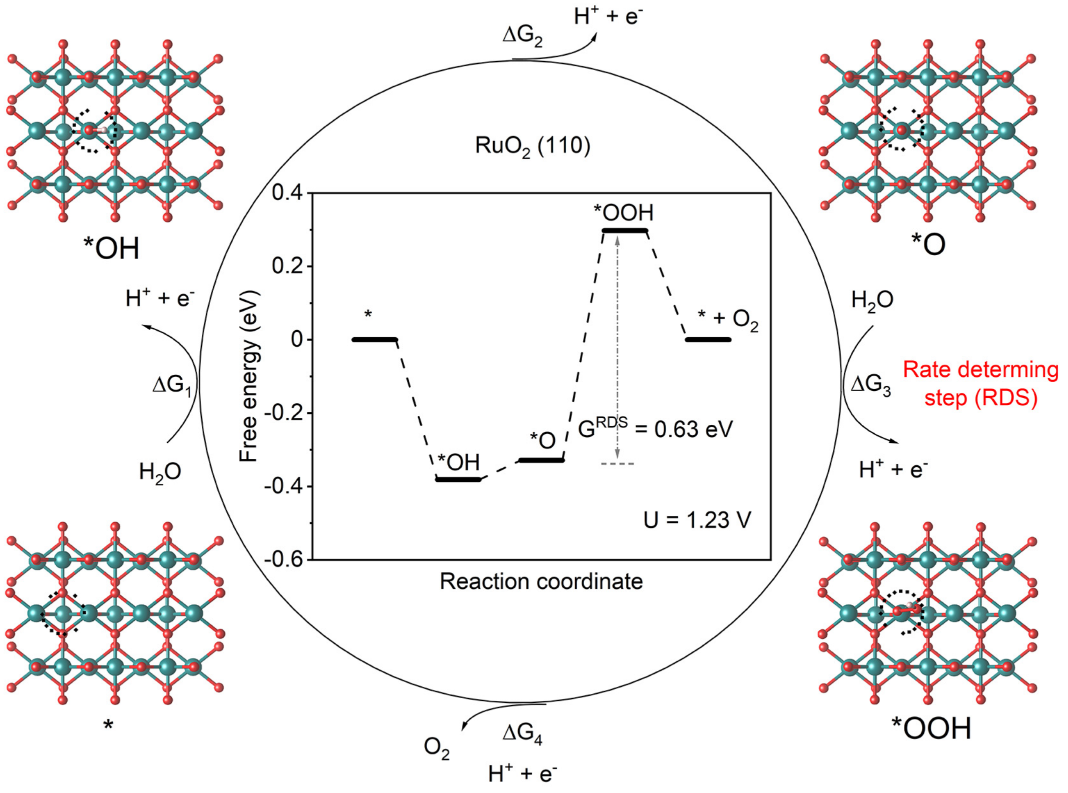

Supplementary Figure 60 | DFT calculations for evaluating the OER activity over RuO_2 (110) moiety at applied voltage of 1.23 V vs. RHE and the corresponding four-step OER reaction pathways.

Supplementary Fig. 60 presents the free energy diagram for OER over RuO_4C_{10} moiety. The formation of *OOH via *O + H_2O → *OOH + H^+ + e^- is determined as the PDS with ΔG of 0.63 eV.

Supplementary Table 1 | Fitting parameters of Ru *K*-edge EXAFS curve for Ru-O_4 SAM and standard sample of $RuCl_3$ and Ru foil.

Sample	Path	^aN	^bR(Å)	$^c\sigma^2$ (10^{-3}Å^2)	$^d\Delta E_0$ (eV)	^eR_f
$RuCl_3$	Ru-Cl	6	2.34 ± 0.03	5.5 ± 1.6	2.9 ± 1.6	0.007
Ru foils	Ru-Ru	12	2.65 ± 0.01	4.3 ± 0.8	7.2 ±1.8	0.0004
Ru-O_4	Ru-O	3.8 ± 0.3	1.99 ± 0.01	2.9 ± 0.5	3.2 ± 0.8	0.007

$^a\boldsymbol{N}$, Coordination number; $^b\boldsymbol{R}$, bonding distance; $^c\boldsymbol{\sigma^2}$, Debye-Waller factor to account for both thermal and structural disorders; $^d\boldsymbol{\Delta E_0}$, inner potential correction; $^e\boldsymbol{R_f}$, the goodness of the fitting.

Supplementary Table 2 | The results of DFT calculated formation energies of different structure models (U = 1.46 V vs. SHE and U = 0 V vs. SHE).

	U = 1.46 V vs. SHE		U =0 V vs. SHE	
	O_xC_y-Ru-C_{10}	O_xC_y-Ru- C_{12}	O_xC_y-Ru-C_{10}	O_xC_y-Ru- C_{12}
C_4	-0.02 eV	1.3 eV	-1.6 eV	-0.23 eV
OC_3	-3.2 eV	-2.2 eV	-4.7 eV	-3.8 eV
O_2C_2[a]	-3.7 eV	-3.4 eV	-5.3 eV	-5.0 eV
O_2C_2[b]	-4.5 eV	-4.6 eV	-6.1 eV	-6.2 eV
O_3C	-4.7 eV	-5.7 eV	-6.2 eV	-7.2 eV
O_4	-5.0 eV	-6.1 eV	-6.5 eV	-7.7 eV

Supplementary Table 3 | Cl_2 selectivity of Ru-O_4 SAM, CS-Ru NPs, and DSA measured by iodometric titration.

Sample	Current density (mA cm^{-2})	Electrode area (cm^2)	Theoretical generated Cl_2 (μmol)	Experimental generated Cl_2 (μmol)	Cl_2 selectivity (%)
Ru-O_4 SAM	10	0.24	2.49	2.48, 2.45, 2.47	99.1
CS-Ru NPs	10	0.24	2.49	2.35, 2.25, 2.42	94.0
DSA	10	1	10.36	9.25, 9.17, 9.55	90

The experimental generated Cl_2 is measured through the iodometric titration. The current density was maintained at 10 mA cm^{-2} for 200s.

Supplementary Table 4 | Comparison of CER activity of Ru-O_4 SAM and recently reported active catalysts.

Samples	Operation conditions	Overpotential at 10 mA cm^{-2}	Cl_2 selectivity	Ref
Ru-O_4 SAM	pH=1, 1 M NaCl	30 mV	99%	This work
CS-Ru NPs	pH=1, 1 M NaCl	110 mV	94%	
Commercial DSA	pH=1, 1 M NaCl	85 mV	95.5%	
RuO_2 NPs/TiO_2 NBs	pH=3, Saturated NaCl	70 mV	90.3%	10
RuO_2@TiO_2 NSAs	pH=2, Saturated NaCl	72.2 mV	90%	11
Mesoporous 15 wt% RuO_2 /TiO_2	pH = 3, 4 M NaCl	220 mV	NA	12
Ti/$Ir_{0.8}Nd_{0.2}O_x$	pH=2, 5 M NaCl	60 mV	91%	13
Ru-Ti-Ir oxide/Ti	pH=2, 4 M NaCl	125.2 mV	97%	14
Mesoporous 15 wt% (Ru-Ir)/TiO_2	pH=3, 4 M NaCl	140 mV	NA	12
Ti/RuO_2–IrO_2- Sb_2O_5–SnO_2	seawater	75 mV	71-87%	15
$(Ru_{0.3}Ti_{0.34}Sn_{0.3}Sb_{0.06})$ O_2–TNTs	pH=2, 5 M NaCl	110 mV	NA	16
RuO_2/Lignin-based carbon electrode	Saturated NaCl	64 mV	NA	17
Pt_1/CNT	pH=0.9, 1 M NaCl	50 mV	97.1%	9
$CoSb_2O_x$	pH=2, 4 M NaCl	430 mV	97%	18
$MnSb_2O_x$	pH=2, 4 M NaCl	470 mV	89.9%	
Co_3O_4 /FTO	pH=3, Saturated NaCl	200 mV	90%	19
Irn-TiC	pH=2, 4 M NaCl	31 mV	98%	20

NB, Nanobelt; NSA, Nanosheet array; TNT, Titanium nanotube; FTO, Fluorine-doped tin oxide; CNT, Carbon nanotube

Supplementary Table 5 | Fitting parameters of k^2-weight Ru K-edge EXAFS curve for Ru-O_4 SAM after long term stability test.

Sample	Path	^aN	^bR(Å)	$^c\sigma^2$ (10^{-3}Å^2)	$^d\Delta E_0$ (eV)	^eR_f
Initial	Ru-O	3.8 ± 0.2	1.99 ± 0.01	3.8 ± 1.2	3.6 ± 1.1	0.006
100 h	Ru-O	3.8 ± 0.3	2.0 ± 0.01	4.6 ± 1.1	2.3 ± 1.2	0.002
1000 h	Ru-O	3.9 ± 0.4	2.0 ± 0.01	3.9 ± 0.8	4.1 ± 1.5	0.002

$^a\boldsymbol{N}$, Coordination number; $^b\boldsymbol{R}$, bonding distance; $^c\boldsymbol{\sigma^2}$, Debye-Waller factor to account for both thermal and structural disorders; $^d\boldsymbol{\Delta E_0}$, inner potential correction; $^e\boldsymbol{R_f}$**,** the goodness of the fitting.

Supplementary Table 6 | DFT calculations for the Raman band in Cl-RuO_4C_{10} moiety.

Configuration	Raman shift (cm^{-1})
Cl-Ru-O_4C_{10}	139; 327

Supplementary Table 7| Fitting parameters of k^2-weight Ru *K*-edge EXAFS curve for Ru-O_4 SAM at selected potentials.

Sample	Path	^aN	^bR(Å)	$^c\sigma^2$ (10^{-3}Å^2)	$^d\Delta E_0$ (eV)	^eR_f
Ru-O_4 OCP	Ru-O	3.8 ± 0.2	1.99 ± 0.01	3.8 ± 1.2	3.6 ± 1.1	0.006
1.4 V	Ru-O	3.8 ± 0.4	2.05 ± 0.03	3.7 ± 0.7	5.6 ± 1.0	0.005
	Ru-Cl	1.1 ± 0.3	2.37 ± 0.02	6.2 ± 1.9	5.6 ± 1.0	0.005
1.45 V	Ru-O	3.7 ± 0.5	2.07 ± 0.03	3.3 ± 1.2	5.9 ± 1.5	0.008
	Ru-Cl	1.4 ± 0.4	2.36 ± 0.03	5.6 ± 1.8	5.9 ± 1.5	0.008
Post CER	Ru-O	3.7 ± 0.3	2.01 ± 0.02	3.2 ± 0.9	3.9 ± 0.9	0.002

^{a}***N***, Coordination number; ^{b}***R***, bonding distance; $^c\boldsymbol{\sigma^2}$, Debye-Waller factor to account for both thermal and structural disorders; $^d\boldsymbol{\Delta E_0}$, inner potential correction; $^e\boldsymbol{R_f}$, the goodness of the fitting.

Supplementary Table 8 | Fitting parameters of k^3-weight Ru *K*-edge EXAFS curve for Ru-O_4 SAM at selected potentials.

Sample	Path	^{a}N	^{b}R(Å)	$^{c}\sigma^2$ (10^{-3}Å^2)	$^{d}\Delta E_0$ (eV)	$^{e}R_f$
1.4 V	Ru-O	3.9 ± 0.5	2.07 ± 0.04	4.7 ± 1.7	3.8 ± 1.2	0.007
	Ru-Cl	1.2 ± 0.2	2.39 ± 0.03	5.8 ± 2.1	3.8 ± 1.2	0.007
1.45 V	Ru-O	3.8 ± 0.4	2.07 ± 0.03	4.5 ± 1.2	5.2 ± 1.5	0.004
	Ru-Cl	1.5 ± 0.2	2.38 ± 0.04	5.2 ± 1.9	5.2 ± 1.5	0.004

$^{a}\boldsymbol{N}$, Coordination number; $^{b}\boldsymbol{R}$, bonding distance; $^{c}\boldsymbol{\sigma^2}$, Debye-Waller factor to account for both thermal and structural disorders; $^{d}\boldsymbol{\Delta E_0}$, inner potential correction; $^{e}\boldsymbol{R_f}$**,** the goodness of the fitting.

Supplementary Table 9 | Fitting parameters of k^2-weight Ru *K*-edge EXAFS curve for RuO_2 at selected potentials.

Sample	Path	^aN	^bR(Å)	$^c\sigma^2$ (10^{-3}Å^2)	$^d\Delta E_0$ (eV)	^eR_f
RuO_2 OCP	Ru-O_1	5.9 ± 0.4	1.97*	4.8 ± 1.2	-1.0 ± 0.6	0.003
	Ru-Ru_1	1.9 ± 0.5	3.15 ± 0.02	9.7 ± 2.1	6.8*	0.003
	Ru-Ru_2	4.5 ± 0.8	3.55 ± 0.03	7.9 ± 1.6	6.8*	0.003
1.4 V	Ru-O_1	5.8 ± 0.5	1.97*	5.1 ± 1.1	-0.9 ± 0.4	0.007
	Ru-Ru_1	1.8 ± 0.3	3.16 ± 0.03	10.1 ± 2.5	6.8*	0.007
	Ru-Ru_2	4.3 ± 1.0	3.56 ± 0.03	8.2 ± 1.9	6.8*	0.007
1.45 V	Ru-O_1	5.8 ± 0.6	1.97 *	5.3 ± 1.5	-1.5 ± 0.8	0.005
	Ru-Ru_1	1.8 ± 0.4	3.16 ± 0.04	10.3 ± 2.7	6.8*	0.005
	Ru-Ru_2	4.4 ± 0.9	3.55 ± 0.03	8.3 ± 2.3	6.8*	0.005
Post CER	Ru-O_1	5.9 ± 0.5	1.97 *	4.9 ± 1.0	-1.9 ± 0.9	0.002
	Ru-Ru_1	1.9 ± 0.3	3.16 ± 0.02	9.9 ± 2.8	6.8*	0.002
	Ru-Ru_2	4.6 ± 1.1	3.55 ± 0.03	8.0 ± 1.7	6.8*	0.002

$^a\boldsymbol{N}$, Coordination number; $^b\boldsymbol{R}$, bonding distance; $^c\boldsymbol{\sigma^2}$, Debye-Waller factor to account for both thermal and structural disorders; $^d\boldsymbol{\Delta E_0}$, inner potential correction; $^e\boldsymbol{R_f}$, the goodness of the fitting. ***,** This parameter is fixed. O_1, O_2, Ru_1, and Ru_2 represent the first and second nearest neighbor coordination shells of O and Ru atoms, respectively.

Reference

1. Li ZQ, Lu CJ, Xia ZP, Zhou Y, Luo Z. X-ray diffraction patterns of graphite and turbostratic carbon. *Carbon* **45**, 1686-1695 (2007).

2. Liu D*, et al.* Atomically dispersed platinum supported on curved carbon supports for efficient electrocatalytic hydrogen evolution. *Nat. Energy* **4**, 512-518 (2019).

3. Cored J*, et al.* Hydrothermal synthesis of ruthenium nanoparticles with a metallic core and a ruthenium carbide shell for low-temperature activation of CO_2 to methane. *J. Am. Chem. Soc.* **141**, 19304-19311 (2019).

4. Wang W*, et al.* Hydrous ruthenium oxide nanoparticles anchored to graphene and carbon nanotube hybrid foam for supercapacitors. *Sci. Rep.* **4**, 4452 (2014).

5. Pilla AS, Cobo EO, Duarte MME, Salinas DR. Evaluation of anode deactivation in chlor-alkali cells. *J. Appl. Electrochem.* **27**, 1283-1289 (1997).

6. Shan J*, et al.* Short-range ordered iridium single atoms integrated into cobalt oxide spinel structure for highly efficient electrocatalytic water oxidation. *J. Am. Chem. Soc.* **143**, 5201-5211 (2021).

7. Exner KS, Anton J, Jacob T, Over H. Controlling selectivity in the chlorine evolution reaction over RuO_2-based catalysts. *Angew. Chem. Int. Ed.* **53**, 11032-11035 (2014).

8. Exner KS, Anton J, Jacob T, Over H. Full kinetics from first principles of the chlorine evolution reaction over a RuO_2(110) model electrode. *Angew. Chem. Int. Ed.* **55**, 7501-7504 (2016).

9. Lim T*, et al.* Atomically dispersed Pt–N_4 sites as efficient and selective

electrocatalysts for the chlorine evolution reaction. *Nat. Commun.* **11**, 412 (2020).

10. Huang J*, et al.* RuO_2 nanoparticles decorate belt-like anatase TiO_2 for highly efficient chlorine evolution. *Electrochim. Acta* **339**, 135878 (2020).

11. Jiang M*, et al.* Superaerophobic RuO_2-Based nanostructured electrode for high-performance chlorine evolution reaction. *Small* **13**, 1602240 (2017).

12. Menzel N, Ortel E, Mette K, Kraehnert R, Strasser P. Dimensionally stable Ru/Ir/TiO_2-anodes with tailored mesoporosity for efficient electrochemical chlorine evolution. *ACS Catal.* **3**, 1324-1333 (2013).

13. Hu J, Xu H, Feng X, Lei L, He Y, Zhang X. Neodymium-doped IrO_2 electrocatalysts supported on titanium plates for enhanced chlorine evolution reaction performance. *ChemElectroChem* **8**, 1204-1210 (2021).

14. Zeradjanin AR, Menzel N, Schuhmann W, Strasser P. On the faradaic selectivity and the role of surface inhomogeneity during the chlorine evolution reaction on ternary Ti–Ru–Ir mixed metal oxide electrocatalysts. *Phys. Chem. Chem. Phys.* **16**, 13741-13747 (2014).

15. Wang S, Xu H, Yao P, Chen X. Ti/RuO_2-IrO_2-SnO_2-Sb_2O_5 anodes for Cl_2 evolution from seawater. *Electrochemistry* **80**, 507-511 (2012).

16. Xiong K*, et al.* Sn and Sb co-doped RuTi oxides supported on TiO_2 nanotubes anode for selectivity toward electrocatalytic chlorine evolution. *J. Appl. Electrochem.* **43**, 847-854 (2013).

17. Chi M*, et al.* Lignin-based monolithic carbon electrode decorating with RuO_2 nanospheres for high-performance chlorine evolution reaction. *Ind. Crops Prod.* **159**, 113088 (2021).

18. Moreno-Hernandez IA, Brunschwig BS, Lewis NS. Crystalline nickel, cobalt, and manganese antimonates as electrocatalysts for the chlorine evolution reaction. *Energy Environ. Sci.* **12**, 1241-1248 (2019).

19. Zhu X*, et al.* Co_3O_4 nanobelt arrays assembled with ultrathin nanosheets as highly efficient and stable electrocatalysts for the chlorine evolution reaction. *J. Mater. Chem. A* **6**, 12718-12723 (2018).

20. Yang J, Li W-H, Xu K, Tan S, Wang D, Li Y. Regulating the tip effect on single-atom and cluster catalysts: forming reversible oxygen species with high efficiency in chlorine evolution reaction. *Angew. Chem. Int. Ed.* **61,** e202200366 (2022).
