# Supplementary material for: Electrosynthesis of chlorine from seawater-like solution through single-atom catalysts
Source: Nat Commun. 2023 Apr 29;14:2475. doi: 10.1038/s41467-023-38129-w (PMC10148798; doi:10.1038/s41467-023-38129-w)
Supplement: Supplementary file 3 — Description of Additional Supplementary Information [file 41467_2023_38129_MOESM3_ESM.pdf]

Description of Additional Supplementary Files

Supplementary Video 1: A movie shows the continuously production of Cl2 gas
