# Supplementary material for: Electrosynthesis of chlorine from seawater-like solution through single-atom catalysts
Source: Nat Commun. 2023 Apr 29;14:2475. doi: 10.1038/s41467-023-38129-w (PMC10148798; doi:10.1038/s41467-023-38129-w)
Supplement: Supplementary file 2 — Peer review file [file 41467_2023_38129_MOESM2_ESM.pdf]

nature portfolio

Peer Review File

Electrosynthesis of chlorine from seawater-like solution through single-atom catalysts

Open Access This file is licensed under a Creative Commons Attribution 4.0 International License, which permits use, sharing, adaptation, distribution and reproduction in any medium or format, as long as you give appropriate credit to the original author(s) and the source, provide a link to the Creative Commons license, and indicate if changes were made. In the cases where the authors are anonymous, such as is the case for the reports of anonymous peer reviewers, author attribution should be to 'Anonymous Referee' followed by a clear attribution to the source work. The images or other third party material in this file are included in the article's Creative Commons license, unless indicated otherwise in a credit line to the material. If material is not included in the article's Creative Commons license and your intended use is not permitted by statutory regulation or exceeds the permitted use, you will need to obtain permission directly from the copyright holder. To view a copy of this license, visit http://creativecommons.org/licenses/by/4.0/.

Editorial Note: This manuscript has been previously reviewed at another journal that is not operating a transparent peer review scheme. This document only contains reviewer comments and rebuttal letters for versions considered at Nature Communications.

REVIEWERS' COMMENTS

Reviewer #1 (Remarks to the Author):

Electrosynthesis of chlorine from seawater-like solution through single-atom catalysts

This manuscript was initially submitted to Nature Synthesis and based on the referee comments, it got transferred to Nature Communications. I must say that the authors have dedicatedly revised their manuscript and addressed all my comments to full extent. Therefore, I recommend publication of their work in Nature Communications.

Reviewer #3 (Remarks to the Author):

I have now read the rebuttal letter as well as the revision version of the article, and based on this I can conclude that the authors have seriously considered my advices, and have also added a lot of additional data/experiments/explanations thereby providing more evidence for the different proposals/hypothesis made. Hence, based on this I am now positive about the article and recommend it for publication as it is.
